# Models, implementation, and reported outcomes of social prescribing interventions: a scoping review

**DOI:** 10.3389/fpubh.2026.1871347

**Published:** 2026-07-22

**Authors:** Adnan Kisa, Sezer Kisa

**Affiliations:** 1 Faculty of Health Sciences and Technology, Kristiania University of Applied Sciences, Oslo, Norway; 2 Department of International Health and Sustainable Development, Tulane University, New Orleans, LA, United States; 3 Faculty of Health Sciences, Department of Nursing and Health Promotion, Oslo Metropolitan University, Oslo, Norway

**Keywords:** community health services, health promotion, loneliness, mental health, primary health care, social prescribing, social support, well-being

## Abstract

**Background:**

Social prescribing has gained international attention as an approach that connects individuals with community-based, non-clinical resources to address social determinants of health and support well-being. Despite growing use across health and community systems, the evidence remains fragmented, with variation in intervention models, settings, target populations, mechanisms, and implementation conditions.

**Objective:**

This scoping review aimed to map the characteristics of social prescribing interventions, including models, initiation and coordination settings, referred community assets, target populations, reported mechanisms, outcomes, implementation facilitators and barriers, and evidence gaps.

**Methods:**

A scoping review was conducted following the frameworks of Arksey and O’Malley (22), Levac et al. (23) and Joanna Briggs Institute guidance. Reporting followed PRISMA-ScR and PRISMA-S. MEDLINE, Scopus, Web of Science Core Collection, APA PsycINFO, CINAHL, and Embase were searched from inception, with the final search conducted on June 13, 2026. Eligibility criteria followed the Population–Concept–Context framework. Two reviewers independently screened records and charted data using a standardized extraction form. Findings were synthesized descriptively and thematically.

**Results:**

Of 7,602 records identified, 115 peer-reviewed empirical studies met the inclusion criteria after deduplication and screening. Included studies used qualitative (*n* = 51), mixed-methods (*n* = 34), and quantitative (*n* = 30) designs and were conducted across primary care, community, cultural, nature-based, mental health, and hospital settings in 16 countries, including one cross-national Italy–Portugal study. Most studies originated from the United Kingdom (approximately 64%). Reported outcomes included improved mental well-being, reduced loneliness, increased confidence, stronger social connectedness, and improved everyday functioning, whereas clinical and system-level outcomes were more variable. Common mechanisms included relational continuity, personalized support, identity development, and engagement in meaningful community-based activities. Barriers included socioeconomic disadvantage, unstable funding, limited community-sector capacity, workforce pressures, and limited integration of social determinants into routine care pathways. Evidence gaps were notable for migrants, minoritized populations, young people, and economic evaluation.

**Conclusion:**

This review integrates evidence on link worker models, arts and cultural prescribing, nature-based approaches, and condition-focused programs within a single analytical framework. Social prescribing appears to operate through relational processes, community capacity, and local implementation conditions, not referral pathways alone. These findings may inform future research, policy, and practice.

## 1 Introduction

Health and well-being are shaped by the social, economic, and environmental conditions in which people live, and not solely by biomedical factors (1, 2). Many health systems continue to face persistent challenges related to loneliness, financial strain, housing insecurity, poor mental well-being, and limited opportunities for community participation, all of which are associated with poorer health outcomes and increased demand for health and social care (3–5). These unmet social and practical needs contribute to avoidable illness, reduced quality of life, and pressure on services that are primarily designed to respond to clinical problems. Because conventional clinical encounters often lack the time, resources, or community links needed to address these wider determinants, interest has grown in approaches that connect individuals with community-based support. Social prescribing (SP) has emerged as one such approach, connecting people with non-clinical resources intended to enhance social connection, daily functioning, and overall well-being (6, 7).

SP is commonly described as an umbrella term for community-linked programs that provide personalized support. Broad categories include link worker models, creative and cultural activities, nature-based initiatives, opportunities for learning or volunteering, and practical assistance with social or welfare needs (8, 9). Although these approaches differ in format and delivery, they collectively aim to complement clinical care by connecting individuals with community-based support. Existing reviews of nature-based interventions, programs for older adults, and community mental health initiatives illustrate the breadth of modalities and populations encompassed by SP, as well as the diversity of contexts in which such programs are implemented (10–13).

SP is not a single standardized intervention but a family of approaches that vary in intensity, referral pathway, professional involvement, and relationship with community assets. A useful way to characterize this diversity is provided by a three-tier social identity framework, which situates SP along a continuum from upstream, largely incidental forms to downstream, explicitly purposive forms (14). At the upstream end, Tier 1 comprises community initiatives and social infrastructure, such as libraries, parks, community centers, and neighborhood activities, that build social connection at a population level, often as a by-product of everyday community life rather than as a targeted health intervention. Tier 2 consists of group programs directed at specific populations or needs, in which agencies or practitioners connect individuals to structured group activities intended to address social disconnection. At the downstream end, Tier 3 involves person-centered interventions in which an individual prescriber, such as a link worker, community navigator, or community connector, works directly with a single client who often presents with complex and long-standing needs. These tiers are interdependent rather than mutually exclusive, and a single community asset may function simultaneously as a community initiative and as a site for targeted group activity. In some settings, particularly in Southern Europe and Latin America, related approaches may be described using different terminology, including assets recommendation, formal recommendation of community resources, health assets, or asset-based community approaches. These differences in terminology reflect variation in health system organization, primary care traditions, and the role of community health structures across countries.

Building on this conceptual and operational diversity, SP has seen rapid international adoption, supported by national strategies, investment in community assets, and policies that strengthen collaboration across health and social systems. Programs are now documented in primary care and community settings and across a range of health service pathways in several countries (15–18). This growth has stimulated work on program development and feasibility, including efforts to integrate referral processes with community provision (19). In this review, SP is therefore understood not only as an individual referral mechanism but also as part of broader community health action, requiring intersectoral collaboration, local governance, accessible community assets, and attention to whether programs reduce or reproduce social inequalities. Conceptual analyses further indicate that theoretical frameworks are applied inconsistently across studies, contributing to variation in how SP is interpreted and evaluated (20). As a result, the evidence base has expanded rapidly but remains diffuse and heterogeneous.

Variation is evident in how SP programs are structured, described, and assessed, with differences in referral pathways, delivery settings, target populations, and outcome measures. Reviews of nature-based activities, mental health promotion initiatives, and programs for older adults report considerable variability in program components and reporting practices, while a recent mapping review identified marked differences in outcome selection and measurement across countries (11, 13, 21). Although these reviews provide valuable insights, they typically focus on specific modalities, populations, or conceptual aspects of SP rather than offering an integrated synthesis across diverse program models and contexts (10, 12, 13, 21).

This diversity is also reflected in the scope of existing reviews, which often examine discrete domains such as nature-based initiatives, programs for older adults, theoretical frameworks, or specific outcome categories, thereby providing only partial insights into the broader field. What remains lacking is a comprehensive synthesis that maps how SP models are conceptualized, implemented, and evaluated across diverse health and community settings. As SP continues to expand internationally, a comprehensive evidence map capturing the range of program types, contextual features, and study designs is needed to support conceptual clarity and inform policy and practice.

This review synthesizes peer-reviewed international research to describe the types of SP programs and related non-medical referral pathways reported in the literature, the settings in which they are initiated or coordinated, the community assets to which people are referred, the populations involved, and the outcomes, mechanisms, and contextual factors examined. By assembling these elements into a single evidence map, the review aims to clarify how SP is currently conceptualized and evaluated and to inform future research, policy, and practice. The following research questions guide the review:

1. What types of SP interventions have been developed?
2. In which health care, social care, and community settings are these interventions initiated or coordinated, and to which community assets are individuals referred?
3. Which populations are targeted by SP initiatives?
4. What health, social, and system-level outcomes are reported in relation to these interventions?
5. What implementation facilitators, barriers, and contextual factors influence implementation and sustainability?
6. What evidence gaps and priorities exist for future research, policy, and practice?

## 2 Methods

### 2.1 Study design

This scoping review followed the methodological framework originally outlined by Arksey and O’Malley (22), further refined by Levac et al. (23), and guided by the Joanna Briggs Institute (JBI) Manual for Scoping Reviews (24). A scoping review approach was selected because SP literature is heterogeneous in study design, intervention types, and outcome measures, making evidence mapping more appropriate than effectiveness synthesis. The protocol for this scoping review was registered on the Open Science Framework (OSF) and is publicly available at: https://osf.io/zjmrp.

In accordance with JBI guidance, no critical appraisal of included studies was conducted (25), as the purpose of a scoping review is to map the breadth, characteristics, and distribution of existing evidence rather than to assess methodological quality or estimate intervention effectiveness. Variations in study rigor are therefore acknowledged and may limit the strength of conclusions regarding outcomes and mechanisms. All stages of the review, including research question development, evidence identification, study selection, data charting, and synthesis, were conducted in accordance with these methodological standards.

### 2.2 Eligibility criteria

Eligibility criteria were structured using the Joanna Briggs Institute Population–Concept–Context (PCC) framework.

#### 2.2.1 Population

##### 2.2.1.1 Inclusion

Studies involving individuals or groups who participated in, were referred to, or were eligible for SP interventions. This included, for example, older adults, people with chronic conditions, socially isolated individuals, and those experiencing mental health challenges.

##### 2.2.1.2 Exclusion

Studies that did not involve human participants, as well as articles that presented conceptual discussion without empirical data.

#### 2.2.2 Concept

##### 2.2.2.1 Inclusion

Empirical studies examining SP or related non-medical referral pathways that linked individuals to community-based support. Eligible interventions included arts on prescription, green or nature-based programs, link worker or community navigator models, and structured community referral pathways.

##### 2.2.2.2 Exclusion

Articles that did not examine SP or related non-medical referral interventions connecting individuals with community-based resources.

#### 2.2.3 Context

Studies were eligible if they were conducted in health care, social care, public health, community, educational, cultural, nature-based, or other community-linked settings where social prescribing interventions or related non-medical referral pathways were implemented. For eligibility and data charting, we distinguished between the setting in which social prescribing was initiated or coordinated and the community assets or resources to which individuals were referred.

Eligible initiation or coordination settings included primary care, general practice, public health services, mental health services, social care services, community organizations, schools, or educational settings when linked to health or well-being referral pathways, hospitals, emergency departments, and other health-related services. Eligible community assets included cultural venues, arts programs, green and blue spaces, physical activity programs, social groups, welfare or practical support services, voluntary and community organizations, and other non-clinical resources intended to support health, well-being, social connection, or daily functioning. No restrictions were applied by country or region.

Contexts were excluded if they were unrelated to health, well-being, social care, community care, or non-medical referral pathways, such as school-only or corporate programs without a health, well-being, community-care, or social prescribing component.

#### 2.2.4 Types of evidence sources and language restrictions

Eligible studies included peer-reviewed empirical research articles using qualitative, quantitative, mixed-methods, experimental, quasi-experimental, observational, implementation, and evaluation designs. Studies were eligible if they examined real-world social prescribing practices, models, pathways, implementation processes, or reported outcomes across health care, social care, community, cultural, nature-based, and other non-medical referral pathways. No geographic restrictions were applied.

Secondary literature, including systematic reviews, scoping reviews, narrative reviews, and umbrella reviews, was excluded from the evidence synthesis. Relevant reviews were used only for contextualization and targeted citation checking and were not treated as included evidence sources.

Grey literature, dissertations, policy reports, editorials, commentaries, conference abstracts, protocols, and other non-peer-reviewed materials were excluded. Only English-language peer-reviewed research journal articles were included. No date restrictions were applied. All eligible peer-reviewed research articles available up to the updated search date of June 13, 2026, were considered for inclusion.

### 2.3 Information sources

A comprehensive literature search was conducted in six bibliographic databases: PubMed/MEDLINE, Embase via Ovid, CINAHL via EBSCOhost, APA PsycINFO, Web of Science Core Collection, and Scopus. These databases were selected to capture biomedical, public health, social science, psychological, nursing, allied health, and interdisciplinary literature relevant to social prescribing and related non-medical referral pathways.

All retrieved records were exported to EndNote (Clarivate), where duplicate records were removed using automated and manual procedures. The deduplicated records were then imported into Rayyan for title and abstract screening. Grey literature was excluded to maintain a focused and comparable evidence base of peer-reviewed empirical studies and to support consistency in study identification, screening, and data charting. This decision may have underrepresented the community sector, local government, and practice-based evidence, particularly because many social prescribing initiatives are evaluated outside academic publishing.

### 2.4 Search strategy

The search strategy was developed by the review authors and refined through pilot searches to improve sensitivity and relevance. Additional synonyms and related terms were added to reflect variation in terminology and implementation across countries and settings. The strategy was adapted for each database to balance sensitivity with specificity for social prescribing and related non-medical referral pathways. Searches combined free-text terms with controlled vocabulary were supported by the database interface. In PubMed/MEDLINE, the Medical Subject Headings (MeSH) term for social prescribing was combined with title and abstract terms. In Embase, the relevant Emtree term was combined with title, abstract, and keyword terms. In CINAHL, the relevant subject heading was combined with title and abstract terms. APA PsycINFO, Web of Science Core Collection, and Scopus were searched using free-text terms in title, abstract, topic, or keyword fields, according to database functionality.

Free-text terms included social prescribing, social prescription, arts on prescription, green prescribing, nature prescribing, nature-based prescription, link workers, community connectors, community navigators, care navigators, well-being coordinators, and non-medical or non-clinical referral pathways. To improve international coverage, the updated search also incorporated terminology used in Southern European and Latin American contexts, including asset recommendation, assets recommendation, health assets, assets for health, asset-based approaches, health asset-based approaches, formal recommendation of community resources, community resource recommendation, and related non-medical community referral terms. The PubMed/MEDLINE strategy served as the base strategy for adaptation across databases and is presented in Supplementary file 1.

No date restrictions, study-design filters, or geographic restrictions were applied. Limits to English-language records and journal articles were applied where supported by each database interface. Each strategy was adapted to the indexing system, field codes, and syntax of the respective database. Search sensitivity was assessed using sentinel article testing. A set of highly cited empirical studies representing different social prescribing models, study designs, and geographic contexts from six countries was identified in advance and used to assess retrieval performance. All sentinel articles were retrieved through the combined database searches. The original search was conducted on October 1, 2025. An updated search was conducted on June 13, 2026, prior to resubmission, and all records available up to that updated search date were eligible for screening.

### 2.5 Selection of sources of evidence

All records were screened in Rayyan after automated and manual deduplication. The screening criteria were pilot tested on a subset of records to improve consistency among reviewers. Two reviewers independently screened titles and abstracts using predefined eligibility criteria, followed by full-text assessment of potentially eligible articles. No discrepancies or disagreements requiring adjudication occurred during title/abstract screening or full-text assessment. Any minor uncertainties were resolved through discussion and clarification of the eligibility criteria. The study selection process is presented in a PRISMA 2020 flow diagram. Following peer-review comments, two studies retrieved through the database searches were re-examined and assessed at full text against the predefined eligibility criteria. Both were incorporated into the final synthesis, and the PRISMA 2020 flow diagram was updated accordingly.

### 2.6 Data charting process

A standardized data charting form was developed in Microsoft Excel and piloted on four studies to assess consistency, completeness, and usability. Minor revisions were made before full data extraction began. These revisions included clarification of categories for intervention type, referral pathway, initiation or coordination setting, community assets or resources, target population, equity-relevant characteristics, outcome domains, theoretical frameworks, implementation facilitators, implementation barriers, and contextual factors.

Two reviewers independently charted the data using the predefined extraction form. Any minor uncertainties about categorization were resolved through discussion and clarification of the charting categories. Extracted data included:

- Author, year, and country
- Study aim, design, and methodology
- Initiation or coordination setting
- Community assets or resources to which individuals were referred
- Population characteristics and equity-relevant information, where reported
- Type of social prescribing intervention, such as link worker, arts-based, green prescribing, nature-based, community connector, or other non-medical referral model
- Referral pathway and delivery characteristics, where reported
- Theoretical framework, where reported
- Duration or intensity of the intervention, where reported
- Outcomes assessed, including health, social, and system-level outcomes
- Findings related to reported outcomes, implementation facilitators, and implementation barriers
- Contextual insights, mechanisms, system-level implementation issues, and policy-relevant observations

Data charting followed an iterative process, allowing categories to be refined as new themes emerged during extraction while maintaining consistency with the predefined eligibility criteria and research questions.

### 2.7 Synthesis of results

A combined descriptive and thematic synthesis approach was used. Because the aim of the review was to map the breadth and characteristics of the literature rather than to estimate pooled effects, no meta-analysis or inferential statistical testing across studies was undertaken. Quantitative findings were summarized using narrative synthesis, descriptive statistics, and frequency counts, including the distribution of intervention types, initiation or coordination settings, referred community assets, target populations, and reported outcomes. Qualitative findings were analyzed inductively to identify recurring patterns related to mechanisms, contextual determinants, implementation challenges, and implementation facilitators.

Findings were organized into the following analytical domains: types of social prescribing interventions; target populations; initiation or coordination settings; referred community assets; implementation processes and contextual determinants; health, social, and system-level outcomes; mechanisms and pathways; implementation facilitators and barriers; and evidence gaps and implications for future research, policy, and practice. Equity-related considerations, including socioeconomic disadvantages, cultural and linguistic accessibility, digital exclusion, transport barriers, and differential engagement across population groups, were charted where reported. Reporting followed the Preferred Reporting Items for Systematic Reviews and Meta-Analyses extension for Scoping Reviews (PRISMA-ScR) guidelines (Checklist 1) (26), and search reporting was guided by the PRISMA extension for reporting literature searches (PRISMA-S) (27).

## 3 Results

**Figure 1:**
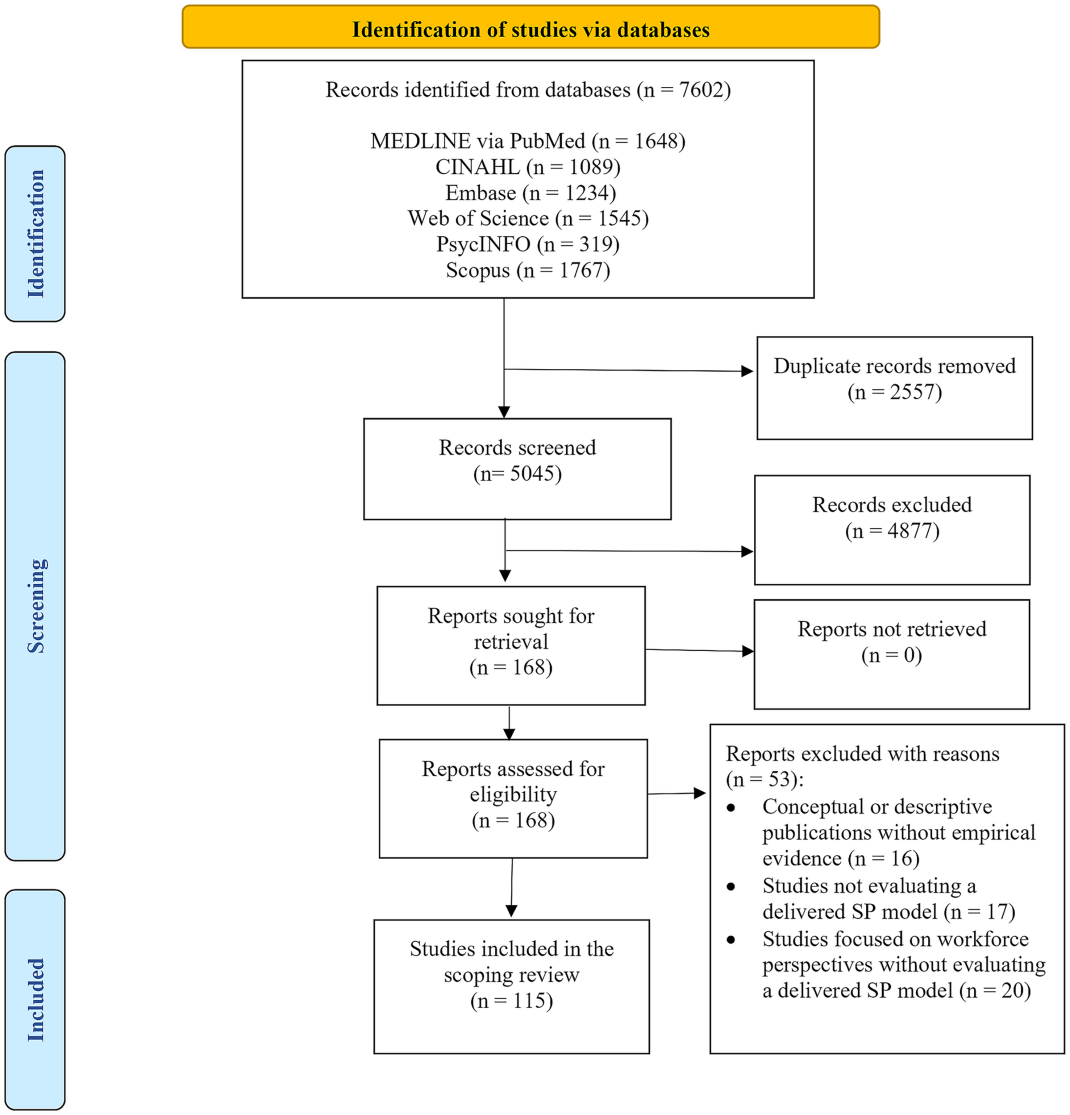
PRISMA 2020 flow diagram of the study selection process.

A total of 7,602 records were identified across six databases. After removing 2,557 duplicates, 5,045 titles and abstracts were screened, and 168 full-text articles were assessed for eligibility. Of these, 53 were excluded for predefined reasons (e.g., no SP intervention or non-health-related context), resulting in 115 studies included in the final review. The study selection process is illustrated in a PRISMA 2020 flow diagram (Figure 1). Detailed characteristics of all included studies are summarized in Supplementary Table 1, while intervention models, outcomes, implementation facilitators, implementation barriers, and contextual factors across studies are synthesized in Supplementary Table 2.

### 3.1 Characteristics of the included studies

#### 3.1.1 Study designs

Qualitative research comprised the largest proportion of included studies (*n* = 51), followed by mixed methods studies (*n* = 34) and quantitative designs (*n* = 30). This distribution reflects the exploration and implementation-focused nature of the current evidence base.

#### 3.1.2 Geographic distribution

The evidence base remains concentrated in the United Kingdom, which accounted for approximately 64% of all included studies (*n* = 74). Beyond the United Kingdom, the largest contributors were Australia (*n* = 7), Canada (*n* = 5), and the United States (*n* = 5), followed by Sweden (*n* = 4) and Denmark, Portugal, and Ireland (*n* = 3 each). Two studies each were identified from the Netherlands and Spain, and single studies were identified from France, Germany, Italy, Japan, New Zealand, and South Korea, with one additional cross-national study involving both Italy and Portugal. Although the updated search broadened the international evidence base, this continued concentration in the United Kingdom limits the generalizability of the findings to other health systems. Studies conducted in England, Scotland, and Northern Ireland were categorized under the United Kingdom for analytical purposes.

#### 3.1.3 Populations of the included studies

The included studies represented a broad range of population groups. The most frequently studied populations were adults experiencing mental health challenges, chronic conditions, loneliness, or multimorbidity. Studies also examined older adults, people living with dementia, individuals with complex psychosocial or socioeconomic needs, and culturally diverse communities. Some studies focused on specific groups such as unpaid carers and young people, including individuals not in education, employment, or training (NEET).

#### 3.1.4 Focus of the included studies

Included studies mainly fell into four broad, non-mutually exclusive areas of focus: implementation and process studies; evaluations of health and social outcomes; population-specific SP models; and studies that explicitly used theoretical frameworks. Implementation and process studies examined referral mechanisms, mechanisms of change as described by participants, and stakeholder experiences across primary care, community, and voluntary-sector settings. Outcome-focused studies reported health and social outcomes, including well-being, psychological distress, loneliness, chronic condition management, and health or social care utilization. Population-specific studies examined models for groups such as people living with dementia, young people, including those NEET, unpaid carers, culturally diverse communities, and people with complex psychosocial needs. A smaller body of studies used theoretical frameworks, including Self-Determination Theory, the Social Cure model, realist evaluation approaches, and Critical Systems Thinking, to explain how SP may generate change. This distribution indicates that the field has mainly prioritized descriptive and process-oriented evidence, with a smaller but growing body of theory-informed outcome research.

### 3.2 Outcomes of SP

Across the included studies, authors reported a wide range of outcomes associated with SP, spanning psychosocial, social, clinical, and system-related domains.

Psychosocial outcomes were the most consistently reported. Quantitative evaluations reported reductions in loneliness, psychological distress, anxiety, and depression, as well as increases in well-being, patient activation, physical activity, daily self-management, and quality of life (15, 28–38). Qualitative studies reported changes in coping, emotional regulation, confidence, resilience, autonomy, and sense of purpose (39–50), alongside outcomes related to meaningful activity, creative engagement, and emotional expression in arts, culture, or nature-based programs (51–55). Studies involving groups with complex psychosocial needs and people living with dementia also reported improvements in programs providing ongoing or adapted forms of support (51, 55, 56).

Social outcomes were reported across study designs and included increased social support, broader social networks, participation in community or cultural activities, and a stronger sense of belonging or identity (41, 44, 57–63).

Clinical and system outcomes showed greater variability. Several studies reported changes in clinical indicators, including frailty, glycated hemoglobin (HbA1c), and other health-related measures, in programs that included lifestyle or behavior change components (32, 56, 64–68). System-related findings were mixed: some evaluations reported reductions in general practice consultations, changes in care coordination, or positive social return on investment (69–75), while others found little or no change in healthcare utilization, including general practice visits, hospital attendance, or medication use (29, 31, 76–78). A number of studies reported limited or negative system-related outcomes, particularly in settings characterized by socioeconomic disadvantage, unstable housing, acute mental health needs, reduced program capacity, or COVID-19-related disruptions (59, 78, 79). Additional system-level outputs included variation in service reach, program fidelity, session volume, data completeness, and referral pathway activity.

### 3.3 Types of SP interventions

SP interventions included generalist and specialist models delivered through health, community, and voluntary sector structures (Supplementary Table 2). The most prevalent model involved link workers or coordinators who conducted holistic assessments and facilitated connections to community or social resources (15, 18, 29, 31, 35, 39, 69, 74, 80, 81). Generalist pathways addressed a range of social, emotional, economic, and lifestyle-related needs. Specialist models were developed for specific populations and settings, including hospital discharge or hospital-linked pathways for socially deprived inpatients, children with neurodisability, and older patients (16, 35, 72), dementia-oriented models (51, 55), child and youth connector models (49, 62), and Armed Forces Community-specific models (50, 82).

Arts and cultural prescribing interventions included performing arts, museum visits, heritage engagement, and writing programs (36, 48, 52, 53, 83–86). Nature-based interventions included gardening, conservation activities, walking and cycling groups, outdoor creative programs, mindfulness sessions, and community horticulture (33, 34, 54, 61, 63, 71, 75, 79, 87–89). Condition-focused and lifestyle-related interventions included structured programs for diabetes and cardiovascular risk, gym or physical activity referral schemes, and electronic referral pathways linked to clinical systems (32, 64–68, 89, 90). Community-based models also included Men’s Sheds and community library services (91, 92).

### 3.4 Implementation settings and referred community assets

In this review, implementation settings refer to the health, social care, or community systems through which SP was initiated, coordinated, or managed, whereas community assets refer to the non-clinical resources, activities, and venues to which individuals were referred. This distinction is important because the point of initiation was often located in formal care systems, while the intervention itself frequently depended on community-based participation and support.

Primary care was the most frequently reported implementation setting, with link workers or coordinators based in general practices, primary care networks, or comparable community-facing health service structures. Delivery formats included in-person and hybrid approaches and often involved coordination with multidisciplinary teams, local authorities, and voluntary-sector partners (15, 18, 29, 37, 39, 57, 81, 93). Hospital-linked SP pathways included discharge coordination for socially deprived inpatients in France (16), a hospital in-reach family-centered model for children with neurodisability (72), and a hospital-based program for older patients (35). Other secondary and mental health-related settings included dementia-oriented, community mental health, and integrated care pathways (51, 94, 95).

Referred community assets included community centers, leisure facilities, cafes, libraries, Men’s Sheds, places of worship, arts and cultural venues, green spaces, and nature-based settings. One school-based example was the Kids in Parks TRACK Rx nature prescription program, in which a school nurse prescribed outdoor physical activity and TRACK Trails to children in rural elementary schools (89). Specific examples included cultural prescribing pathways involving museums, galleries, theatres, and heritage sites (7, 52, 53, 84, 86), nature-based programs involving gardens, woodlands, coastal areas, farms, walking groups, and horticulture activities (34, 59, 61, 71, 79, 86, 87), and community-based resources such as libraries, voluntary-sector organizations, and social groups (91, 92, 96). Differentiating implementation settings from referred community assets clarifies where SP is coordinated, where support is delivered, and which local resources are required for transferability across health systems.

### 3.5 Target populations

SP was delivered to diverse population groups, with adults experiencing multimorbidity, long-term conditions, frailty, or complex psychosocial needs being the most commonly reported recipients (29, 31, 39, 68, 80, 97). Older adults were frequently represented, including those with cognitive impairment or dementia, bereavement, functional limitations, or caregiving roles (7, 28, 35, 37, 38, 51, 52, 57, 69, 88, 93, 98–100). Mental health-related presentations included depression, anxiety, stress, social withdrawal, low self-esteem, and post-traumatic symptoms (8, 33, 36, 40, 48, 53, 70, 83, 87, 101–106). Loneliness and social isolation were reported across age groups, linked to bereavement, disability, reduced mobility, retirement, or limited community engagement (9, 15, 28, 37, 38, 63, 69, 75, 76, 98, 107–110). Ethnic minority and migrant populations were also represented, including multilingual groups and specific cultural communities such as Pakistani carers, and some studies also described outreach to refugees and asylum seekers (17, 43, 61, 63, 84, 111, 112). Additional groups included unpaid carers, people with disabilities or learning difficulties, and young people with mental health needs, NEET status, or limited social connectedness (8, 15, 42, 43, 47–49, 62, 80, 92, 106, 108, 113–115). Hospital-linked pathways involved people attending emergency departments, those experiencing homelessness or socioeconomic hardship, and individuals with cardiovascular, metabolic, or lifestyle-related risks, alongside referrals addressing financial strain, caregiving burden, housing challenges, and low well-being (17, 32, 35, 67, 72, 83, 114, 116–119). Several studies also documented socioeconomic disadvantages, including poverty, housing instability, debt, digital exclusion, and limited-service access (41, 58, 59, 91).

### 3.6 Implementation facilitators

The most consistently reported implementation facilitators across studies were the quality of the practitioner relationship, flexibility in delivery format, and the availability of appropriate community assets. Person-centered tailoring, holistic assessment, and flexible approaches, including in-person, home-based, community-based, and remote delivery, were frequently noted (8, 28, 45, 55, 98, 118, 120). Therapeutic relationship features, including empathy, time, trust, motivational interviewing, and continuity with link workers or other SP practitioners, were described as central to engagement across diverse populations and settings (29, 39, 40, 43, 47, 50, 57, 69, 82, 112, 121).

At the system level, general practitioner endorsement, co-location of link workers within primary care, access to medical records, structured referral pathways, and clear communication between sectors were identified as important implementation facilitators (29, 39, 57, 60, 81, 114, 117, 122). Collaboration across primary care, community organizations, cultural and arts institutions, voluntary-sector organizations, and faith-based groups supported referral quality and continuity of support (43, 52, 53, 84, 92, 121). For marginalized and minority groups, cultural and linguistic alignment, peer or buddy systems, and providers with shared lived experience were particularly important (16, 41, 43, 50, 58, 61, 63, 108). For example, green SP for ethnically diverse, migrant, refugee, and asylum-seeking communities relied on trusted community organizations as culturally meaningful routes into green and blue spaces (61), while Armed Forces-specific SP highlighted the importance of cultural competence, shared understanding of military experience, advocacy, and veteran-specific service navigation (50, 82). Organizational factors, including well-defined role boundaries, supervision, reflective practice, protected staff time, training, and adaptable implementation structures, were also reported as supporting implementation (18, 46, 78, 94, 122, 123). In some contexts, prior community health structures and established intersectoral relationships appeared to function not only as implementation facilitators but as enabling conditions for feasible and sustainable implementation.

### 3.7 Barriers to SP

Barriers to SP were reported at multiple levels, with individual-level obstacles, limited community capacity, and system fragmentation emerging as cross-cutting themes (Supplementary Table 2). Patient-level barriers included caring responsibilities, limited time, fluctuating health, psychological distress, low confidence, stigma, socioeconomic hardship, transport difficulties, limited digital access, literacy and language challenges, cultural mismatch, and limited readiness to engage in community activities (45, 78, 83, 118, 120).

Provider-level barriers included limited awareness of SP pathways, restricted appointment times, inconsistent knowledge of community resources, variation in referral quality, high caseloads, emotional burden, and workforce turnover, which affected relational continuity (40, 46, 57, 121). However, a biomedical focus should not be understood only as an individual provider-level barrier. At the system level, a predominantly biomedical and reactive care model limited the integration of social determinants of health into routine referral and care pathways (40, 57, 121).

Organizational and system-level barriers included fragmented coordination between sectors, unclear role expectations, limited information sharing, short-term or siloed funding arrangements, variation in program duration and eligibility criteria, limited voluntary-sector capacity, inconsistent data systems, and limited access to outcome measurement tools (57, 79, 81, 93, 95, 111, 117). Community-level barriers included limited availability and accessibility of local activities, unstable provision, cultural inappropriateness of some services, volunteer shortages, and broader structural factors such as poverty, housing insecurity, transport gaps, and neighborhood safety. COVID-19 restrictions introduced additional barriers, including reduced community capacity and increased digital exclusion (78, 79, 96, 124). These barriers suggest that SP may be least accessible to some groups with the greatest social needs unless implementation is supported by adequate community infrastructure, outreach, and equity-oriented design.

### 3.8 Contextual factors

Implementation and delivery of SP were shaped by a complex set of structural, policy, and local contextual conditions, with socioeconomic deprivation and system capacity constraints emerging as the most reported influences across studies (Supplementary Table 2). Many programs operated in deprived or multicultural areas characterized by poverty, unemployment, multimorbidity, loneliness, and migration-related diversity, conditions that influenced both the types of needs encountered and the organization of services (17, 29, 43, 61, 73, 80, 125). Structural pressures, including reductions in community-sector capacity, unstable commissioning arrangements, uneven community infrastructure, and high workload within primary care, were noted across multiple settings (40, 57, 121).

Policy and governance environments introduced further variation through differences in referral pathways, commissioning structures, data sharing systems, and regional or national priorities. Several studies identified the use of theoretical frameworks, including Self-Determination Theory, Social Cure approaches, and salutogenic perspectives, to inform program design (9, 41, 49, 93, 116, 126). Geographic conditions such as rural isolation, limited transport, seasonal constraints, and uneven access to green spaces were reported in settings including the United Kingdom and Japan (91, 118, 122). The COVID-19 pandemic introduced additional contextual variation through shifts to remote or hybrid delivery, reduced voluntary sector capacity, increased socioeconomic pressures, and higher levels of digital exclusion (32, 78, 96, 109, 111, 112, 124). Program maturity and the history of local intersectoral collaboration were also relevant: areas with established links between general practice and the voluntary sector reported more coherent operational processes, while regions with emerging or fragmented systems described ongoing coordination challenges (52, 76, 117).

### 3.9 Evidence gaps and future research priorities

**Figure 2:**
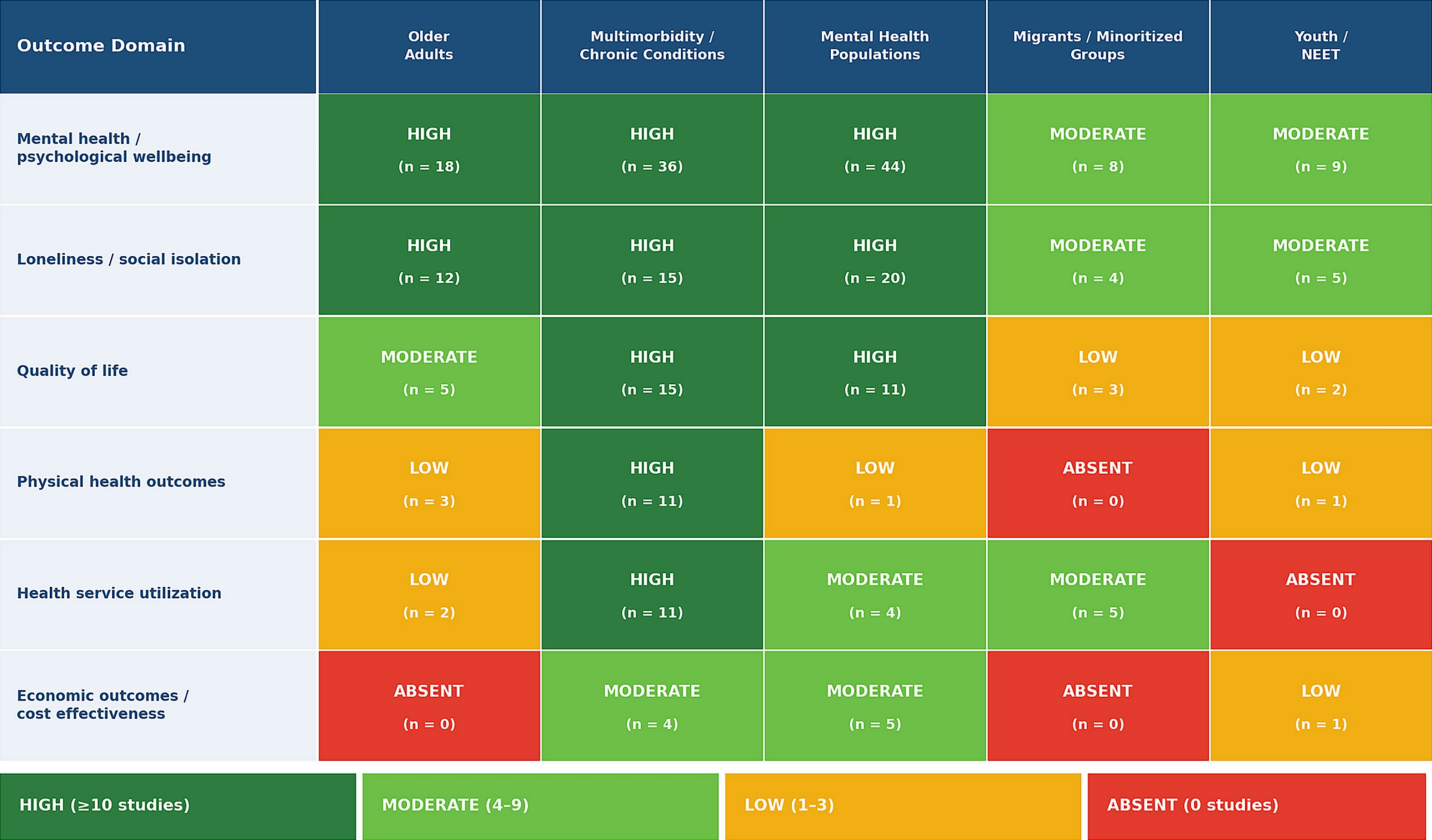
Evidence gap map of SP studies by population group and outcome domain. The figures shows the distribution of included studies (*n* = 115) across five population groups and six outcome domains. Evidence levels were categorized as HIGH (≥10 studies), MODERATE (4–9 studies), LOW (1–3 studies), and ABSENT (0 studies). Numbers indicate the count of studies reporting each outcome within each population group. Because many studies reported more than one out comes or population group, totals across cells exceed the total number of included studies. The map highlights areas with substantial evidence, such as mental health and loneliness outcomes among older adults, populations with chronic conditions, and mental health populations, and areas where evidence remains limited, including economic and physical health outcomes among migrant, minoritized, and youth populations. The visualization was prepared in accordance with guidance for scoping reviews (24, 25).

To visually summarize the distribution of evidence across population groups and outcome domains, an evidence gap map was developed (Figure 2). The map summarizes the extent to which different outcomes have been investigated among major population groups represented in the included studies, including older adults, individuals with multimorbidity or chronic conditions, mental health populations, migrants or minoritized groups, and youth or individuals NEET. Evidence levels were categorized as high (≥10 studies), moderate (4–9 studies), low (1–3 studies), or absent (0 studies). Because individual studies frequently reported multiple outcomes or included multiple population groups, a single study may contribute to multiple cells in the map. The map visually indicates areas where evidence is more frequently reported, particularly for psychosocial outcomes, as well as areas where fewer studies were identified, such as economic outcomes and studies involving migrants, minoritized populations, or youth.

The reviewed studies identified gaps in the SP evidence base that can be organized into four principal categories (Supplementary Table 2). First, methodological limitations constrained causal inference. Limited large-scale and longitudinal research was frequently noted, restricting understanding of long-term effectiveness, dosage requirements, and outcome variation across groups such as carers, young people, migrants, older adults, people with multimorbidity, and people living with dementia (28, 37, 43, 47, 51, 62, 77, 80, 127). Causal inference was further limited by the scarcity of randomized or quasi-experimental designs, short follow-up periods, varied comparator groups, and inconsistent outcome measures. Routine data systems did not consistently capture relational, contextual, or structural factors reported to influence engagement and outcomes (39, 83, 105).

Second, mechanistic understanding remained incomplete. Uncertainty was reported regarding the roles of autonomy support, identity, confidence, social belonging, relational work, cultural adaptation, and broader contextual conditions in shaping participation and outcomes (41, 110, 128, 129). Several studies noted the need for more precise specification of program components, link worker roles, fidelity expectations, and monitoring systems that reflect relational and contextual variation, alongside workforce considerations such as training, supervision, and retention (53, 121, 122).

Third, economic and system-level evidence was limited. Short-term funding arrangements, unstable commissioning models, and constrained voluntary sector capacity contributed to the small number of studies that included economic evaluation or long-term modeling (59, 71, 72, 75, 79).

Fourth, equity-related gaps were prominent. These included limited availability of culturally tailored or co-designed models, few examinations of non-engagement and refusal, and limited analysis of socioeconomic and gender-related variation. Structural barriers such as poverty, transport availability, digital access, and community resource distribution were identified as insufficiently examined in relation to access and outcomes (43, 56, 61, 63, 88, 115, 120).

## 4 Discussion

### 4.1 Principal findings

This review mapped evidence on SP models, initiation and coordination settings, referred community assets, populations, outcomes, implementation facilitators, implementation barriers, and evidence gaps. The findings show that SP is most often described as a relational and context-dependent public health practice, with psychosocial outcomes reported more consistently than clinical or system-level effects. The evidence base remains heavily concentrated in the United Kingdom, and the results should therefore be interpreted with caution when considering transferability to other health systems and contexts. Previous reviews have examined particular populations, intervention types, mechanisms, implementation issues, or outcome domains. This review adds to that literature by bringing these strands together across SP models, referral and coordination settings, community assets, population-specific patterns, equity-related barriers, and reported health, social, and system outcomes. The following sections discuss these patterns in relation to reported outcomes, mechanisms, implementation conditions, population-specific findings, implications for commissioners, and limitations of the current evidence base.

#### 4.1.1 Psychosocial outcomes and mechanisms

Psychosocial improvement was the most consistently reported outcome across the included studies. Reductions in loneliness, anxiety, and emotional strain were commonly accompanied by increases in confidence, motivation, routine, social connection, and sense of purpose. These effects were observed across diverse models, including link worker programs, arts and cultural activities, nature-based interventions, community groups, and physical activity prescriptions, suggesting that conditions enabling connection, structure, and meaning may be more influential than the specific activity type. Most studies using validated mental health or well-being scales reported improvement, although measurement tools and follow-up periods varied considerably.

These patterns are consistent with findings from reviews of arts and nature-based interventions (13, 30, 99, 109, 130, 131). The current review complements that evidence by mapping relational processes that were commonly proposed or described as contributing to reported outcomes across a broader range of SP models and populations. Qualitative and mixed-method studies consistently highlighted relational work, including sustained listening, emotional validation, culturally sensitive communication, and practical support, as a recurring process linked to perceived change. These practices were described as supporting autonomy and competence by helping individuals clarify priorities, overcome barriers, and engage with community resources. Increased participation and restored routine were then associated with improved mood and reduced loneliness, consistent with Self-Determination Theory (132) and behavioral activation frameworks (133).

Although rarely measured quantitatively, some studies reported identity shifts, with individuals moving from illness-focused or socially withdrawn roles toward positions as contributors or group members, consistent with social identity approaches to well-being (14, 47, 49). This relational pathway aligns with social identity accounts of SP, which propose that benefits arise when individuals join and come to identify with meaningful groups, supported across community, group-program, and person-centered tiers of delivery (14). These findings point to a plausible pathway in which relational continuity, emotional safety, and motivational support are associated with psychosocial benefit, although this pathway remains insufficiently tested in comparative or longitudinal designs. These findings are also consistent with recent social identity-informed work on SP, including Staras et al. (134) systematic review, which emphasizes social connection, group belonging, and identity-related mechanisms. The present review differs by mapping these mechanisms across a broader range of SP models, populations, initiation and coordination settings, referred community assets, and outcome domains, rather than focusing specifically on social identity processes.

The psychosocial benefits documented in this review are consistent with broader public health evidence on social support and well-being. Perceived social support, particularly self-esteem support, has been associated with improved quality of life among individuals with cardiovascular disease, highlighting the potential relevance of relational mechanisms similar to those emphasized in SP interventions (135). Likewise, perceived social support has been associated with psychological well-being in the post-COVID period, a context in which several studies in this review described disruptions to SP delivery and increased social isolation (136).

#### 4.1.2 Clinical and system outcomes

Clinical and system outcomes showed greater variability than psychosocial findings, and causality cannot be inferred from the available evidence. Improvements in frailty, physical activity, chronic disease indicators, and self-management were most reported in programs with sustained engagement, structured follow-up, or explicit integration with primary care and community organizations (29, 32, 67, 68, 73). Shorter or less coordinated models rarely demonstrated comparable gains.

System-level findings were mixed across populations, program models, and implementation conditions. Some evaluations reported modest reductions in primary or emergency care use among individuals with high baseline utilization who received extended relational support (73, 74), but several studies reported no change or noted temporary increases as previously unmet needs were identified and addressed. These findings are consistent with previous mapping reviews (21, 131, 137) and suggest that any system-level effects are likely to depend on program duration, intensity of relational support, baseline risk, infrastructure stability, and integration within care pathways. Psychosocial outcomes were reported more consistently than reductions in service utilization and claims about cost savings should be made cautiously based on current evidence.

Conceptual work on SP evaluation also highlights the methodological challenges of assessing interventions within complex and evolving health systems. A stage-sensitive evaluation framework proposed for SP integrates implementation science, realist evaluation, and theory-informed approaches to better capture how and under what conditions such interventions produce outcomes (138). This perspective reinforces the need for evaluation strategies that account for contextual variation, program maturity, and system complexity when interpreting findings from SP research.

### 4.2 Implementation and context

SP delivery appeared most coherent where service design included clear referral pathways, well-defined practitioner roles, regular cross-sector communication, protected supervision time, and sufficient community-sector capacity (43, 81, 84, 121). Programs operating within multisector networks involving primary care, voluntary and community organizations, cultural institutions, green space initiatives, social care structures, and local authorities reported stronger user engagement and more sustained delivery (8, 43, 84, 121, 139). In this context, multisector networks refer to structured relationships between health services, social care, local government, voluntary-sector organizations, cultural providers, and community groups that support referral, navigation, and sustained participation.

These patterns suggest that SP is not simply an individual referral intervention but part of a broader process of community health action. In settings where intersectoral collaboration, local governance structures, and established relationships between health services and community organizations were already in place, SP appeared easier to embed and sustain. Prior community health structures may therefore function not only as implementation facilitators but also as enabling conditions for feasible and sustainable delivery.

Many programs nevertheless faced persistent challenges related to role ambiguity, communication gaps between sectors, limited community capacity, short-term funding arrangements, fragmented data systems, and high turnover among link workers (57, 79, 81, 93, 95, 121). These conditions disrupted relational continuity and affected engagement. The evidence reinforces that implementation context cannot be separated from outcomes (12, 140) and that sustainability depends on stable infrastructure, workforce continuity, professional training, and strong intersectoral collaboration (46, 60, 121, 122). Future implementation studies should therefore specify the content, format, and competencies covered in training for prescribers, link workers, and community-sector partners, including supervision models, continuing professional development, and strategies for translating person-centered and equity-oriented SP theory into routine practice.

With respect to implementation, the findings align with prior reviews of social prescribing, which have highlighted referral-pathway clarity, link worker role definition, workforce support, and voluntary and community-sector capacity as recurring determinants of delivery (46, 81, 118). Donaghy et al. (141) showed that link workers in England and Scotland increasingly support patients with complex needs related to the social determinants of health, particularly in areas of socioeconomic deprivation, while short-term funding, role ambiguity, rising demand, and pressure on statutory and voluntary services threatened sustainability. Potter et al. (142) showed that COVID-19 disrupted social prescribing delivery through reduced voluntary-sector capacity, digital exclusion, loss of in-person contact, referral backlogs, and escalating complexity of need. The present review adds to this literature by situating these implementation issues within a broader cross-model synthesis that also considers initiation and coordination settings, community assets, target populations, reported outcomes, and equity-related implementation barriers.

Differences in primary care organization are also important for interpreting transferability. Much of the included evidence came from the United Kingdom, where SP has often been developed through primary care networks, link-worker roles, and commissioning arrangements. In health systems with different primary care structures, stronger or weaker longitudinal relationships with patients, different community health traditions, or more formalized intersectoral governance, implementation requirements may differ. Wider conceptual and policy literature similarly indicates growing international interest in SP and related community referral approaches, while also showing substantial variation in program structures, referral mechanisms, and funding arrangements (143). Terminological variation across English-language SP traditions and related Southern European and Latin American concepts, such as asset recommendation, formal recommendation of community resources, and health asset-based approaches, may also influence evidence retrieval, interpretation, and cross-system comparison. Reported implementation challenges, including fragmented referral pathways, limited resources, and the absence of standardized evaluation frameworks, are consistent with the patterns identified in this review. This interpretation is consistent with recent conceptual models that frame SP as a community referral approach dependent on local infrastructure, intersectoral partnerships, and system readiness (144).

### 4.3 Population-specific patterns and equity

Population-specific patterns were evident across the included studies but were often insufficiently analyzed in depth. For older adults, tailoring activities to mobility, accessibility preferences, and social circumstances was associated with enhanced connection and mood, consistent with earlier reviews (10–12). Dementia-focused programs relied on caregiver involvement, structured and adaptable activities, and relational continuity (51, 55). Migrants and minoritized groups faced language, trust, and cultural barriers, but engagement was reported to improve where multilingual communication, culturally proximate link workers, and community-based outreach were available (16, 43, 58, 61, 63, 112). Young people not in education, employment, or training benefited from relational support integrated with identity building and future-oriented planning (47–49, 62, 106, 113). In multimorbidity-focused programs, SP appeared to function as a bridge supporting navigation, lifestyle change, and emotional support alongside clinical care (29, 140).

Equity concerns were widespread and should be considered central to SP implementation rather than peripheral. Individuals experiencing poverty, housing instability, limited transport, digital exclusion, language barriers, or low trust in services faced substantial challenges in accessing or sustaining engagement. Strategies including transport vouchers, flexible scheduling, refreshments, multilingual materials, culturally tailored communication, peer link workers, and outreach through trusted community organizations were reported to improve engagement but were inconsistently available due to resource constraints (43, 51, 58, 61, 63, 111).

These findings raise concerns about the inverse care law in SP implementation, whereby those with the greatest needs may be least able to access or benefit from services unless programs are deliberately designed to reduce structural barriers (145). Programs that rely heavily on self-referral, digital communication, stable transport, or well-developed local community assets may disproportionately reach individuals who are already better positioned to engage, while underserving those with the greatest social and structural needs. The evidence suggests that achieving equitable access to SP may require structural action beyond the health sector, including welfare, housing, transport, digital inclusion, community development, and urban planning (41, 58, 59, 120). Without such conditions, SP risks reinforcing rather than reducing existing inequities (1, 2).

The evidence gap map developed in this review further illustrates the uneven distribution of research across population groups and outcome domains. While psychosocial outcomes among older adults and individuals with chronic conditions were frequently examined, substantially fewer studies addressed economic outcomes or populations such as migrants, minoritized groups, and youth. These patterns highlight important gaps in the current evidence base and suggest that future research should prioritize underrepresented populations, equity-relevant outcomes, non-engagement, refusal, and differential access to better understand how SP can contribute to equitable health and social care delivery across diverse contexts.

### 4.4 Implications for health service and social care commissioners

The findings have practical implications for health service and social care commissioners. Social prescribing should not be commissioned simply as a referral pathway or as a low-cost alternative to clinical or statutory services. Across the included studies, implementation depended on the infrastructure surrounding the referral itself: the availability of voluntary and community-sector provision, clear referral routes, support for link workers and prescribers, and sustained coordination between primary care, social care, local government, public health, and community organizations (57, 79, 81, 93, 95, 121, 141).

A central implication is the need to protect the relational work on which social prescribing often depends. Reported benefits were frequently linked to person-centered assessment, time to build trust, continuity of contact, and practical support for people with complex social and health needs. Service specifications that focus mainly on referral volume or short-term throughput may therefore be poorly aligned with how social prescribing appears to work in practice. More supportive commissioning would allow for realistic caseloads, supervision, training, role clarity, and sufficient contact time for link workers and prescribers (18, 46, 78, 94, 122, 123, 141).

The capacity of the voluntary, community, and social enterprise sector is also central. Social prescribing relies on accessible community assets, but several studies showed that short-term funding, volunteer shortages, reduced community provision, and unstable local infrastructure can limit both implementation and continuity of support (57, 79, 81, 121, 142). Commissioning link worker posts without also considering the capacity of the organizations receiving referrals risks weakening the pathway and limiting its ability to address social isolation, practical needs, and community participation.

Equity also needs to be built into commissioning decisions. People experiencing poverty, housing insecurity, transport barriers, digital exclusion, language barriers, or low trust in services may face the greatest difficulty accessing or sustaining engagement with social prescribing. Commissioners therefore need to consider practical supports such as outreach, transport assistance, multilingual and culturally tailored provision, digital inclusion, and partnerships with trusted community organizations, particularly in deprived or underserved communities (43, 51, 58, 61, 63, 111).

Finally, outcome measurement should reflect the aims and mechanisms of social prescribing. Evaluation based only on referral counts or short-term health service use is unlikely to capture the full value or limitations of these interventions. Commissioners may need to include relational, social, equity-related, and community-level outcomes, such as loneliness, confidence, social connectedness, participation, well-being, access to community assets, and sustainability of community-sector provision (39, 93, 105, 141). These data would help identify who is reached, where gaps remain, and where additional investment or redesign may be needed.

### 4.5 Limitations

Several limitations of this review should be acknowledged. First, as a scoping review, no formal critical appraisal of included studies was conducted, consistent with the purpose of a scoping review and JBI guidance. Consequently, studies with varying methodological rigor were synthesized together, and conclusions regarding outcomes should be interpreted as reported findings rather than as causally established effects. Second, the methodological profile of the evidence base was dominated by qualitative, mixed-methods, and observational designs, which limit causal inference and contribute to variation in reported outcomes. Many studies relied on small or selective samples and used heterogeneous outcome measures, further restricting direct comparisons across intervention models. Third, the geographic distribution of the literature was heavily concentrated in the United Kingdom, which accounted for the majority of included studies. Evidence from other countries remained limited, and research from low-resource or underrepresented health-system contexts was scarce. This concentration limits the generalizability of the findings to wider international contexts. Fourth, the exclusion of grey literature may have resulted in the underrepresentation of community and voluntary-sector perspectives. Because many SP programs are implemented and delivered by community organizations, excluding non-peer-reviewed sources may have skewed the evidence toward models embedded in formal health care systems. Fifth, substantial heterogeneity across study designs, populations, settings, and outcome measures limited the ability to directly compare intervention models or to synthesize outcomes quantitatively. Finally, the search was restricted to peer-reviewed English-language studies. Although an updated search was conducted prior to resubmission, relevant studies published in other languages, indexed outside the searched databases, or reported in grey literature may not be represented. Despite these limitations, the broad scope of this review enabled a comprehensive mapping of SP models, initiation and coordination settings, referred community assets, mechanisms, and population-specific patterns across a diverse body of literature.

## 5 Conclusion

This scoping review provides an integrated cross-model synthesis of SP interventions across diverse settings, populations, and delivery models. The findings suggest that SP may be most usefully understood not as a fixed program model or transactional referral mechanism, but as a relational and context-dependent public health practice shaped by structural, organizational, and community conditions. By synthesizing intervention types, initiation and coordination settings, referred community assets, population needs, mechanisms, implementation facilitators, implementation barriers, and evidence gaps across 115 studies, this review offers a broader understanding of how SP operates within health and community systems.

The review brings together evidence on link worker models, arts and cultural prescribing, nature-based interventions, and condition-focused programs within a single analytical framework. It also moves beyond outcome description by mapping potential mechanisms of change and the contextual conditions under which SP appears to generate benefit. Distinguishing initiation and coordination settings from the referred community assets strengthens the interpretation of how SP is organized, coordinated, and delivered across different systems. The synthesis also shows that implementation factors, equity considerations, and evidence gaps need to be considered alongside outcome findings when assessing the relevance of SP for policy and practice.

Across the reviewed studies, psychosocial outcomes were reported more consistently than clinical or system-level effects, suggesting that the benefits of SP are most strongly associated with relational support, community participation, and engagement in meaningful activities. Feasible and sustainable implementation appears to depend on relational continuity, person-centered assessment, strong coordination between health services and community organizations, and the availability of appropriate community assets. Adequate training, supervision, and protected time for prescribers, link workers, and community-sector partners, together with stable commissioning arrangements and investment in community-sector capacity, are likely important for maintaining program stability and equitable access.

Future research should prioritize longitudinal and comparative study designs that clarify causal pathways, intervention components, and implementation conditions. Greater attention is also needed to economic evaluation, health equity, culturally tailored or co-designed models, non-engagement, and implementation in regions beyond the United Kingdom, where the current evidence base remains limited.

Realizing the public health potential of SP will depend not only on referral mechanisms but also on sustained investment in the relational, community, and structural conditions that enable individuals to engage meaningfully with the social determinants of health.
