# Supplementary material for: Models, implementation, and reported outcomes of social prescribing interventions: a scoping review
Source: Front Public Health. 2026 Jul 22;14:1871347. doi: 10.3389/fpubh.2026.1871347 (PMC13437648; doi:10.3389/fpubh.2026.1871347)
Supplement: Supplementary file 1 [file Supplementary_file_1.docx]

**Supplementary File 1. Full Database Search Strategies**

Updated search: 13 June 2026. Original search: 1 October 2025.

Limits applied where supported by the interface: English language; journal articles.

No date restrictions and no study-design or geographic filters were applied.

The PubMed/MEDLINE strategy served as the base strategy and was adapted to each database's indexing system, field codes, and syntax. The number of records retrieved is reported beneath each strategy.

**1. PubMed/MEDLINE**

Controlled vocabulary (MeSH) combined with title/abstract free-text terms.

(

"Social Prescribing"[Mesh]

OR "social prescribing"[tiab]	OR "social prescription"[tiab]	OR "social prescriptions"[tiab]	OR "arts on prescription"[tiab]	OR "art on prescription"[tiab]	OR "culture on prescription"[tiab]	OR "cultural prescription"[tiab]	OR "green prescribing"[tiab]	OR "green prescription"[tiab]	OR "nature prescribing"[tiab]	OR "nature prescription"[tiab]	OR "nature-based prescription"[tiab]	OR "nature based prescription"[tiab]	OR "community referral"[tiab]	OR "community referrals"[tiab]	OR "community resource referral"[tiab]	OR "community resource referrals"[tiab]	OR "community-based referral"[tiab]	OR "community based referral"[tiab]	OR "non-medical referral"[tiab]	OR "non-medical referrals"[tiab]	OR "non medical referral"[tiab]	OR "non medical referrals"[tiab]	OR "non-clinical referral"[tiab]	OR "non-clinical referrals"[tiab]	OR "non clinical referral"[tiab]	OR "non clinical referrals"[tiab]	OR "non-clinical support"[tiab]	OR "non clinical support"[tiab]	OR "link worker"[tiab]	OR "link workers"[tiab]	OR "community link worker"[tiab]	OR "community link workers"[tiab]	OR "social prescribing link worker"[tiab]	OR "social prescribing link workers"[tiab]	OR "community connector"[tiab]	OR "community connectors"[tiab]	OR "community navigator"[tiab]	OR "community navigators"[tiab]	OR "care navigator"[tiab]	OR "care navigators"[tiab]	OR "wellbeing coordinator"[tiab]	OR "wellbeing coordinators"[tiab]	OR "well-being coordinator"[tiab]	OR "well-being coordinators"[tiab]

OR (

(

"asset recommendation"[tiab]	OR "asset recommendations"[tiab]	OR "assets recommendation"[tiab]	OR "assets recommendations"[tiab]	OR "health asset"[tiab]	OR "health assets"[tiab]	OR "assets for health"[tiab]	OR "asset-based approach"[tiab]	OR "asset-based approaches"[tiab]	OR "asset based approach"[tiab]	OR "asset based approaches"[tiab]	OR "health asset-based approach"[tiab]	OR "health asset-based approaches"[tiab]	OR "health asset based approach"[tiab]	OR "health asset based approaches"[tiab]	OR "asset-based community development"[tiab]	OR "asset based community development"[tiab]	OR "formal recommendation of community resources"[tiab]	OR "recommendation of community resources"[tiab]	OR "community resource recommendation"[tiab]	OR "community resource recommendations"[tiab]	OR "formal recommendation of assets for health"[tiab]	OR "recommendation of assets for health"[tiab]

)

AND

(

referral*[tiab]	OR prescrib*[tiab]	OR prescription*[tiab]	OR recommendation*[tiab]	OR "primary care"[tiab]	OR "general practice"[tiab]	OR "health service"[tiab]	OR "health services"[tiab]	OR "community resource"[tiab]	OR "community resources"[tiab]	OR "community-based resource"[tiab]	OR "community based resource"[tiab]	OR "non-medical"[tiab]	OR "non medical"[tiab]	OR "non-clinical"[tiab]	OR "non clinical"[tiab]

)

)

)

AND english[lang]

AND journal article[pt]

**Records retrieved: 1,648.**

**2. Embase via Ovid**

Emtree controlled vocabulary combined with free-text terms in the title, abstract, and keyword fields. Ovid search-set numbering begins at 1.

**1.**	exp social prescribing/

**2.**	("social prescribing" or "social prescription" or "social prescriptions" or "arts on prescription" or "art on prescription" or "culture on prescription" or "cultural prescription" or "green prescribing" or "green prescription" or "nature prescribing" or "nature prescription" or "nature-based prescription" or "nature based prescription" or "community referral" or "community referrals" or "community resource referral" or "community resource referrals" or "community-based referral" or "community based referral" or "non-medical referral" or "non-medical referrals" or "non medical referral" or "non medical referrals" or "non-clinical referral" or "non-clinical referrals" or "non clinical referral" or "non clinical referrals" or "non-clinical support" or "non clinical support" or "link worker" or "link workers" or "community link worker" or "community link workers" or "social prescribing link worker" or "social prescribing link workers" or "community connector" or "community connectors" or "community navigator" or "community navigators" or "care navigator" or "care navigators" or "wellbeing coordinator" or "wellbeing coordinators" or "well-being coordinator" or "well-being coordinators").ti,ab,kw.

**3.**	("asset recommendation" or "asset recommendations" or "assets recommendation" or "assets recommendations" or "health asset" or "health assets" or "assets for health" or "asset-based approach" or "asset-based approaches" or "asset based approach" or "asset based approaches" or "health asset-based approach" or "health asset-based approaches" or "health asset based approach" or "health asset based approaches" or "asset-based community development" or "asset based community development" or "formal recommendation of community resources" or "recommendation of community resources" or "community resource recommendation" or "community resource recommendations" or "formal recommendation of assets for health" or "recommendation of assets for health").ti,ab,kw.

**4.**	(referral* or prescrib* or prescription* or recommendation* or "primary care" or "general practice" or "health service" or "health services" or "community resource" or "community resources" or "community-based resource" or "community based resource" or "non-medical" or "non medical" or "non-clinical" or "non clinical").ti,ab,kw.

**5.**	1 or 2 or (3 and 4)

**6.**	limit 5 to english language

**7.**	limit 6 to article

**Records retrieved: 1,234.**

**3. CINAHL via EBSCOhost**

CINAHL subject heading combined with title (TI) and abstract (AB) free-text terms.

(

(MH "Social Prescribing+")

OR TI ("social prescribing" OR "social prescription" OR "social prescriptions" OR "arts on prescription" OR "art on prescription" OR "culture on prescription" OR "cultural prescription" OR "green prescribing" OR "green prescription" OR "nature prescribing" OR "nature prescription" OR "nature-based prescription" OR "nature based prescription" OR "community referral" OR "community referrals" OR "community resource referral" OR "community resource referrals" OR "community-based referral" OR "community based referral" OR "non-medical referral" OR "non-medical referrals" OR "non medical referral" OR "non medical referrals" OR "non-clinical referral" OR "non-clinical referrals" OR "non clinical referral" OR "non clinical referrals" OR "non-clinical support" OR "non clinical support" OR "link worker" OR "link workers" OR "community link worker" OR "community link workers" OR "social prescribing link worker" OR "social prescribing link workers" OR "community connector" OR "community connectors" OR "community navigator" OR "community navigators" OR "care navigator" OR "care navigators" OR "wellbeing coordinator" OR "wellbeing coordinators" OR "well-being coordinator" OR "well-being coordinators")

OR AB ("social prescribing" OR "social prescription" OR "social prescriptions" OR "arts on prescription" OR "art on prescription" OR "culture on prescription" OR "cultural prescription" OR "green prescribing" OR "green prescription" OR "nature prescribing" OR "nature prescription" OR "nature-based prescription" OR "nature based prescription" OR "community referral" OR "community referrals" OR "community resource referral" OR "community resource referrals" OR "community-based referral" OR "community based referral" OR "non-medical referral" OR "non-medical referrals" OR "non medical referral" OR "non medical referrals" OR "non-clinical referral" OR "non-clinical referrals" OR "non clinical referral" OR "non clinical referrals" OR "non-clinical support" OR "non clinical support" OR "link worker" OR "link workers" OR "community link worker" OR "community link workers" OR "social prescribing link worker" OR "social prescribing link workers" OR "community connector" OR "community connectors" OR "community navigator" OR "community navigators" OR "care navigator" OR "care navigators" OR "wellbeing coordinator" OR "wellbeing coordinators" OR "well-being coordinator" OR "well-being coordinators")

OR (

(TI ("asset recommendation" OR "asset recommendations" OR "assets recommendation" OR "assets recommendations" OR "health asset" OR "health assets" OR "assets for health" OR "asset-based approach" OR "asset-based approaches" OR "asset based approach" OR "asset based approaches" OR "health asset-based approach" OR "health asset-based approaches" OR "health asset based approach" OR "health asset based approaches" OR "asset-based community development" OR "asset based community development" OR "formal recommendation of community resources" OR "recommendation of community resources" OR "community resource recommendation" OR "community resource recommendations" OR "formal recommendation of assets for health" OR "recommendation of assets for health") OR AB ("asset recommendation" OR "asset recommendations" OR "assets recommendation" OR "assets recommendations" OR "health asset" OR "health assets" OR "assets for health" OR "asset-based approach" OR "asset-based approaches" OR "asset based approach" OR "asset based approaches" OR "health asset-based approach" OR "health asset-based approaches" OR "health asset based approach" OR "health asset based approaches" OR "asset-based community development" OR "asset based community development" OR "formal recommendation of community resources" OR "recommendation of community resources" OR "community resource recommendation" OR "community resource recommendations" OR "formal recommendation of assets for health" OR "recommendation of assets for health"))

AND

(TI (referral* OR prescrib* OR prescription* OR recommendation* OR "primary care" OR "general practice" OR "health service" OR "health services" OR "community resource" OR "community resources" OR "community-based resource" OR "community based resource" OR "non-medical" OR "non medical" OR "non-clinical" OR "non clinical") OR AB (referral* OR prescrib* OR prescription* OR recommendation* OR "primary care" OR "general practice" OR "health service" OR "health services" OR "community resource" OR "community resources" OR "community-based resource" OR "community based resource" OR "non-medical" OR "non medical" OR "non-clinical" OR "non clinical"))

)

)

AND LA English

AND PT "Journal Article"

**Records retrieved: 1,089.**

**4. APA PsycINFO via EBSCOhost**

APA PsycINFO was searched using free-text terms in the title (TI) and abstract (AB) fields; no dedicated social-prescribing descriptor was used.

(

TI ("social prescribing" OR "social prescription" OR "social prescriptions" OR "arts on prescription" OR "art on prescription" OR "culture on prescription" OR "cultural prescription" OR "green prescribing" OR "green prescription" OR "nature prescribing" OR "nature prescription" OR "nature-based prescription" OR "nature based prescription" OR "community referral" OR "community referrals" OR "community resource referral" OR "community resource referrals" OR "community-based referral" OR "community based referral" OR "non-medical referral" OR "non-medical referrals" OR "non medical referral" OR "non medical referrals" OR "non-clinical referral" OR "non-clinical referrals" OR "non clinical referral" OR "non clinical referrals" OR "non-clinical support" OR "non clinical support" OR "link worker" OR "link workers" OR "community link worker" OR "community link workers" OR "social prescribing link worker" OR "social prescribing link workers" OR "community connector" OR "community connectors" OR "community navigator" OR "community navigators" OR "care navigator" OR "care navigators" OR "wellbeing coordinator" OR "wellbeing coordinators" OR "well-being coordinator" OR "well-being coordinators")

OR AB ("social prescribing" OR "social prescription" OR "social prescriptions" OR "arts on prescription" OR "art on prescription" OR "culture on prescription" OR "cultural prescription" OR "green prescribing" OR "green prescription" OR "nature prescribing" OR "nature prescription" OR "nature-based prescription" OR "nature based prescription" OR "community referral" OR "community referrals" OR "community resource referral" OR "community resource referrals" OR "community-based referral" OR "community based referral" OR "non-medical referral" OR "non-medical referrals" OR "non medical referral" OR "non medical referrals" OR "non-clinical referral" OR "non-clinical referrals" OR "non clinical referral" OR "non clinical referrals" OR "non-clinical support" OR "non clinical support" OR "link worker" OR "link workers" OR "community link worker" OR "community link workers" OR "social prescribing link worker" OR "social prescribing link workers" OR "community connector" OR "community connectors" OR "community navigator" OR "community navigators" OR "care navigator" OR "care navigators" OR "wellbeing coordinator" OR "wellbeing coordinators" OR "well-being coordinator" OR "well-being coordinators")

OR (

(TI ("asset recommendation" OR "asset recommendations" OR "assets recommendation" OR "assets recommendations" OR "health asset" OR "health assets" OR "assets for health" OR "asset-based approach" OR "asset-based approaches" OR "asset based approach" OR "asset based approaches" OR "health asset-based approach" OR "health asset-based approaches" OR "health asset based approach" OR "health asset based approaches" OR "asset-based community development" OR "asset based community development" OR "formal recommendation of community resources" OR "recommendation of community resources" OR "community resource recommendation" OR "community resource recommendations" OR "formal recommendation of assets for health" OR "recommendation of assets for health") OR AB ("asset recommendation" OR "asset recommendations" OR "assets recommendation" OR "assets recommendations" OR "health asset" OR "health assets" OR "assets for health" OR "asset-based approach" OR "asset-based approaches" OR "asset based approach" OR "asset based approaches" OR "health asset-based approach" OR "health asset-based approaches" OR "health asset based approach" OR "health asset based approaches" OR "asset-based community development" OR "asset based community development" OR "formal recommendation of community resources" OR "recommendation of community resources" OR "community resource recommendation" OR "community resource recommendations" OR "formal recommendation of assets for health" OR "recommendation of assets for health"))

AND

(TI (referral* OR prescrib* OR prescription* OR recommendation* OR "primary care" OR "general practice" OR "health service" OR "health services" OR "community resource" OR "community resources" OR "community-based resource" OR "community based resource" OR "non-medical" OR "non medical" OR "non-clinical" OR "non clinical") OR AB (referral* OR prescrib* OR prescription* OR recommendation* OR "primary care" OR "general practice" OR "health service" OR "health services" OR "community resource" OR "community resources" OR "community-based resource" OR "community based resource" OR "non-medical" OR "non medical" OR "non-clinical" OR "non clinical"))

)

)

AND LA English

AND PT "Journal Article"

**Records retrieved: 319.**

**5. Web of Science Core Collection**

Searched using the Topic (TS) field (title, abstract, author keywords, and Keywords Plus); no controlled subject thesaurus was used.

TS=(

"social prescribing" OR "social prescription" OR "social prescriptions" OR "arts on prescription" OR "art on prescription" OR "culture on prescription" OR "cultural prescription" OR "green prescribing" OR "green prescription" OR "nature prescribing" OR "nature prescription" OR "nature-based prescription" OR "nature based prescription" OR "community referral" OR "community referrals" OR "community resource referral" OR "community resource referrals" OR "community-based referral" OR "community based referral" OR "non-medical referral" OR "non-medical referrals" OR "non medical referral" OR "non medical referrals" OR "non-clinical referral" OR "non-clinical referrals" OR "non clinical referral" OR "non clinical referrals" OR "non-clinical support" OR "non clinical support" OR "link worker" OR "link workers" OR "community link worker" OR "community link workers" OR "social prescribing link worker" OR "social prescribing link workers" OR "community connector" OR "community connectors" OR "community navigator" OR "community navigators" OR "care navigator" OR "care navigators" OR "wellbeing coordinator" OR "wellbeing coordinators" OR "well-being coordinator" OR "well-being coordinators"

OR (

("asset recommendation" OR "asset recommendations" OR "assets recommendation" OR "assets recommendations" OR "health asset" OR "health assets" OR "assets for health" OR "asset-based approach" OR "asset-based approaches" OR "asset based approach" OR "asset based approaches" OR "health asset-based approach" OR "health asset-based approaches" OR "health asset based approach" OR "health asset based approaches" OR "asset-based community development" OR "asset based community development" OR "formal recommendation of community resources" OR "recommendation of community resources" OR "community resource recommendation" OR "community resource recommendations" OR "formal recommendation of assets for health" OR "recommendation of assets for health")

AND

(referral* OR prescrib* OR prescription* OR recommendation* OR "primary care" OR "general practice" OR "health service" OR "health services" OR "community resource" OR "community resources" OR "community-based resource" OR "community based resource" OR "non-medical" OR "non medical" OR "non-clinical" OR "non clinical")

)

)

Refined by: Language = English; Document type = Article

**Records retrieved: 1,545.**

**6. Scopus**

Searched using the TITLE-ABS-KEY field; no controlled subject thesaurus was used.

TITLE-ABS-KEY(

"social prescribing" OR "social prescription" OR "social prescriptions" OR "arts on prescription" OR "art on prescription" OR "culture on prescription" OR "cultural prescription" OR "green prescribing" OR "green prescription" OR "nature prescribing" OR "nature prescription" OR "nature-based prescription" OR "nature based prescription" OR "community referral" OR "community referrals" OR "community resource referral" OR "community resource referrals" OR "community-based referral" OR "community based referral" OR "non-medical referral" OR "non-medical referrals" OR "non medical referral" OR "non medical referrals" OR "non-clinical referral" OR "non-clinical referrals" OR "non clinical referral" OR "non clinical referrals" OR "non-clinical support" OR "non clinical support" OR "link worker" OR "link workers" OR "community link worker" OR "community link workers" OR "social prescribing link worker" OR "social prescribing link workers" OR "community connector" OR "community connectors" OR "community navigator" OR "community navigators" OR "care navigator" OR "care navigators" OR "wellbeing coordinator" OR "wellbeing coordinators" OR "well-being coordinator" OR "well-being coordinators"

OR (

("asset recommendation" OR "asset recommendations" OR "assets recommendation" OR "assets recommendations" OR "health asset" OR "health assets" OR "assets for health" OR "asset-based approach" OR "asset-based approaches" OR "asset based approach" OR "asset based approaches" OR "health asset-based approach" OR "health asset-based approaches" OR "health asset based approach" OR "health asset based approaches" OR "asset-based community development" OR "asset based community development" OR "formal recommendation of community resources" OR "recommendation of community resources" OR "community resource recommendation" OR "community resource recommendations" OR "formal recommendation of assets for health" OR "recommendation of assets for health")

AND

(referral* OR prescrib* OR prescription* OR recommendation* OR "primary care" OR "general practice" OR "health service" OR "health services" OR "community resource" OR "community resources" OR "community-based resource" OR "community based resource" OR "non-medical" OR "non medical" OR "non-clinical" OR "non clinical")

)

)

AND ( LIMIT-TO ( LANGUAGE , "English" ) )

AND ( LIMIT-TO ( DOCTYPE , "ar" ) )

**Records retrieved: 1,767.**

**Total records retrieved:**

PubMed/MEDLINE: 1,648

Embase: 1,234

CINAHL: 1,089

APA PsycINFO: 319

Web of Science Core Collection: 1,545

Scopus: 1,767

Total records retrieved across all six databases: 7,602. After removing 2,557 duplicates, 5,045 records were screened.
