# Supplementary material for: Models, implementation, and reported outcomes of social prescribing interventions: a scoping review
Source: Front Public Health. 2026 Jul 22;14:1871347. doi: 10.3389/fpubh.2026.1871347 (PMC13437648; doi:10.3389/fpubh.2026.1871347)
Supplement: Supplementary file 2 [file Table_1.docx]

**Supplementary Table 1. Characteristics of Included Studies and Summary of Key Findings**

| **Author(s), Year, Country** | **Study Design** | **Study Population** | **Study Aim** | **Key Findings** |
| --- | --- | --- | --- | --- |
| Aggar et al., 2025, Australia [15] | Mixed methods (pre-post intervention design using ONS4 well-being measures; semi-structured interviews) | n = 93 adult informal carers with mental health needs and/or chronic conditions enrolled in social prescribing programs | To assess the impact of a social prescribing program on quality of life, well-being, psychological distress, and healthcare utilization among adult carers. | Significant improvements were observed in quality of life, mental well-being, self-reported health, and psychological distress (all p < .001). GP visits reduced significantly (p = .002). Carers received more social prescriptions than non-carers (mean difference = 2.05). Remote telephone contact was the primary engagement modality (98%). |
| Ajibade et al., 2024, UK [39] | Mixed methods (online survey with Likert and free text responses; semi-structured interviews) | n = 102 GPs; n = 55 narrative responses; n = 8 interviews | To examine GP attitudes, experiences, perceptions, and challenges regarding social prescribing in primary care. | 90% of GPs reported using social prescribing and were broadly supportive. Principal components analysis identified six attitude components. Three qualitative themes emerged: perceived effectiveness, training and awareness, and structural and operational delivery. Implementation barriers included low awareness, limited training, poor communication and feedback, administrative burden, and underfunding. |
| Armitt et al., 2026, England [61] | Mixed-methods study using a pre-post intervention design with ONS4 well-being measures and qualitative semi-structured interviews | Quantitative component: n = 143 participants from ethnically diverse communities accessing green social prescribing activities through six community organizations in Hull and York, England; n = 103 consented to additional data collection, and n = 79 provided 6-month outcome data. Qualitative component: n = 20 interviews with participants, activity organizers, and one person who withdrew after one session. | To explore the impact and experience of green social prescribing activities for ethnically diverse, migrant, refugee, and asylum-seeking communities, and to understand the role of community organizations in supporting access and engagement. | After 6 months of engagement in green social prescribing, slight improvements were observed across all ONS4 well-being domains, with a statistically significant reduction in anxiety (mean change −0.7, 95% CI −1.4 to 0.01, p = 0.02). Around two-thirds of participants maintained or improved well-being across life satisfaction, feeling worthwhile, happiness, and anxiety. Qualitative findings showed that trusted community organizations were central to access and engagement, provided safe and culturally meaningful routes into green space, and supported social connection, belonging, heritage, culture, physical activity, relaxation, and mental well-being among underserved communities. |
| Aughterson, Baxter & Fancourt, 2020, UK [30] | Qualitative (one-to-one semi-structured interviews; reflexive thematic analysis) | n = 17 GPs from varied UK regions, practices, and career stages | To explore barriers and enablers influencing GP engagement in social prescribing for patients with mental health problems. | GPs viewed social prescribing as valuable for addressing psychosocial needs. Enablers included the link worker role, trust-based GP-patient relationships, practice-wide support, and feedback on patient benefit. Barriers included limited time, insufficient training, sustainability concerns in the voluntary and community sectors, high unmet need, and variability in patient motivation. |
| Rabinowitz Bailey and DiMilia, 2026, United States [36] | Quantitative (quasi-experimental single-group longitudinal study; secondary analysis of programmatic data and repeated WHO-5 Wellbeing Index assessments) | n = 240 adult Art Pharmacy participants from California and Georgia, United States; n = 239 included in the primary baseline-adjusted analysis. Participants were adults referred through healthcare, community, university, Medicaid, Medicare, mental health, cancer, and palliative care partners for loneliness, social isolation, depression, anxiety, or unmet mental health needs. | To examine changes in self-reported mental health and psychological well-being over time during participation in Art Pharmacy, a U.S.-based arts-based social prescribing program. | WHO-5 well-being scores increased significantly across all follow-up time points. In the baseline-adjusted model, scores increased by 5.5 points at follow-up 1 and peaked at approximately 18–20 points at follow-ups 6–8, exceeding the 10-point threshold for meaningful change. Improvements were sustained through follow-up 9. Similar trajectories were observed in demographic-adjusted sensitivity analyses. Age, race, ethnicity, and treatment duration were not significantly associated with follow-up well-being scores, although male participants showed higher WHO-5 scores in demographic-adjusted models. The findings provide preliminary longitudinal evidence that arts-based social prescribing may improve mental health and well-being in the U.S. context. |
| Bertotti et al., 2018, UK [57] | Mixed methods (realist evaluation; interviews, focus groups, surveys, and observations) | n = 17 service users; n = 3 SPCs; n = 2 commissioners; n = 2 GPs; n = 3 community organizations; GP survey n = 52 | To explore contextual factors and mechanisms shaping the implementation of a social prescribing pathway linking primary care with the voluntary sector. | Three pathway mechanisms were identified: GP referral driven by awareness and belief in non-clinical solutions; SPC-patient relationship, where trust and face-to-face contact supported behavior change; and engagement with community organizations, which reduced isolation and improved personal control over health. Challenges included limited GP engagement, short clinical consultations, unclear branding, and underfunded voluntary sector capacity. |
| Bertotti et al., 2025, Italy & Portugal [113] | Mixed methods (realist evaluation; pre-post cohort data with qualitative interviews) | n = 416 young people aged 16 to 34 in NEET situations; n = 161 followed up; interviews with young people, link workers, and C.O.P.E. team members | To evaluate mental health outcomes of the C.O.P.E. social prescribing intervention and identify contextual factors and mechanisms shaping outcomes for NEET youth. | Significant improvements in mental well-being and reductions in psychological distress were observed, including transitions from clinically significant to non-clinically significant states. Improvements were greatest among females, younger participants (aged 16 to 24), and those with poorer baseline mental health. Strengthened self-esteem, confidence, motivation, social skills, and re engagement in training and employment were reported. Trusting, flexible, and holistic link worker relationships were identified as core mechanisms. Challenges included sustainability, high time demands, and variable inter-service coordination. |
| Bhatti et al., 2021, Canada [41] | Qualitative (case study; focus groups and interviews) | n = 96 adults referred to social prescribing in 11 Community Health Centers | To explore how social prescribing facilitates positive outcomes through the lens of Self-Determination Theory. | Social prescribing was experienced as an autonomy supportive process that fostered trusted relationships and enhanced autonomy, competence, relatedness, and beneficence. Improvements in mental health, social support, community belonging, chronic condition self-management, and sense of purpose were reported. Benefits were particularly noted among participants who took on health champion volunteer roles. |
| Bishop-Edwards et al., 2026, England [124] | Qualitative (photo-elicitation focus groups; inductive thematic analysis) | n = 22 established members of VCSE-run creative and social groups supported by Creative Minds in South West Yorkshire, England; participants attended groups before, during, and after the COVID-19 lockdown. Groups were inclusive of people with serious mental health needs, learning disabilities, neurodiversity, and other marginalized experiences. | To explore group users' experiences of creative social prescribing groups during the COVID-19 lockdown, examine the impact of changes in group delivery on social connection, belonging, mental health, and recovery, and compare user perspectives with previous findings from group providers. | Participants emphasized that creative groups provided social connectedness, a sense of belonging, routine, friendship, peer support, creative identity, and a sense of acceptance. Online or adapted provision during lockdown helped maintain some connection but was generally viewed as a poor substitute for in-person groups. Lockdown magnified pre-existing marginalization, isolation, anxiety, and access barriers. Skilled, warm, compassionate, and responsive group leadership was central to maintaining connection and group cohesion. Findings highlighted the importance of inclusive, flexible, and hybrid community provision while preserving the value of in-person social prescribing groups. |
| Bos et al., 2025, Netherlands [58] | Qualitative (participatory action research; interviews and observations) | n = 26 participants: health and care professionals, experts-by-experience, and clients with complex multiple problems | To explore perspectives on improving support for people with complex multiple problems through social prescribing. | Participants identified a need for support beyond referral to existing activities. A tailored approach addressing wider determinants, single case manager coordination, culturally appropriate support, and strong interprofessional collaboration was identified as necessary. Key barriers included systemic fragmentation, unclear roles, limited time and resources, and misalignment between client needs and available services. |
| Brandborg et al., 2021, Denmark [64] | Qualitative (semi-structured interviews; Framework Method) | n = 9 Danish GPs with varied years in practice and practice locations | To explore Danish GPs' views on the advantages, disadvantages, facilitators, and barriers of implementing social prescribing to promote physical activity in primary care. | GPs were predominantly positive about social prescribing as a community-based, non-medical approach promoting physical activity and social well-being. Key barriers included patient expectations for medicalized treatment, stigma around mental and social issues, limited patient financial resources, GP time constraints, lack of systematic signposting tools, and concerns about third sector competency. |
| Brettell et al., 2022, UK [8] | Quantitative (pre-post outcome evaluation using routine data) | n = 14 to 21 young people aged 16 to 25 with social isolation or mental health issues | To evaluate the impact of the Linking Leeds social prescribing service on mental health and well-being among young people. | Improvements were observed in loneliness (71.4%), mental well-being (80.9%), and personal well-being (66.7%), with reduced support needs across life domains. Qualitative findings indicated that social connectedness and behavioral activation contributed to observed improvements. |
| Brown & Aylett, 2025, UK [87] | Qualitative (multi-stakeholder interviews and workshops) | n = 39 stakeholders involved in coordination, referral, or delivery of nature-based social prescribing | To explore how green social prescribing is conceptualized and experienced by stakeholders during the formalization of the Welsh national social prescribing framework. | Five themes were identified: lack of consensus on definitions and roles; tension regarding referrals and responsibilities; funding insecurity and short-term provision; lack of standardized evaluation metrics; and uncertainty about sustainability and system alignment. |
| Cailhol et al., 2024, France [16] | Quantitative (quasi-experimental quality improvement intervention) | n = 223 adult inpatients discharged from endocrinology and infectious disease departments in a disadvantaged area | To assess unmet social prescribing needs at hospital discharge and examine the impact of a personalized social prescribing plus discharge coordination intervention on unplanned rehospitalization. | 69.1% of patients needed help understanding discharge information; 43.9% required social prescribing support (language, housing, health literacy, finances). No significant difference in rehospitalization rates was observed (3.1% vs 2.6%). Extensive unmet social needs were identified, particularly in a high-immigrant population. |
| Calderón-Larrañaga et al., 2023, UK [17] | Mixed methods (realist evaluation; quantitative and qualitative components) | n = 447,360 adults in 35 GP practices; n = 41,378 at high risk of type 2 diabetes; qualitative subsample of SP users and practitioners | To evaluate how primary care based social prescribing contributes to type 2 diabetes prevention in a multi-ethnic, socioeconomically deprived population, and compare referral patterns with the NHS Diabetes Prevention Program. | Adults at high risk of type 2 diabetes were four times more likely to be referred to social prescribing than the general population (attenuated after adjustment). Social prescribing reached more deprived and multimorbid groups than the NHS Diabetes Prevention Program and was described as having broader eligibility and more sustained support. Social determinants (housing, debt, welfare, language) were addressed alongside lifestyle behaviors. Strong primary care and integration with the voluntary and community sector enabled timely cross-referrals and personalized support. |
| Calderon-Larrañaga et al., 2024, UK [111] | Qualitative (case study; interviews and ethnographic observations) | n = 35 participants, including primary care professionals, link workers, and VCS practitioners; observations in community SP activities | To investigate how social prescribing for adults at high risk of type 2 diabetes is implemented in primary care and community settings. | Three overlapping SP practice types were identified: reflective reinvention (creative, relationship-based tailoring); constrained provision (limited by eligibility rules, resource shortages, and workload pressures); and uncritical adoption (target-driven, protocol-focused SP). Creative practices were constrained by short-term funding and audit requirements. Policy narratives emphasizing independence were associated with constrained, compliant practices and limited personalized support for those with the greatest need. |
| Calderón-Larrañaga et al., 2026, England [67] | Quantitative (observational study using routinely collected national primary care data; cross-sectional and prospective cohort analyses) | n = 2,326,400 adults at high risk of type 2 diabetes from 1,839 general practices in England using Royal College of General Practitioners Research and Surveillance Centre data; 148,443 individuals were referred to social prescribing | To examine the reach, equity of access, and potential impact of social prescribing on type 2 diabetes prevention among adults at high risk | Social prescribing referrals increased 27-fold between 2019 and 2023. Referral was more common among women, older adults, people living in more deprived areas, current smokers, people with obesity, depression or anxiety, multimorbidity, and prior NHS Diabetes Prevention Program referral. Referral was slightly lower among Asian groups and younger adults, indicating equity gaps. Over a median of 3.7 years, 271,582 individuals developed type 2 diabetes. Social prescribing was initially associated with higher diabetes risk during early roll-out but became protective by 2023 (aHR 0.84, 95% CI 0.79–0.89). Benefit also emerged after ≥3 years post-referral (aHR 0.88, 95% CI 0.80–0.97), suggesting delayed protective associations as services matured and engagement accumulated. |
| Carnes et al., 2017, UK [76] | Mixed methods (postal survey, records analysis, and qualitative interviews) | Referred adults (frequent attenders and socially isolated patients): survey n = 184 baseline, 69 follow-up; controls n = 302 baseline, 127 follow-up, and 7,540 matched controls; qualitative n = 20 interviews | To evaluate whether a social prescribing service improved mental well-being and reduced primary care resource use. | No significant improvements were observed in general health, well-being, depression, anxiety, or active engagement at 8 months. Pre-referral GP consultation rates were high and decreased post-referral. Qualitative data indicated improvements in confidence, purpose, and motivation. Only 17% of referred patients received two or more sessions, limiting the intervention dose. |
| Cheshire et al., 2022, UK [83] | Mixed methods (nested qualitative study within a pilot evaluation) | n = 365 referrals; n = 279 attended at least one session; baseline n = 240; qualitative n = 22 service users and 7 yoga teachers | To assess the acceptability and feasibility of the Yoga4Health program as a social prescribing intervention. | Yoga4Health was highly acceptable and produced biopsychosocial benefits, including improved stress management, mood, relaxation, resilience, flexibility, balance, sleep, energy, and reduced pain. Participants also reported enhanced social connectedness, confidence, self-management skills, and healthier lifestyle behaviors. Facilitators included skilled teachers, inclusive classes, positive group dynamics, and accessible materials. Barriers included time burden, unsuitable venues, scheduling difficulties, and limited follow-up class options. |
| Chng et al., 2021, UK [123] | Qualitative (longitudinal process evaluation) | Staff from 7 GP practices in highly deprived areas (focus groups n = 31; interviews n = 14 mid-implementation, n = 19 end-of-evaluation; surveys n = 44 and 30) | To examine how the Deep End Links Worker Program was implemented in deprived-area practices and to identify facilitating and hindering factors. | Only 3 of 7 practices fully integrated the program. Fully integrated practices demonstrated shared understanding, strong team engagement, and empowered community link practitioners across all roles. Partially integrated practices showed fragmented leadership and constrained practitioner duties. Successful implementation was associated with collaborative leadership, team culture, and practitioner continuity. Turnover, poor communication, and reliance on pilot status hindered integration. |
| Christiana et al., 2025, United States [89] | Quantitative (controlled pre-post program evaluation; baseline and 3-month follow-up parent surveys) | n = 78 parent–child dyads with children in 1st–3rd grade from two rural elementary schools in North Carolina; intervention group n = 51 and control group n = 27 | To evaluate the effectiveness and feasibility of implementing the Kids in Parks TRACK Rx nature prescription program by a school nurse to increase children's time spent outdoors, nature-based physical activity, and parental intention for children to spend time outdoors | Children in the intervention group had a significant increase in outdoor physical activity on weekend/non-school days compared with the control group (p < .01). No significant changes were found for overall physical activity, weekday outdoor physical activity, time spent outdoors with family/friends/alone, or parental intention for outdoor time. Parents reported high trust in school nurses as a source of child health information, comparable to trust in primary healthcare providers. The study supports the potential role of school nurses in implementing nature prescription programs. |
| Dadswell & Bungay, 2025, UK [84] | Qualitative (exploratory descriptive study; focus groups) | n = 36 across six stakeholder groups; n = 14 Culture and Health Working Group members | To explore cultural social prescribing provision and pathways connecting people to cultural opportunities in a UK district. | Major issues were identified with unclear terminology, inconsistent understanding of social prescribing roles, and limited awareness of cultural opportunities. Barriers affecting marginalized groups included language, accessibility, transport, and cost. Facilitators included community-led outreach, inclusive communication, peer support, and co-production. A need for sustained funding, culturally competent engagement, and infrastructure to reduce inequalities in access was identified. |
| Darcy et al., 2025, England [33] | Quantitative (before-and-after cohort evaluation; baseline and follow-up questionnaires) | n = 223 community-based adults referred to green social prescribing across Humber and North Yorkshire; n = 171 had both before and after survey data. Participants were adults with mild to moderate mental health problems, with many reporting mental health conditions, disability, unemployment or inability to work, and residence in highly deprived areas. | To evaluate whether exposure to nature-based interventions through the Humber and North Yorkshire Green Social Prescribing "test and learn" program was associated with improvements in mental health and well-being, and to examine whether outcomes varied by activity type and intervention duration. | Significant improvements were reported across all ONS-4 well-being domains, and reductions in anxiety and depressive symptoms were observed, as measured by the HADS. Life satisfaction, feeling worthwhile, happiness, and anxiety improved significantly (all p < .001). HADS anxiety and depression also decreased significantly (both p < .001). Longer nature-based interventions, especially 9–12 weeks compared with 1–4 weeks, were associated with larger improvements in well-being. Horticulture and care farming activities showed stronger associations with well-being gains than some other activity types. |
| Davis, 2026, Scotland/United Kingdom [86] | Qualitative (semi-structured online interviews; reflexive thematic analysis) | n = 14 cross-sector professionals from the health, cultural, and third sectors, including health professionals, allied health professionals, community links professionals, third-sector executives, Scottish Ballet staff, and strategic stakeholders involved in or familiar with Scottish Ballet's developing dance-on-prescription pathways | To explore cross-sector professionals' perspectives on Scottish Ballet's efforts to connect dance health programs for neurological conditions into health and social care pathways through social prescribing | Professionals viewed Scottish Ballet as both a credible provider of dance-based health programs for people with neurological conditions and a strategic promoter of arts-in-health and social prescribing. Opportunities included person-centered care, improved access to dance for some patients with neurological conditions, and cross-sector advocacy. Barriers included limited professional capacity, inconsistent service-level coordination, variable professional buy-in, cultural perceptions of ballet, physical and transport access barriers, lack of sustainable funding, and insufficient system-level support. |
| Dayson et al., 2020, UK [94] | Qualitative (case study with multiple VCSE sub-cases) | n = 20: commissioners (n = 2), service providers (n = 3), VCSE providers (n = 6), patients (n = 9) | To identify emotional, psychological, and social well-being outcomes of a social prescribing model embedded in a secondary mental health recovery pathway. | Social prescribing improved emotional well-being (happiness, life satisfaction), psychological well-being (purpose, confidence, autonomy, self-acceptance), and social well-being (reduced isolation, community integration, peer support). A supportive, flexible 6-month discharge pathway enabling community engagement and peer networks was identified as a key mechanism for sustained positive outcomes. |
| Di Lorito et al., 2025, UK [51] | Qualitative (semi-structured interviews; thematic analysis) | n = 24 social prescribers supporting people with dementia across England | To identify challenges social prescribers face when supporting people with dementia and to explore strategies for addressing them. | Challenges were identified across all pathway stages, including poor referral information, communication difficulties, low motivation, cultural and language barriers, complex signposting, and client over-dependency. Effective strategies included dementia-specific training, in-person and home-visit support, personalized communication, motivational interviewing, family involvement, and improved integration between health and third-sector services. The need for dementia sensitive models and mandatory training was highlighted. |
| Dingle et al., 2024, Australia [28] | Quantitative (controlled non-randomized parallel-group evaluation) | n = 114 community-dwelling adults experiencing loneliness (SP n = 63; TAU n = 51) | To evaluate early outcomes of social prescribing compared with GP treatment as usual for reducing loneliness and improving well-being. | SP demonstrated higher retention (79.4%) and engagement in diverse social activities. Significant Time × Condition effects were observed for direct loneliness and social trust, with improvements observed only in SP participants. Small to moderate improvements in well-being, psychological distress, and social anxiety were observed in the SP group; TAU participants showed deterioration or minimal change. TAU had higher GP visits; SP had higher mental health and therapy visits. |
| Donaghy et al., 2025, UK [114] | Mixed methods (multiregional mapping study) | n = 98 SPLW stakeholders, including leads from Primary Care Networks, Health and Social Care Partnerships, and VCSE organizations | To map variation in SPLW delivery models, referral pathways, target groups, scope of support, and data recording practices across five UK regions. | Four SPLW delivery models were identified: PCN employed; grouped PCNs; VCSE commissioned or subcontracted (most common); and integrated ICB/HSCP and VCSE models. Major variation was found across referral routes, target populations, session numbers, data systems, and outcome measures. Reliance on VCSE partners, inconsistent outcome measurement, fragmented data systems, workforce pressures, and lack of standardized evaluation frameworks were identified as key challenges. |
| Donaghy et al., 2026, UK (England and Scotland) [141] | Qualitative (semi-structured interviews; reflexive inductive thematic analysis; post-hoc deductive framework analysis) | n = 130 stakeholders across eight case study sites, including 36 SPLWs, 28 patients receiving SPLW support, 28 referring professionals, 24 VCSE leads, and 14 strategic leads | To explore the roles, challenges, effects, employment models, organization, management, and sustainability of SPLWs embedded in primary care in England and Scotland. | Six themes were identified: varied SPLW backgrounds, changing role, therapeutic relationship, benefits of SPLWs, employment models and management, and challenges and sustainability. SPLWs increasingly supported patients with complex mental health, social, financial, housing, and multimorbidity-related needs. Reported benefits included patient confidence, independence, community engagement, and reduced GP moral distress, although effects on GP workload were mixed. Sustainability challenges included short-term funding, role ambiguity, burnout, retention, variable pay, limited career progression, rising demand, and pressure on statutory and voluntary-sector services. |
| Du Plooy et al., 2025, Aotearoa New Zealand [34] | Qualitative (semi-structured interviews; interpretive thematic analysis) | n = 15 former Green Prescription Otago participants; adults aged 42–77 years who had completed the program 6 months to 3 years before interview; most were referred by general practitioners, with some referred by specialists or self-referred | To explore former participants' experiences of the Green Prescription program, including perceived impacts on physical activity, mental health, quality of life, barriers and facilitators to engagement, and recommendations for program improvement | Participants reported largely positive experiences, including increased physical activity, improved mental health, enhanced quality of life, greater confidence, hope, social connection, and better management of chronic conditions. Key barriers included financial constraints, transportation difficulties, complex medical conditions, COVID-19-related disruptions, and reduced motivation after program completion. Participants valued individualized support, a strengths-based approach, subsidized activities, group-based social connection, and staff encouragement. Recommendations included extending program duration, increasing in-person support, diversifying activity options, improving program promotion, strengthening follow-up, and building stronger partnerships with community organizations. |
| Elston et al., 2019, UK [29] | Quantitative (12-month before-after evaluation with validated measures) | n = 126 adults aged 50 and older with 2 or more long-term conditions at baseline; complete 12-month follow-up n = 86 | To evaluate whether a holistic social prescribing link worker improves well-being, patient activation, and frailty, and reduces health and social care use among older adults with multimorbidity. | 85% of participants achieved their personal goals. Statistically significant improvements were observed in mental well-being (WEMWBS, +20.3%), overall well-being (Well-being Star, +43.3%), patient activation (PAM, +22.8%), and frailty (RCFS, -4.6%). Overall service use and costs increased significantly, driven by a small subset of rapidly deteriorating high-cost users (13 individuals accounted for 59% of the cost increase). No baseline characteristics predicted improvement. |
| Esfandiari et al., 2025, Canada [139] | Qualitative (online interviews; directed content analysis) | n = 11 community link workers delivering SP across nine sites serving adults aged 60 and older | To explore community connectors' experiences of implementing social prescribing, focusing on implementation factors, challenges, and future directions. | High program acceptability, feasibility, and self-efficacy were reported. Key implementation themes included variability in unmet social needs, difficulties locating community resources, role ambiguity, and the importance of communication and team relationships. Barriers included low client income, digital literacy issues, language and translation challenges, limited community assets, and inconsistent referral engagement. |
| Farina et al., 2026, Italy [47] | Qualitative (semi-structured interviews; inductive thematic analysis) | n = 15 young people aged 16–34 years participating in the Italian C.O.P.E. social prescribing pilot in the Autonomous Province of Trento. Participants were in NEET situations and varied by age, gender, educational background, duration of NEET status, social isolation, and previous contact with social, mental health, employment, or criminal justice services. | To explore young people's experiences of the C.O.P.E. social prescribing intervention and examine how it supported psychosocial readiness for labor market engagement among young people in NEET situations. | Participants described diverse and non-linear NEET trajectories shaped by social isolation, low self-confidence, mental health difficulties, fear of failure, and disorientation toward the labor market. The intervention was not experienced as a direct route into employment, but as a relational and preparatory space that supported trust-building, emotional stabilization, agency, self-esteem, clarity, and gradual re-engagement. The Link Worker was central, acting as an "in-between" figure combining emotional support with practical employment-related guidance. Reported benefits included increased confidence, self-understanding, motivation, future orientation, and readiness to consider education or employment pathways. |
| Fixsen et al., 2020, UK [18] | Mixed methods (qualitative case study within service evaluation) | n = 24: link workers, GP staff, public health managers, IT and DWP staff, adult social care staff, and service users | To explore the development of a rural social prescribing scheme using Critical Systems Thinking and to develop a relational model of stakeholder buy-in. | Relational, operational, and systemic challenges were identified, including the complexity of the link worker role, resource limitations, and GP engagement issues. A relational model emphasizing shared vision, communication, and collaboration was proposed. Emotional buy-in was identified as a key factor in sustainability. Barriers included transport, activity costs, and technical issues. |
| Fixsen et al., 2021, UK [96] | Qualitative (interviews with longitudinal follow-up) | n = 23 stakeholders from three SP schemes, including CLPs, advisors, navigators, GPs, managers, and researchers | To examine how social prescribing schemes in Scotland adapted during COVID-19 and how the pandemic reshaped delivery models and roles. | Social prescribing was highly diverse across funding, structure, and delivery models. COVID-19 caused major shifts toward remote and digital delivery, increased mental health and crisis-related referrals, and expanded SPC responsibilities. SPRING advisors shifted toward health education roles; rural Community Navigators focused on digital inclusion. SPCs faced heavier workloads, diminished third sector resources, and job insecurity. |
| Fixsen & Barrett, 2022, UK [79] | Qualitative (semi-structured interviews; inductive analysis) | n = 28 stakeholders, including GPs, link workers, advisors, navigators, managers, and researchers | To explore challenges and opportunities in delivering green social prescribing during and after COVID-19. | Stakeholders reported benefits of nature-based social prescribing for mental health, physical activity, and social connection. GPs highlighted strong benefits of schemes such as parkrun; link workers emphasized barriers, including health anxieties, mobility limitations, transport deficits, and increased distress during COVID-19. The pandemic severely reduced community service access and limited referrals, particularly for older adults, those with multimorbidity, and those in high-deprivation areas. |
| Foster et al., 2021, UK [69] | Mixed methods (pre-post quantitative; comparator analysis; interviews; SROI) | National referral sample n = 10,643; loneliness subsample n = 2,250; follow-up n = 101; qualitative n = 50 stakeholders (26 service users, 15 link workers, 9 volunteers) | To evaluate the impact of a national social prescribing program on loneliness, identify implementation facilitators and barriers, and assess economic impact through SROI. | Loneliness improved in 72.6% of users (mean UCLA change = -1.84); the proportion classified as lonely decreased from 83.9% to 47.4%. Younger users (under 50) showed greater improvement (76.2% vs 70.2%). SROI base case: £3.42 per £1 invested. Key barriers included volunteer recruitment and retention challenges, limited community resources, low signposting rates (34.7%), and transport and cost barriers. Improvements in loneliness were not consistently sustained at the 3-month follow-up. |
| Giebel et al., 2021, UK [80] | Qualitative (service evaluation interviews) | n = 387 adults referred to Community Connectors (322 with age data: 234 aged 65+, 53 aged 50 to 64, 35 aged 18 to 49); qualitative subsample n = 13 | To evaluate whether the Community Connectors service reduces loneliness by enabling adults to access community-based social activities. | Four themes were identified: falling out of society, easy self-referral, structured supportive service, and reconnecting with community. Increased confidence, stronger social networks, reduced loneliness, greater motivation, improved emotional well-being, and enhanced engagement in meaningful activities were reported. The service was valued for its accessibility and person-centered support. |
| Giebel et al., 2022, UK [125] | Quantitative (longitudinal pilot; baseline, 3 months, 6 months) | n = 14 people with dementia; n = 11 family carers | To evaluate the long-term effects of a socially prescribed community service offering physical and mental activities for people with dementia and family carers. | Significant improvements in SWEMWBS well-being scores were observed in 3 months (p = 0.000) and 6 months (p = 0.001). 59% improved by 3 months and 80% by 6 months. No correlation was found between the number of visits and the change in well-being. Discontinuation was mainly due to transport issues and other commitments. |
| Golden et al., 2023, USA [42] | Mixed methods (surveys, focus groups, and qualitative analysis) | n = 84 referred participants aged 4 to 90; n = 21 cultural organization staff; n = 33 healthcare providers | To evaluate the CultureRx arts-on-prescription pilot by examining participant experiences, provider perspectives, and challenges. | Participants reported enjoyment, feeling welcome and safe, improved well-being, and intention to return. Benefits included emotional uplift, reduced stress, increased motivation, and enhanced social connection. Providers viewed CultureRx as a valuable addition to care. Barriers included transportation, digital and technical issues, language and literacy challenges, scheduling constraints, and staff turnover. |
| Golubinski et al., 2020, Germany [65] | Quantitative (cross-sectional service-use analysis) | n = 1,734 adults using a community health advice and navigation service; 36% socially prescribed | To examine whether social prescribing (doctor referral) increases adherence to a non-clinical community service compared with self-referral. | Social prescribing significantly increased adherence (IRR 1.166 to 1.204). Distance reduced adherence; overweight or obesity was associated with increased adherence. No differences by age or gender were observed. The prescription effect was weaker for patients with psychological concerns. |
| Gordon et al., 2025, England [72] | Mixed methods (cohort pilot evaluation; social return on investment analysis) | n = 25 families recruited to the evaluation, including 26 children with neurodisability aged <16 years, 36 parents/carers, and 4 siblings; the service supported 48 families overall. Participants were identified during inpatient stays at a children's hospital in North-East England. | To pilot a hospital-initiated social prescribing service for children with neurodisability and their families, and to assess feasibility, acceptability, outcomes, and social return on investment. | Hospital in-reach social prescribing was feasible and acceptable. Link workers supported families for six months after hospital identification/discharge and helped them address non-medical needs. Unmet need decreased significantly on the Support Star (p < .001), and only 6 of 151 goals were unmet. Families reported improved access to community services, reduced isolation, stronger community connection, and greater self-efficacy. The SROI analysis estimated £2.75 of social value generated for every £1 spent. |
| Gorenberg et al., 2023, UK [52] | Qualitative (interviews within realist evaluation) | n = 28 older adults; n = 25 cultural sector staff from museums, libraries, and gardens | To explain how the cultural sector can support social prescribing for older people and to refine a program theory of tailoring cultural offers. | Nine core concepts were identified as essential for tailoring cultural sector social prescribing: immersion, buddying, cafe culture, capacity, emotional involvement, perseverance, autonomy, elitism, and virtual cultural offers. Tailored offers were described as supporting older adults' well-being through immersion, psychological holding, social connection, and self-growth. Effective delivery requires clear boundaries, strong link worker and the cultural sector collaboration, and adequate resources. |
| Hamill et al., 2026, Australia [115] | Mixed methods (pilot feasibility and acceptability study; unmet social needs screening, descriptive and regression analyses, and staff survey data) | Parents/carers of children aged 0–12 years referred to the Child Health Information Link for pediatric allied healthcare services in Sydney, Australia; 114 of 144 families agreed to participate in RISE UP, and all eligible Child Health Information Link staff completed the staff survey (n = 9) | To determine whether the Routine Identification of Unmet Social Needs to Unlock Potential (RISE UP) social prescribing model of care was needed, feasible, and acceptable within a pediatric allied healthcare intake context | Most participating families reported at least one unmet social need (74/114, 64.9%). The most common unmet needs were childcare (47.4%) and employment (45.6%), followed by bills, food, and housing. Older child age was associated with increased risk of unmet needs (adjusted RR 1.07, 95% CI 1.03–1.10), and families speaking only non-English at home had a higher risk than English-only households (adjusted RR 1.40, 95% CI 1.01–1.94). Parent/carer acceptability was high (97.2%), but staff acceptability (55.8%) and feasibility (64.3%) were lower, indicating implementation barriers within existing allied healthcare intake workflows. |
| Hanlon et al., 2021, UK [126] | Qualitative (thematic analysis of interview data) | n = 12 adults aged 34 to 64 referred to Community Links Practitioners with complex health and social problems | To explore whether Self-Determination Theory explains perceived impacts of the Links Worker social prescribing intervention. | Six participants reported moderate or major improvement; two reported slight improvement; four reported no change. Improvements were tied to satisfaction of relatedness, competence, and autonomy. Greatest improvements occurred when patients engaged in community activities, developed social connections, and shifted toward intrinsic motivation. Non-improvement occurred when psychological needs were unmet or when structural barriers, including poverty and health limitations, restricted change. |
| Hassan et al., 2023, UK [121] | Mixed methods (workshop, survey, and thematic synthesis) | n = 12 workshop participants (practitioners and Public Advisors); n = 49 survey respondents describing 50 SP programs | To explore social prescribing practices across the NIHR CLAHRC/ARC NWC region and identify essential elements for effective and sustainable implementation. | Three core elements for successful SP were identified: a personalized approach, meaningful public involvement, and whole systems working. Internal challenges included capacity constraints, high caseloads for link workers, and evaluation difficulties. External challenges included short-term funding, volunteer shortages, and insufficient stakeholder support. A visual model summarizing essential elements and contextual influences was developed. |
| Hazeldine et al., 2021, UK [122] | Qualitative (researcher-in-residence model) | n = 11 link workers; n = 2 link worker managers; n = 1 counselor | To explore early implementation of social prescribing link worker programs and identify key barriers, facilitators, and contextual factors. | Six implementation factors were identified: many referrals involved moderate to severe mental health needs beyond program remit; link workers required additional mental health training; workforce support was inconsistent, with centralized models offering stronger supervision; GP practice receptiveness and community activity accessibility shaped outcomes; SP champions facilitated integration and improved referral quality; and individual link workers developed local solutions to implementation challenges. |
| Heijnders & Meijs, 2018, Netherlands [116] | Qualitative (exploratory interviews) | n = 10 adults aged 48 to 91 referred for psychosocial problems | To explore what happens during the social prescription pathway and identify changes in social participation. | Participants trusted the GP referral, and well-being coaches functioned as connectors between primary care and community organizations. Strengths based intake and personalized activity matching increased engagement. Lack of follow-up contributed to dropout. Increased self-confidence, self-reliance, social contacts, and perceived improvements in health were reported. Mismatched activities reduced engagement. |
| Hejlesen and Jensen, 2026, Denmark [48] | Qualitative (focus group interviews and written survey responses; thematic analysis) | Young adults aged 18–30 years participating in the 10-week Culture Vitamins for Young Adults Arts on Prescription program in Denmark. The program targeted young adults with mental health challenges, including anxiety, depression, and stress, who had dropped out of education or were unemployed. Eighteen participants took part in four focus group interviews, and four additional participants completed written survey responses. | To explore the impact of an Arts on Prescription program on the mental health, psychosocial well-being, social connection, daily routines, identity development, and life transitions of young adults disengaged from education and employment. | Participants reported improved mood, reduced depression, greater confidence, reduced isolation, strengthened daily routines, increased self-understanding, and greater readiness to pursue education, employment, or other future pathways. Group-based creative activities supported peer connection, identity development beyond illness or unemployment, and a sense of belonging. Hands-on arts activities helped participants rediscover creativity and experience achievement. However, some participants experienced fatigue, stress, guilt about non-attendance, discomfort with certain activities, and concern about limited follow-up after the program ended. Findings suggest that Arts on Prescription can support well-being and transitional processes among young adults when carefully designed, safeguarded, integrated into systems, and supported by sustained follow-up. |
| Hoffmeister et al., 2024, Portugal [112] | Qualitative (participatory interviews; CFIR-guided analysis) | n = 9 stakeholders, including family doctors, link workers, social workers, and community partners | To identify enablers, barriers, and lessons learned during the implementation of a social prescribing program in Portuguese primary care. | Key enablers included proactive professionals, adaptable tools, strong intersectoral communication, visible link workers within the health unit, and structured engagement strategies. Key barriers included limited community resources, low patient awareness, limited time and funding, fragmented communication systems, long waiting times, low adherence, and lack of systematic follow-up data. |
| Hoffmeister et al., 2026, Portugal [146] | Quantitative (cross-sectional study using routinely collected referral forms and electronic health records) | n = 1,298 referrals to the first social prescribing program in Portugal, implemented in two Family Healthcare Units in downtown Lisbon between September 2018 and December 2023 | To analyze factors associated with specific reasons for referral to a social prescribing program in Lisbon and examine how referral reasons changed before, during, and after the COVID-19 pandemic | The most common reasons for referral were social and financial support (58.9%), social isolation (29.3%), unemployment (19.8%), mental health concerns (18.8%), functional dependency (16.0%), and sedentary lifestyle (13.0%). Social isolation, mental health concerns, and a sedentary lifestyle commonly co-occurred, suggesting a cluster of psychosocial and behavioral needs. Social and financial support was the leading referral reason across all periods and increased during COVID-19. Functional dependency also increased during the pandemic, while referrals for sedentary lifestyles decreased during and after COVID-19. The findings highlight the need for social prescribing services to monitor referral trends and develop community responses that can address multiple overlapping needs. |
| Holding et al., 2020, UK [107] | Qualitative (telephone interviews; interpretive analysis) | n = 24 participants (link workers n = 15; volunteers n = 9) | To explore staff and volunteer experiences of delivering a national social prescribing program to reduce loneliness and identify the required community infrastructure. | Three themes emerged: the complexity and workload burden of the link worker role; operational challenges, including inappropriate referrals, high caseloads, and limited volunteer capacity; and critical dependence on local community infrastructure. Link workers tailored the national model to local contexts, sometimes creating new community groups. Transport barriers and funding cuts constrained sustainability. |
| Holt, 2020, UK [102] | Quantitative (longitudinal repeated-measures design) | n = 66 adults referred for anxiety, depression, social isolation, chronic pain, or low mood | To measure the immediate impact of arts-on-prescription workshops on mood and examine whether mood change predicts global well-being change. | Global well-being increased significantly over the 12-week program. Immediate mood improved after every workshop across contentment, calmness, and alertness dimensions. Baseline mood on arrival improved across weeks. Reduction in tense arousal predicted long-term well-being gains. |
| Hou et al., 2026, England [73] | Quantitative (quasi-experimental study; dynamic staggered difference-in-differences analysis with propensity-matched controls) | n = 1,304 patients receiving the Central Locality Integrated Care Service (CLICS) social prescribing intervention and n = 5,216 matched controls from 25 inner-city general practices in Bradford, England; patients were matched on ethnicity, deprivation, age, gender, and health conditions | To evaluate the impact of the CLICS social prescribing intervention on unplanned hospital admissions and unplanned accident and emergency attendances in an ethnically diverse and deprived population | CLICS was associated with reduced unplanned hospital use. Intervention patients were 2.1 percentage points less likely to have an unplanned hospital admission (95% CI −3.8% to −0.4%, p = .013) and 2.4 percentage points less likely to have an unplanned accident and emergency attendance (95% CI −4.6% to −0.2%, p = .03) compared with matched controls. Effects appeared to increase over time. Subgroup analyses showed heterogeneity by ethnicity: reduced unplanned admissions were observed mainly among patients of Pakistani heritage, while reduced accident and emergency attendances were statistically significant among White British patients. |
| Howlett et al., 2025, Scotland [99] | Qualitative (interviews and descriptive costing data) | n = 28 stakeholders, including service users, link workers, activity providers, and referrers | To evaluate the implementation of Green Health Prescriptions using the APEASE criteria. | Green Health Prescriptions were perceived as acceptable and beneficial. Improvements in mental health, mood, physical activity, sleep, and social connection were reported. Facilitators included supportive link workers, activity variety, and free or low-cost access. Major barriers included a lack of integrated IT systems, poor routine data capture, inconsistent referral pathways, transport challenges, and limited accessibility for mobility-restricted users. Costs were estimated at £64 to £102 per referral. |
| Ikeda et al., 2026, Hawaiʻi, United States [54] | Mixed methods (feasibility study; recruitment, adherence, retention, exploratory well-being outcomes, and semi-structured interviews) | Firefighters from Hawaiʻi Island county fire department; 45 enrolled participants, with 24 firefighters completing four or more loko iʻa sessions and participating in pre-post assessments and interviews. Most analytic-sample participants were male, active-duty firefighters, partnered, parents, and many identified as Native Hawaiian. | To examine the feasibility of a culture-based social prescribing intervention for firefighter well-being and explore how restoring a loko iʻa, a Hawaiian fishpond, affected participants' well-being. | The intervention showed strong feasibility signals, including a 90% enrollment rate among accepted participants, 53.3% adherence to the revised four-session threshold, and strong retention among those who reached this threshold. Exploratory well-being outcomes showed small, non-significant improvements in psychological well-being and PTSD symptoms. Qualitative findings indicated high acceptability and described spiritual renewal, stress relief, mental clarity, connection to ʻāina, cultural identity, ancestral connection, purpose, camaraderie, relational well-being, and alcohol-free peer connection. Implementation challenges included communication clarity, scheduling, safety concerns, and logistical barriers. |
| Jensen, 2019, Denmark [103] | Qualitative (semi-structured interviews; salutogenic lens) | n = 7 adults referred by job center for depression, anxiety, or stress | To explore mental health well-being benefits of participating in the 10-week Culture Vitamins Arts on Prescription project. | Improved mental well-being was reported, including increased energy, reduced panic attacks, higher self-esteem, increased motivation, greater joy in life, and enhanced self-care. Participants described overcoming initial challenges, experiencing the program as a supportive free space, and shifting from self-criticism toward self-caring through cultural and artistic engagement. Participants also reported feeling closer to labor market participation. |
| Jensen et al., 2025, Sweden [101] | Mixed methods (pre-post quantitative and qualitative interviews) | n = 112 adult primary healthcare patients recruited from 18 healthcare centers; qualitative subsample n = 28 | To investigate the psychosocial effects of a 10-week group-based Arts on Prescription program. | Highly significant improvements in mental well-being (SWEMWBS) and salutogenic health (SHIS) were observed (p < 0.001). Stronger gains were associated with female sex, poorer self-rated health, more frequent healthcare contacts, and no prior arts engagement. Arts on Prescription was experienced as supportive for stress management, social connection, and reducing loneliness. Effects were greatest among vulnerable participants. |
| Jensen & Brorsson, 2025, Sweden [30] | Qualitative (exploratory interviews) | n = 28 primary care patients with stress, anxiety, depression, or loneliness | To explore psychosocial effects of a 10-week Arts on Prescription program and identify contributing processes. | Three interlinked themes described positive psychosocial change: Social Community and Connectedness (reduced isolation, strengthened belonging, and the development of friendships); Self-efficacy (greater confidence, personal growth, emotional coping, and regaining identity and purpose); and Routine and Structure (predictability, behavioral activation, and the re-establishment of daily routines). Concerns about sustaining benefits after the program ended were also reported. |
| Jensen & Bungay, 2021, Sweden [53] | Qualitative (exploratory descriptive interviews) | n = 10 primary care practitioners referring patients to Arts on Prescription | To explore Swedish primary healthcare practitioners' perspectives on referring patients with mental health issues to Arts on Prescription. | Two themes were identified: Impacts on Individuals (Arts on Prescription supported motivation, routine building, social connection, self-confidence, and self-esteem, and acted as a catalyst for activation); and Social Issues and Social Impact (Arts on Prescription provided an alternative pathway for addressing psychosocial drivers of primary care visits, potentially reducing revolving door cases). Barriers included time pressures, dominance of the medical model, and short-term project-based program structures. |
| Jonsson et al., 2026, Sweden [93] | Qualitative (theory-informed process evaluation; longitudinal and post-implementation interviews, prescriptions, and follow-up materials) | Prescribers from 10 Swedish primary care centers and 16 older adult patients aged 69–92 years who had experience with the social prescribing model. Prescribers included occupational therapists, nurses, psychologists, assistant nurses, rehabilitation coordinators, and healthcare counselors. | To unpack how and under what conditions a Swedish social prescribing model was implemented in primary care to reduce loneliness and promote health among older adults. | The social prescribing model was perceived as relevant and timely in Swedish primary care, but early implementation was limited. Four main themes were identified: the model was initially relevant in theory; it was not consistently aligned with prescribers' perceptions of loneliness; it was not compatible with everyday organizational practice; and it often did not meet patients' needs or expectations. Only 27 patients were recruited across 10 centers, far below the expected target. The findings show that social prescribing was difficult to normalize in Swedish primary care without stronger fidelity to core components, clearer organizational commitment, and better connection to community resources. |
| Kellezi et al., 2019, UK [9] | Mixed methods (interviews and longitudinal survey) | n = 630 adults referred to a social prescribing pathway (follow-up n = 178); qualitative n = 35 (7 GPs, 3 health coaches, 6 link workers, 19 patients) | To assess whether the Social Cure model explains mechanisms within a social prescribing pathway and whether psychosocial processes predict changes in primary healthcare usage. | Staff and patients recognized loneliness and social isolation as major health drivers. Group memberships increased over time and increases in community belonging combined with reductions in loneliness mediated reductions in primary care use. Social connectedness emerged as a central mechanism in the pathway. |
| Kiely et al., 2024, Ireland [77] | Mixed methods (convergent process evaluation) | n = 238 adults with multimorbidity in deprived urban GP practices; intervention n = 102; follow-up survey n = 66; qualitative n = 53 (25 patients, 11 GPs, 10 link workers, 8 community resource providers) | To examine how a general practice-based link worker social prescribing intervention was implemented during COVID-19 and to identify facilitators, barriers, and mechanisms shaping community resource connection. | Recruitment was 19 to 20% of eligible patients, hindered by complex paperwork, literacy issues, GP workload, and COVID-19 disruptions. Core components were delivered with high fidelity, but a 1-month duration was insufficient for complex multimorbidity. Only 35% of patients contacted community resources; 26% remained engaged at 1 year. Facilitators included GP endorsement, co-location, and strong link worker relational skills. |
| Kiely et al., 2024, Ireland [147] | Quantitative (pragmatic RCT with cost-utility analysis) | n = 251 adults with multimorbidity from 13 GP practices; randomized n = 240 (intervention n = 123; control n = 117); follow-up n = 207; link workers n = 10 | To examine the feasibility, health impacts, and cost-effectiveness of practice-based link workers for adults with multimorbidity in deprived areas. | Feasibility was established for practices and link workers, but patient recruitment was far below target due to COVID-19. No significant differences were found in primary outcomes (EQ-5D-5L, HADS). Per-protocol analysis showed greater activity participation among those who met with the link worker. Subgroup analysis indicated a small improvement in well-being (ICECAP-A) for men. Cost-utility sensitivity analysis suggested a high probability of cost-effectiveness at full link worker capacity. |
| Loftus et al., 2017, Northern Ireland [127] | Quantitative (pre-post quality improvement study) | n = 68 adults aged 65 and older with chronic conditions | To determine whether social prescribing activities influence GP workload and polypharmacy among older adults. | Program uptake was low (41%). No significant changes were observed in GP visits, home visits, telephone calls, repeat prescriptions, or total medications at 12 weeks or 6 to 12 months. Long delays (median 92 days) and high dropout reduced engagement. |
| Ma et al., 2025, USA [108] | Mixed methods (pre-post evaluation and qualitative interviews) | n = 15 emerging adults aged 18 to 25 who screened positive for loneliness | To evaluate the feasibility, acceptability, and preliminary impact of adapted CBT and SP interventions for loneliness in primary care. | SP was feasible and acceptable, with high completion (93%). Pre-post reductions in loneliness, depression, and anxiety were observed but were non-significant given the small sample size. Qualitative findings identified improved self-understanding, increased confidence, greater sense of control, and behavioral changes. Recruitment via electronic health records was challenging. |
| Makanjuola et al., 2022, UK [70] | Mixed methods (SROI evaluation) | n = 32 adults with anxiety, stress, PTSD, OCD, or depression (online and face-to-face clients) | To evaluate the effectiveness and social return on investment of the EmotionMind Dynamic lifestyle coaching program across face-to-face and online delivery formats. | Both delivery formats improved mental well-being and self-efficacy. SROI ratios were GBP 4.12 to 7.08 per GBP 1 for face to face and GBP 2.36 to 3.34 per GBP 1 for online delivery. Enhanced confidence, emotional regulation, coping, and reduced anxiety and stress were reported. Small reductions in NHS mental health-related service visits were also observed. |
| McGarity-Shipley et al., 2025, Canada [74] | Quantitative (service evaluation of a 10-month rural primary care pilot; health, well-being, and healthcare utilization outcomes) | Clients of a rural family health organization in Ottawa, Canada, who received support from an embedded social prescribing link worker; outcome data were available for a small subset of participants, including n = 3 with first- and third-visit well-being/loneliness measures and n = 16 with discharge healthcare utilization data | To describe a rural social prescribing pilot intervention delivered through collaboration between a community resource center and a family health organization, and to assess its impact on client health, well-being, and healthcare utilization | Among the small subset with repeated outcome data, well-being improved by 43% and loneliness improved by 23%. Among participants with discharge healthcare utilization data, hospital, emergency department, and paramedic use decreased over the program period, while family doctor visits increased. Findings suggest potential benefits of embedding social prescribing link workers in rural primary care, but results are limited by the very small number of participants with complete outcome data. |
| McGowan et al., 2026, Ireland [35] | Quantitative (retrospective observational cohort study using routinely collected service data; pre-post well-being measures) | n = 370 patients aged ≥65 years referred from a tertiary hospital geriatric medicine department to a dedicated link-worker social prescribing service in Dublin, Ireland; mean age 80.1 years; 66% female; 65% lived alone; 36% had dementia, and 20% had mild cognitive impairment | To describe the demographic and clinical characteristics of older adults referred to a hospital-based social prescribing service, reasons for referral, service engagement, types of support provided, and changes in well-being scores before and after the intervention | Social isolation and loneliness were the most common referral reasons, followed by dementia-specific cognitive activities and re-connection after illness or functional decline. Of 370 referrals, 210 completed the program. Participants received telephone support, advocacy, community referrals, and information on local activities. Among completers with paired measures, WEMWBS improved significantly from 50.2 to 54.8 (p = .006, n = 94), and WHO-5 improved from 55.5 to 68.7 (p = .017, n = 90). MYCaW well-being and concern scores improved but did not reach statistical significance. The program was feasible in an acute hospital geriatric setting, but engagement was limited by poor health, hospitalization, long-term care admission, mobility, transport, dementia, and COVID-19-related barriers. |
| McMullen et al., 2024, UK [43] | Qualitative (reflexive thematic interviews) | n = 27 Pakistani family carers; n = 10 SP stakeholders | To explore how culturally adapted social prescribing could support Pakistani carers and identify cultural, religious, linguistic, and structural influences on access. | Two overarching themes were identified: Individual and community influences (carer identity, stigma, gendered norms, language barriers, and low SP awareness hindered engagement; culturally adapted and gender sensitive activities were valued); and Societal and structural challenges (funding shortages, poor primary care and SP integration, unclear terminology, and limited cultural competence). Nearly all carers had not heard of social prescribing but expressed interest once it was explained. |
| Mercer et al., 2019, Scotland [31] | Quantitative (quasi-experimental cluster RCT) | n = 288 intervention patients referred to CLPs; n = 612 comparator patients | To assess whether referral to a community link practitioner improves quality of life, well-being, mental health, and health behaviors in high socioeconomic deprivation. | Intention-to-treat analysis found no significant effects on EQ-5D-5L, ICECAP-A, HADS-A, HADS-D, or lifestyle behaviors. In the subgroup attending three or more consultations, small but significant improvements were observed in quality of life, depression, anxiety, and exercise. A positive correlation was found between the number of sessions and the uptake of community resources. |
| Moffatt et al., 2017, UK [44] | Qualitative (interviews; thematic analysis) | n = 30 adults aged 40 to 74 with long-term conditions and common mental health issues | To explore the experiences of adults with long-term conditions referred to a Link Worker social prescribing program (Ways to Wellness). | Increased self-confidence, sense of control, physical activity, healthier eating, and improved long term condition management were reported. Reduced social isolation, better mental health, resilience, and socioeconomic improvements, including debt and welfare support, were also described. A trusting Link Worker relationship and long term, personalized, holistic support were identified as central to outcomes. |
| Morimoto et al., 2023, Japan [91] | Qualitative (semi-structured interviews; SCAT analysis) | n = 10 adults involved in a community library program, including unemployed workers, carers, students, and volunteers | To identify the functions of a community library within a social prescribing initiative and examine its impacts on individuals and the broader community. | The community library functioned as a multifunctional social prescribing site, offering a safe space, accessible consultation, social support, and cross-generational connection. Reduced loneliness, increased trust, improved communication and digital skills, a sense of belonging, and participation in co-created activities were reported. Community-level impacts included expanded social networks, civic participation, mutual aid, and social inclusiveness. |
| Morris et al., 2022, UK [78] | Qualitative (remote interviews) | n = 5 link workers; n = 8 managers; n = 44 adult clients with 12 months or more of SP engagement | To explore how a social prescribing service adapted during the first wave of COVID-19 and how clients experienced these changes. | The service rapidly shifted to remote delivery, improving access for some clients while creating challenges in rapport building, digital exclusion, and continuity of support. Client experiences varied: some received consistent, holistic support, including bereavement assistance; others experienced sporadic or no contact. Disruption to voluntary and community sector services led to setbacks in physical and mental health management. |
| Morris et al., 2025, UK [95] | Mixed methods (ED record review and staff survey) | n = 1,057 ED patients supported by SPLWs; n = 43 ED staff | To evaluate the feasibility, reach, and acceptability of an Emergency Department based social prescribing pilot. | The ED-SP model reached a large, diverse cohort with complex social, emotional, and practical needs. Mental well-being, housing, social isolation, financial insecurity, and substance use were common referral reasons. SPLWs signposted or referred 79% of patients to 204 community organizations. ED staff reported reduced workload and reduced moral injury. Barriers included limited staff awareness of referral pathways out of hours and incomplete routine data. |
| Muhl et al., 2025, Canada [49] | Qualitative (descriptive program evaluation; semi-structured interviews and deductive thematic analysis) | n = 33 children and youth aged 0–17 years participating in a social prescribing program and n = 30 caregivers from families served by a pediatric integrated health and social service hub in an underserved inner-city neighborhood in Ottawa, Canada | To explore how a child and youth social prescribing program may foster self-determination through the Circle of Courage framework, including independence, belonging, mastery, and generosity | The program appeared to foster self-determination by supporting children's decision-making, autonomy, trusting relationships, belonging, confidence, skill acquisition, and sense of purpose. Children co-produced social prescriptions with connectors based on their needs, strengths, and interests. Families reported increased independence, social connection, community belonging, new skills, confidence, and opportunities for prosocial contribution. |
| Muhl et al., 2026, Canada [62] | Qualitative (descriptive program evaluation; semi-structured interviews and inductive thematic analysis) | n = 68 participants, including 33 children and youth enrolled in the social prescribing program, 30 caregivers, and five staff members at a pediatric integrated health and social service hub in Ottawa, Canada. Program participants were aged 4–17 years and came from families facing socioeconomic disadvantage, disability, newcomer status, racial/ethnic minority status, low income, and single-caregiver household circumstances. | To understand the successes and challenges of a child and youth social prescribing program from the perspectives of program participants, caregivers, and staff members. | Successes included high participant and caregiver satisfaction, positive relationships with connectors, improved social connectedness, increased community engagement, reduced financial and transportation barriers, provider satisfaction, and stronger community cohesion. Children and youth were supported in leaving the house, trying new activities, making friends, and feeling more connected to their community. Challenges included finding the right social prescription, overcoming health, caregiver, financial, and logistical barriers to engagement, and managing operational constraints, including staff turnover, limited resources, and difficulties coordinating with community organizations. |
| O'Connor et al., 2024, Australia [55] | Mixed methods (hybrid effectiveness-implementation feasibility study; routinely collected data, outcome measures, artist notes, and stakeholder interviews) | n = 4 dyads who completed Arts on Prescription at Home, consisting of community-dwelling people living with dementia and their family carers in Sydney, Australia. Participants with dementia were aged 81–90 years; most carers were daughters or spouses living with the person with dementia. | To evaluate the feasibility of measuring outcomes from Arts on Prescription at Home for people living with dementia and family carers, and to assess implementation of the model within an existing community aged care service. | All participants with dementia reported improvements in overall health and well-being after the program. Carer well-being scores improved from baseline to post-program, and all participants reported achieving their goals. The program was feasible, acceptable, and delivered with good fidelity using existing Australian government community aged care funding. Implementation challenges included COVID-19 disruptions, artist availability, geographic reach, referral-to-program conversion, and matching art modality to client preference. |
| Ogrin et al., 2026, Australia [100] | Mixed methods (community case study; process and engagement measures, qualitative impact stories, and implementation learning) | Connect Local targeted older adults in metropolitan Melbourne with chronic conditions who were experiencing or at risk of loneliness, social isolation, and/or depressive symptoms. Spark targeted midlife women in Adelaide through a community-led social connection initiative delivered via a neighborhood community center and trained volunteers. | To examine how evaluation of social prescribing and social connection initiatives can be conceptualized and conducted within complex adaptive systems, using the evaluation journeys of Connect Local and Spark as case studies. | The study showed that conventional linear evaluation models were insufficient for capturing the relational, adaptive, and system-level impacts of community social prescribing. Connect Local and Spark both evolved during implementation, requiring flexible evaluation approaches. Key learning included the importance of co-design, community voice, relational indicators, adaptive monitoring, social capital, participant-defined goals, and recognition of system-level ripple effects such as new partnerships, changes in local provider practice, and community capacity-building. |
| Oliveira et al., 2026, Portugal [68] | Qualitative (focus groups; reflexive thematic analysis following a non-randomized pilot cohort intervention) | n = 18 people living with type 2 diabetes mellitus who completed the 12-week REDE D+ social prescribing program in a primary healthcare context in the Lisbon metropolitan area; mean age 68.4 years; 72.2% female; most were retired and had lived with diabetes for ≥6 years | To assess the acceptability, satisfaction, perceived benefits, barriers, facilitators, and implementation experiences of the REDE D+ social prescribing program for self-care and health literacy among people with type 2 diabetes mellitus | Participants reported high acceptability and satisfaction with the program. Perceived benefits included improved diabetes knowledge, health literacy, awareness of complications, self-care behaviors, physical activity, diet, medication management, blood glucose and foot self-monitoring, quality of life, social interaction, and reduced loneliness. Key facilitators included peer support, group activities, the trusted nurse social prescriber, community stakeholder support, SMS reminders, pedometers, and the SP passport. Barriers included age-related physical limitations, osteoarticular pain, initial difficulty using the SP passport, low self-esteem, preconceived ideas, and challenges maintaining commitment. |
| Opacin et al., 2026, Australia [63] | Mixed methods (single-arm pre-post study; quantitative measures, ethnographic observation, participatory action research, photovoice, interviews, focus groups, and WhatsApp conversations) | n = 37 LGBTIQA+ asylum seekers and refugees aged 21–52 years in Melbourne, Australia, from 15 countries across Africa, the Middle East, Asia, South America, and Eastern Europe. Participants represented diverse cultural backgrounds, sexualities, gender identities, migration histories, languages, and settlement experiences. | To examine how the co-designed Friends in Nature nature-based social prescribing intervention shaped loneliness, social connection, quality of life, nature connectedness, and experiences of belonging among LGBTIQA+ asylum seekers and refugees. | Participation in Friends in Nature was associated with reduced loneliness and increased nature connectedness, while health-related quality of life showed no statistically significant change. The full analysis showed a significant reduction in global loneliness and emotional loneliness. Qualitative findings identified five themes: improved mental well-being, stronger social connection and belonging, cultural sensitivity and confidence-building, the relationship between nature, memory, and safety, and the vital role of facilitators. Nature was experienced as an active agent supporting safety, recognition, shared meaning, calm, cultural memory, and hope among participants facing displacement, marginalization, and settlement insecurity. |
| Papon et al., 2026, Spain [75] | Mixed-methods economic evaluation combining a quantitative social return on investment analysis alongside a randomized controlled trial with qualitative validation data (focus groups, interviews, and facilitator learning diaries) | n = 320 adults aged ≥18 years experiencing loneliness and living in socioeconomically deprived areas of Barcelona, randomized 1:1 to the Friends in Nature intervention or usual care; SROI analysis included participants with available 3-month outcome data | To quantify the social return on investment of the Friends in Nature nature-based social prescribing intervention compared with usual care for alleviating loneliness | The Friends in Nature intervention generated EUR 1.48 in social value for every EUR 1 invested. Net present value was EUR 8,585 per Friends in Nature participant and EUR 8,496 per usual care participant. SROI ratios remained above one in most sensitivity analyses, ranging from EUR 1.31 to EUR 13.18 per EUR 1 invested. Qualitative validation supported key outcomes, including reduced loneliness, improved connectedness, increased sense of belonging, better well-being, confidence to engage in new hobbies and activities, and a stronger connection with local natural environments. |
| Payne et al., 2020, UK [45] | Qualitative (phenomenological interviews and synthesis) | n = 17 adults aged 45 to 84 | To explore how social prescribing benefits individuals participating in socially prescribed activity. | A five-step sequential benefit process was identified: receiving personalized support for social problems; engaging with others through socially prescribed activity; learning new skills and ways of relating; recognizing personal assets and becoming open to new futures; and developing hope and a positive outlook. Benefits encompassed confidence building, reduced isolation, skill development, improved well-being, and growing independence. |
| Pescheny et al., 2018, UK [120] | Qualitative (face to face interviews) | n = 10 service users; n = 3 GPs; n = 2 navigators in Luton SP program | To explore factors influencing uptake and adherence to a social prescribing program from service user, GP, and navigator perspectives. | Uptake was promoted by trust in GPs, perceived benefits, the navigator's initial contact, and availability of free sessions. Barriers included stigma around psychosocial problems and patient expectations of medical solutions. Adherence was supported by the navigator's person-centered approach, ongoing practical and emotional support, and choice in onward referrals. Lack of provider follow-up and limited free session availability hindered adherence. |
| Pescheny et al., 2019, UK [137] | Quantitative (uncontrolled before-after Bayesian modeling) | n = 448 referred users; baseline n = 186; complete-case n = 146 | To assess changes in energy expenditure following participation in the Luton social prescribing program and examine effects of age, gender, and working status. | Energy expenditure increased across all activity levels post-intervention: walking (+41.7%), moderate activity (+5.0%), vigorous activity (+107.3%), and total physical activity (+56.3%). The probability of engaging in physical activity also increased, particularly among previously inactive individuals. Age showed a consistently negative association with MET levels; working status was negatively associated with most activity levels. |
| Pola-Garcia et al., 2024, Spain [90] | Quantitative (cross-sectional electronic health records study) | n = 1,482 SP health records; n = 428 adults | To analyze sex differences in the use, attendance, satisfaction, and perceived health improvement of a formal Social Prescription Protocol in Primary Care. | SP was used more often for females (74.8%). Significant sex differences were found in areas prescribed: females received more SP focused on physical, emotional, and relational skills. Males showed higher non-attendance. Overall satisfaction was high and slightly higher in males. Emotional and relational skills, good prior self-care, high satisfaction, and rural residence predicted greater improvement, particularly among older rural females. |
| Pollard et al., 2023, UK [56] | Qualitative (ethnographic process evaluation) | n = 20 link workers; n = 19 clients with type 2 diabetes | To explore how a social prescribing intervention was delivered by link workers and experienced by referred clients. | Major implementation challenges emerged, including weak integration with GP practices and the voluntary and community sector, high link worker turnover, and time pressure to complete assessments. Findings suggested that the lighter touch model was more beneficial for participants with fewer social disadvantages than for those with greater social disadvantages. A small reduction in HbA1c (-0.10 percentage points) was observed among people with type 2 diabetes. |
| Pollard et al., 2025, UK [59] | Qualitative (ethnographic multi-year fieldwork) | n = 19 SP clients aged 40 to 74; n = 20 link workers; n = 12 walking and gardening leaders | To examine how green social prescribing referrals were implemented within a broader social prescribing intervention and compare these pathways with gym referrals. | Gym referral pathways were straightforward but sometimes led to shame and stigma. Green social prescribing offered therapeutic experiences for some clients but required intensive coordination with link workers and skilled group leadership. Green groups were fragile due to volunteer reliance, seasonal instability, limited funding, and inconsistent group cohesion. High link worker turnover and an unstable community landscape limited green referrals. |
| Polley et al., 2026, England [50] | Qualitative evaluation using semi-structured online interviews and inductive thematic analysis | n = 9 participants, including five Armed Forces Community service users and four social prescribing link workers. Service users varied by age, Armed Forces branch, time since leaving service, and referral reason. The wider Armed Forces Community Social Prescribing Service accepted 293 people, aged 16–84 years, mostly male and mainly from Army, Royal Navy/Royal Marines, and Royal Air Force backgrounds. | To explore the experiences of Armed Forces Community service users and social prescribing link workers involved in a bespoke Armed Forces Community Social Prescribing Service in Cornwall. | Service users presented with complex and interrelated needs, including mental health difficulties, PTSD-related issues, social isolation, housing, finances, physical health, domestic abuse, food insecurity, heating problems, and difficulties navigating civilian systems. Having social prescribing link workers who were themselves veterans was central to building trust, reducing the need to explain military culture, and enabling engagement. Service users reported improved living conditions, better access to practical support, improved mental health and well-being, increased social connection, and renewed hope and optimism. |
| Porter et al., 2023, UK [92] | Mixed methods (survey and interviews) | n = 93 members of a men's community organization; n = 21 interviewed participants | To explore how social prescribing referrals impact existing Men's Shed members and describe the processes involved in joining voluntary and community organizations. | Joining Sheds typically followed major life transitions such as retirement, bereavement, or illness. Sheds provided identity, purpose, humor, skill sharing, and peer support. Social prescribing referrals were beneficial when aligned with the Shed ethos and organizational capacity, but caused harm when inappropriate, creating safety risks and threatening the informal volunteer-led culture. |
| Potter et al., 2025, UK (England) [142] | Qualitative (case study within a wider mixed-methods evaluation; RE-AIM framework; reflexive discussion group and semi-structured interviews; framework analysis) | n = 32 stakeholders involved in Community Connect, including 12 service users, 6 social prescribers, 6 voluntary service provider organizations, 6 volunteer telephone befrienders, and 2 service commissioners | To examine how COVID-19 affected the implementation, maintenance, and effectiveness of Community Connect, a primary care-based social prescribing program in the London Borough of Bexley. | Community Connect adapted to remote delivery following pandemic-related disruption. Telephone befriending was reported to reduce social isolation, increase self-worth, and improve mental well-being for some service users. Remote delivery limited support for digitally excluded users and those with complex health, mental health, housing, or financial needs. Social prescribers faced challenges in building relationships remotely, managing increased referral complexity, and maintaining consistent contact. Reduced voluntary-sector capacity, service disruption, workforce pressures, and escalating post-pandemic demand highlighted the vulnerability of social prescribing when linked community services are disrupted. |
| Redmond et al., 2019, UK [85] | Qualitative (longitudinal secondary thematic analysis) | n = 1,272 adults referred from GP practices | To explore participant experiences of the Artlift arts-on-prescription program and understand perceived therapeutic benefits and contributions to self-management. | Five themes were identified: Being with others (companionship, belonging, mutual support); Being on my own (escape and change of scenery); Doing something for me (creativity, learning, self-esteem); Losing oneself (mental respite from pain and depressive rumination); and Threshold (catharsis and personal growth). The program supported self-management of physical and mental health, reduced isolation, and functioned as a form of therapeutic experience. |
| Schoultz et al., 2026, United Kingdom [82] | Sequential mixed-methods study using quantitative pre-post well-being assessment and qualitative semi-structured interviews | Quantitative component: n = 259 Armed Forces Community service users who completed the Short Warwick-Edinburgh Mental Wellbeing Scale at initial and final consultations with Armed Forces Social Prescriber Link Workers. Qualitative component: n = 11 stakeholders, including six Armed Forces Social Prescriber Link Workers, four line managers, and one general practitioner. | To evaluate the impact of a novel social prescribing service designed specifically for the Armed Forces Community and assess its influence on service users' well-being, while exploring stakeholder experiences of service delivery and implementation. | Well-being scores increased significantly from initial consultation (mean = 15.03, SD = 3.66) to final consultation (mean = 18.79, SD = 4.17), with a mean difference of 3.76 and a 25.01% increase in well-being (t(258) = 30.7, p < .001). Despite improvement, scores remained below national averages, reflecting complex health and social needs. Qualitative findings identified four themes: Armed Forces experience and perspective; challenges and barriers; service delivery and effectiveness; and skill development and attributes. Advocacy, navigation, cultural competence, trust-building, and relationship-building were central mechanisms of the Armed Forces Social Prescriber Link Worker role. |
| Skinner et al., 2023, UK [32] | Quantitative (Social Return on Investment evaluation) | n = 54 adults with prediabetes and BMI of 30 or higher at baseline; eight-week follow-up n = 24 | To evaluate the social value of the MY LIFE program by comparing diabetes technician support alone with diabetes technician plus community-based social prescribing. | Both groups showed improvements in mental well-being (SWEMWBS) and overall health (EQ5D-5L). SROI ratios ranged from £4.23 to £5.07 per £1 for DT only and £4.67 to £5.07 for DT plus SP. Both groups reported reduced GP and nurse visits. SP activity uptake was limited due to COVID-19 restrictions. |
| Skivington et al., 2018, Scotland, UK [117] | Qualitative (framework analysis interviews) | n = 30 community organization representatives; n = 6 Community Links Practitioners | To investigate the benefits and challenges of implementing a primary care-based social prescribing program aimed at improving inter-sectoral working. | The Links Worker Program improved collaborative case management and inter-sectoral working, with community link practitioners acting as bridges between patients, GP practices, and community organizations. Benefits were driven primarily by individual relationships rather than organizational partnerships. Major challenges included austerity-driven funding cuts, reduced capacity of community organizations, and the time-intensive nature of case management. |
| Stickley & Eades, 2013, UK [104] | Qualitative (follow-up interviews) | n = 16 Arts on Prescription participants who were current or former mental health service users (n = 10 at follow-up) | To evaluate longer-term outcomes two years after initial interviews, identifying sustained hard and soft outcomes of Arts on Prescription participation. | Arts on Prescription acted as a catalyst for long term change. Soft outcomes included increased self-confidence, self-esteem, motivation, communication skills, social integration, and new identity formation (e.g., becoming an artist, poet, or carer). Hard outcomes included enrolling in art-related education (BTec, Foundation Art, BA), leading user-led arts groups, and engaging in voluntary work. Four thematic domains summarized long-term change: educational achievements, broadened horizons, identity shifts, and social and relational transformations. |
| Stickley & Hui, 2012, UK [105] | Qualitative (in-depth interviews) | n = 10 referrers (GPs, occupational therapists, social workers, voluntary sector staff) | To explore referrers' views on the quality, effectiveness, and role of an Arts on Prescription program within wider mental health and community support systems. | Referrers consistently viewed Arts on Prescription as valuable, therapeutic, and psychologically safe. Clients were reported to gain confidence, self-esteem, motivation, meaningful occupation, artistic and social skill development, and a sense of purpose. Social benefits included increased social contact, belonging, and peer support. The program was viewed as complementary to statutory mental health provision, providing a non-stigmatizing option for clients not yet ready for employment or education. |
| Sumner et al., 2020, UK [128] | Quantitative (observational cohort study) | n = 1,297 adults referred to Artlift Arts on Prescription | To identify sociodemographic and baseline factors associated with attendance, engagement, and well-being change in a large Arts on Prescription intervention. | Baseline well-being was the strongest predictor of all outcomes. Higher baseline well-being was associated with increased attendance and engagement; lower baseline well-being predicted greater improvement. Participants from the median IMD quintile were more likely to attend. Approximately 23.6% showed no improvement or declined in well-being. |
| Sumner et al., 2021, UK [129] | Quantitative (longitudinal pre-post study) | n = 245 primary care patients referred to Arts on Prescription | To assess changes in anxiety, depression, and well-being following Arts on Prescription participation across initial and re-referral cycles, including among participants with multimorbidity. | Anxiety, depression, and well-being improved significantly after the initial cycle and again after re-referral. Improvements were consistent in both the full sample and the multimorbid subgroup. Baseline outcome levels predicted change; demographic and clinical characteristics did not. Symptoms tended to rebound between cycles. |
| Taylor et al., 2026, United Kingdom [106] | Qualitative study using semi-structured interviews and reflexive thematic analysis | n = 25 participants, including young people who attended Safety Nets (n = 6), parents/carers (n = 6), clinicians (n = 9), and sports leaders (n = 5) across five sites in Yorkshire and Humber. Young people were aged 11–16 years and were on NHS CAMHS waiting lists for anxiety, low mood, and/or depression. | To explore the experiences of young people, parents/carers, and delivery staff who participated in or delivered Safety Nets, and to inform future implementation and evaluation of the intervention. | Safety Nets was well received by young people, parents/carers, clinicians, and sports leaders. Participants valued the combination of psychoeducation and physical activity, the informal and friendly atmosphere, and delivery in non-clinical professional sports club settings. Reported benefits included improved confidence, mood, self-esteem, peer relationships, sense of belonging, anxiety management, sleep-related skills, and greater comfort in social and school settings. Staff reported professional development benefits, especially sports leaders gaining mental health knowledge. Participants recommended maintaining the relaxed atmosphere, considering a longer program duration, supporting transport access, and exploring adaptations for autistic young people. |
| Thompson et al., 2023, UK [98] | Qualitative (longitudinal interviews) | n = 26 service users enrolled in Community Connectors; n = 12 at follow-up | To explore service users' experiences of receiving support through a national social prescribing scheme addressing loneliness and social isolation. | Service users highly valued the one-to-one relationship with the link worker or volunteer, often prioritizing this over signposting to community activities. The program first built confidence for daily tasks before enabling community resource engagement. The short-term nature of support made endings emotionally challenging; several participants reported deterioration in mood after support ended. Sustainability was hindered by weak community infrastructure, mobility barriers, and inadequate transport. |
| Thompson et al., 2026, Australia [37] | Quantitative pre-post feasibility and acceptability intervention study conducted within a concurrent mixed-methods doctoral project | n = 46 community-dwelling older people recruited from seven general practices in the Hawkesbury region of New South Wales, Australia; 40 completed the study, and 37 had paired pre-post data for analysis. Participants were mainly women, with a mean age of 82.0 years, and were at risk of loneliness and/or social isolation. | To investigate the feasibility, acceptability, and impact of a general practice nurse-led social prescribing intervention to improve social connections for community-dwelling older people. | No statistically significant changes were found in loneliness, self-rated health, or EQ-5D-5L quality-of-life dimensions at 12-week follow-up. However, 45.9% of participants reported improved self-rated health, and 29.7% reported improvement in pain/discomfort. Participants reported increased awareness of community activities, improved social interaction, greater willingness to try new activities, and high satisfaction with the intervention. The intervention was feasible and acceptable, but reach, recruitment, and activity uptake were limited by COVID-19, natural hazard events, transport barriers, and disruption to community activities. |
| Thomson et al., 2018, UK [8] | Quantitative (within-participants repeated-measures) | n = 115 older adults aged 65 to 94 with mild to moderate dementia | To assess psychological well-being changes among older adults in a 10-week museum-based social prescribing intervention. | Significant improvements were observed in all six psychological well-being emotions (absorbed, active, cheerful, encouraged, enlightened, inspired) at the start, midpoint, and end of the program. Cheerful consistently showed the highest scores; active showed the lowest. Absorbed and enlightened showed disproportionately large gains. Social bonding, object handling, and creative activities were valued by participants. |
| Tierney et al., 2022, UK [88] | Quantitative (cross-sectional online survey) | n = 148 link workers | To explore link workers' experiences of using the cultural sector within social prescribing for older adults during COVID-19. | Link workers recognized potential well-being benefits of cultural venues but rarely referred older adults to them, particularly during COVID-19. Referrals to libraries and museums declined sharply during lockdowns; gardens remained more accessible. Personal familiarity with venues strongly predicted referral likelihood. Cultural venues were frequently perceived as elitist or costly. Additional barriers included physical accessibility issues, digital exclusion, and limited communication with cultural organizations. |
| Tierney et al., 2024, UK [46] | Mixed methods (realist evaluation) | n = 7 link workers; n = 61 patients (41 follow-up); n = 93 professionals (VCSE staff, healthcare staff, link workers) | To explore implementation of link workers in primary care and determine what works, for whom, and why, with a focus on micro-discretions and role boundaries. | Link workers routinely exercised micro-discretions, using personal judgment to tailor support. These micro-discretions fostered rapport, person-centered care, and job satisfaction. When boundaries were unclear, link workers experienced role ambiguity, emotional burden, and safety risks. Employment model, supervision, training, and role clarity strongly shaped the use and consequences of micro-discretion. |
| Tierney et al., 2024, UK [118] | Mixed methods (realist evaluation) | n = 61 patients (41 follow-up); n = 35 observed in link worker sessions | To understand how patient buy-in to social prescribing and the link worker role is established. | Patients buy-in formed through two interconnected processes: connection with the link worker (trust, hope, emotional safety) and connection to external support (when readiness allowed). Engagement was shaped by link workers' interpersonal skills, empathy, reliability, and knowledge of local resources. Many patients reported increased confidence, reduced stress, and reduced reliance on GP services. Structural barriers, including transport difficulties, housing instability, and limited local resources, restricted engagement in external activities. |
| Tierney et al., 2024, England [81] | Realist evaluation using focused ethnographies, interviews, observations, documents, patient questionnaires, daily link worker debriefs, and follow-up interviews | Seven social prescribing link workers across different areas of England; n = 61 patients and n = 93 professionals from primary care and the voluntary, community, and social enterprise sector; follow-up interviews with 41 patients, seven link workers, and one link worker manager | To understand and explain how the social prescribing link worker role is implemented and integrated within primary care in England | The study developed a program theory describing a continuum of link worker integration into primary care: "bolted on," "fitting in," and "belonging." Four key concepts shaped integration: centralizing or diffusing power, forging an identity in general practice, demonstrating effect, and building a facilitative infrastructure. Successful implementation required whole-system support, clear role understanding, legitimacy within primary care, appropriate supervision, feedback loops, time to build VCSE connections, and infrastructure that allowed link workers to deliver person-centered care. |
| White et al., 2022, UK [109] | Mixed methods (evaluation with a qualitative focus) | n = 57: SP managers, link workers, GPs, social workers, clients, and VCS practitioners | To examine the effectiveness of a city-based social prescribing service from multiple stakeholder perspectives. | GP referrals were extremely low (4.2%), while self-referrals (28%) and social worker referrals (20%) were considerably higher. Link workers provided skilled support, including exploring complex needs, building rapport, and accompanying clients to initial sessions. Barriers included GP skepticism, dominance of the medical model, limited voluntary and community sector capacity for complex mental health needs, and tension between promoting independence and avoiding dependency. |
| Whitelaw et al., 2017, UK [60] | Qualitative (case study) | n = 30 informants, including steering group members, primary care staff, and community providers | To evaluate the early implementation of the Healthy Connections Stewartry social prescribing initiative, focusing on implementation processes and sustainability within primary care. | Early implementation was reported as broadly successful, with normalization within primary care routines beginning to occur. Success was attributed to inclusive stakeholder engagement, strong public health leadership, shared commitment to holistic care, use of an IT-based referral system (SCI Gateway), and multi-sector collaboration. Key barriers included unstable funding for community resources, concerns about quality assurance, limited feedback to referrers, and uneven community capacity. |
| Whiteley et al., 2025, UK [71] | Mixed methods (SROI evaluation) | n = 20 adults enrolled; n = 19 at baseline; n = 15 at follow-up | To pilot a health economics informed SROI evaluation of the nine-week Making Well nature-based social prescribing program. | Improvements were observed in overall well-being (ICECAP-A), mental health (SWEMWBS), social connection (SWEMWBS Q6), and confidence and self-efficacy (GSES). GP appointments decreased by 40%. SROI was estimated at GBP 3.30 to 4.70 per GBP 1 invested; a forecast indicated GBP 5.40 to 7.70 per GBP 1. Increased belonging, reduced anxiety, new skills, and personal growth were reported. |
| Wildman et al., 2019, UK [110] | Qualitative (follow-up interviews) | n = 24 adults aged 40 to 74 with long-term conditions | To explore service users' experiences of link worker social prescribing, one to two years after initial engagement. | Reduced social isolation, improved condition management, better mental well-being, increased confidence, and positive changes in health behaviors (diet, physical activity, smoking cessation) were reported. The continuous link worker relationship was central to progress. Many participants experienced setbacks due to multimorbidity, mental health problems, and cultural barriers, requiring continued support. Link worker turnover and lack of suitable community services were identified as major barriers. |
| Wildman & Wildman, 2021, UK [66] | Quantitative (observational cohort study; Difference-in-Differences analysis) | n = 8,086 adults aged 40 to 74 with type 2 diabetes (intervention n = 4,752; controls n = 3,334) | To determine whether a community health worker social prescribing program was associated with improved HbA1c levels among patients with type 2 diabetes. | The program was associated with modest but statistically significant reductions in HbA1c, -0.10 percentage points overall and -0.14 after three years. Stronger associations were found for White patients, those with fewer comorbidities, those without obesity, and those in the most deprived areas. Subgroup analyses showed reductions of up to -0.19 percentage points in the most deprived deciles. |
| Wildman & Wildman, 2023, UK [97] | Quantitative (natural experiment; Difference-in-Differences analysis) | n = 8,283 adults aged 40 to 74 with type 2 diabetes (intervention practices n = 4,862; control practices n = 3,421) | To evaluate whether link worker social prescribing reduced non-elective admitted patient care use and costs. | Participation was associated with reduced non-elective inpatient care costs, with the largest reductions among highly engaged patients, up to -£77.57 per patient per year. Subgroup analyses showed greater reductions for non-White patients, older adults, and patients without additional comorbidities. Intention to treat estimates were small and non-significant; per-protocol and engagement level analyses showed meaningful reductions. |
| Woodall et al., 2018, UK [119] | Mixed methods (pre-post questionnaires and interviews) | n = 342 adults with complete pre-post well-being data; qualitative n = 26 service users; staff focus group n = 1 | To evaluate outcomes of a Well-being Coordinator-led social prescribing service and identify mechanisms supporting delivery. | Significant improvements were found in well-being (medium to large effect), self-rated health (small to medium effect), and social connectedness, alongside significant reductions in anxiety and depression. Younger users showed greater well-being gains; men showed a trend toward greater improvement. Mechanisms of success included strong, flexible Well-being Coordinator relationships, empathetic communication, and a strong voluntary and community sector infrastructure. GP utilization changes were mixed and inconclusive. |
| Zhao et al., 2026, South Korea [38] | Quantitative, quasi-experimental nonequivalent control-group pretest-posttest study using difference-in-differences analysis and structural equation modeling | n = 294 rural older adults aged ≥65 years from Wonju City, Gangwon-do, South Korea; 148 participants received the social prescribing intervention, and 146 were in the control group | To evaluate the effectiveness of a pilot social prescribing project for rural older adults and examine changes in social support, loneliness, depression, cognitive function, and life satisfaction before and after the intervention | The social prescribing intervention significantly reduced depression and improved social support and life satisfaction compared with the control group. The experimental group showed increased social support, reduced depression, and improved life satisfaction after the intervention, while cognitive function improved slightly but not significantly. Difference-in-differences analysis confirmed significant intervention effects for social support, depression, and life satisfaction. Structural equation modeling showed that social support was positively associated with cognitive function and life satisfaction and negatively associated with depression. Findings support the feasibility and mental health benefits of culturally embedded, community and digitally supported social prescribing for rural older adults. |

+
