# Supplementary material for: Models, implementation, and reported outcomes of social prescribing interventions: a scoping review
Source: Front Public Health. 2026 Jul 22;14:1871347. doi: 10.3389/fpubh.2026.1871347 (PMC13437648; doi:10.3389/fpubh.2026.1871347)
Supplement: Supplementary file 3 [file Table_2.docx]

**Supplementary Table 2. Social Prescribing Models, Reported Outcomes, Implementation Facilitators, Implementation Barriers, and Contextual Factors Across Studies**

| **Author(s), Year, Country** | **Intervention Type** | **Initiation/Coordination Setting** | **Target Population(s)** | **Outcomes Reported (Health / Social / System)** | **Implementation Facilitators** | **Implementation Barriers** | **Contextual Factors** | **Evidence Gaps / Future Priorities** |
| --- | --- | --- | --- | --- | --- | --- | --- | --- |
| Aggar et al., 2025, Australia [15] | Link worker social prescribing with co-designed individual plans and referrals addressing well-being, social needs, daily living activities, and economic issues. | Primary healthcare and community not-for-profit services in New South Wales; delivered by social workers via telephone, internet, and face-to-face contact. | Adult informal carers and adults with mental health conditions or chronic illness; self-referred or referred by general practitioners. | Measured: Improved quality of life, mental well-being, psychological distress, and self-reported health; reduced general practitioner visits; no significant change in hospital visits. Perceived: Strong engagement with well-being focused prescriptions. | Person-centered tailoring; holistic assessment; flexible referral pathways; remote delivery options | Caring burden; time constraints; lack of systematic carer identification; undifferentiated support needs; reliance on worker-collected data | Most participants were women; many had mental health needs; people stayed in the program longer than expected; carers needed more support time. | Future research should include larger and more diverse samples, develop accurate carer-identification tools, compare outcomes across caring roles, track long-term service use, include control groups, and test models that allow people to enroll in multiple services. |
| Ajibade et al., 2024, UK [39] | Link worker-led social prescribing with access to non-medical community activities (arts, sports, volunteering, gardening, cookery) and occasional direct signposting. | National Health Service general practice settings across multiple regions. | Adults with long-term conditions and psychosocial needs, including mental health difficulties, isolation, low confidence, limited support, and socioeconomic stress. | Measured: No consistent reduction in general practitioner workload; increased administrative workload. Perceived: Improved mental well-being, confidence, motivation, and self-management. | General practitioner support; link worker time and emotional support; task shifting potential; available community resources | Low awareness; stigma; preference for medical care; time-consuming referral processes; weak communication; long waits; high caseloads; worker turnover; insufficient training; inconsistent service availability | Community organizations had limited resources; services differed across regions; long-term funding was uncertain; general practitioners had heavy workloads; social prescribing was used to help with non-medical problems. | Priorities include long-term follow-up of patient outcomes, clearer evidence on cost-effectiveness and workload impact, integration of social prescribing in health education, development of standardized operational models, inclusion of link workers and public perspectives, and examination of sustainable funding structures. |
| Armitt et al., 2026, England [61] | Green social prescribing delivered through community organizations. Activities included horticulture, food growing on community allotments, home-based gardening clubs, hub-based food growing linked to English-language learning, walking groups in urban green spaces and wider countryside, and outdoor sport or physical activities such as paddleboarding and archery. | Six community-based organizations in Hull and York, England, including organizations supporting minority ethnic, migrant, refugee, and asylum-seeking communities. Activities were delivered in community allotments, green and blue spaces, community hubs, local outdoor environments, and trusted community settings. | People from ethnically diverse communities, including migrants, refugees, asylum seekers, and local minority ethnic communities. Many participants lived in highly deprived areas; 62% of participants were from the 20% most deprived postcodes in England. | Measured: ONS4 life satisfaction, feeling worthwhile, happiness, and anxiety. Anxiety improved significantly after 6 months, and most participants maintained or improved well-being across the four ONS4 domains. Perceived: improved mental well-being, relaxation, reduced stress, social connection, belonging, confidence, physical activity, connection with local green spaces, cultural and heritage connection, and family/community engagement. System: community organizations acted as trusted access points, with informal referral routes more important than healthcare referral. | Trusted community organizations; word-of-mouth referral; culturally familiar and safe group environments; community leaders collecting data and supporting participation; flexible choice of activities; family inclusion; transport and equipment support; access to green/blue spaces; food growing and sharing; links to heritage and culture; peer support; safe facilitation; community organizations acting as anchors for emotional, social, and practical needs. | Limited links with statutory and healthcare services; no participants accessed activities through healthcare referral; language barriers; challenges using ONS4 across cultures and languages; discrimination and mistrust of services; immigration insecurity; cost-of-living pressures; community unrest and riots targeting minority ethnic communities; drop-out and unplanned endings; instability due to Home Office moves or work changes; limited recruitment of people who declined or withdrew. | Conducted during a period of social and economic stress, including riots affecting ethnic minority communities in Hull and wider public hostility toward migrant and refugee communities. The study was part of England's green social prescribing test-and-learn extension program and focused specifically on underserved groups often underrepresented in green social prescribing evidence. | Future research should examine green social prescribing in other regions and rural settings, improve culturally adapted well-being measures, explore experiences of people who decline or withdraw, strengthen links between community organizations and statutory services, assess long-term outcomes, evaluate informal referral routes, and investigate how green social prescribing can reduce rather than widen health inequalities for migrant, refugee, asylum-seeking, and minority ethnic communities. |
| Aughterson, Baxter & Fancourt, 2020, UK [30] | General practitioner referral to community-based activities through a link worker (arts, gardening, peer support, walking groups). | General practice settings working with voluntary and community sector organizations. | Adults with common mental health problems, including anxiety, depression, and psychosocial distress. | Perceived: Improved motivation, sense of empowerment, confidence, social connection, and reduced isolation; increased acceptance of social prescribing among practitioners. | Link worker bridging role; trusted general practitioner relationships; personalized care; whole-practice engagement; supportive culture; collaboration; feedback processes | Limited time; inadequate knowledge and training; difficulty accessing community resources; low motivation; transport barriers; high unmet psychosocial need; workforce pressures; voluntary-sector instability; uneven practice culture | Community organizations had unstable funding; buildings and equipment were limited; pressure on the health service increased demand for support. | Evidence gaps relate to stronger demonstration of health benefits, systems for regular progress feedback to clinicians, understanding dropout and engagement patterns, research from devolved nations, safety monitoring for group activities, and assessment of community organizations' capacity. |
| Rabinowitz Bailey and DiMilia, 2026, United States [36] | Arts-based social prescribing through the Art Pharmacy model. Healthcare or community partners referred participants to Care Navigators/link workers, who matched them to personalized arts and cultural activities based on health goals, interests, and needs. Activities included workshops, classes, performances, visual arts, music, dance, movement, and cultural experiences, with approximately one arts activity per month for up to 12 months. | U.S.-based healthcare and community partner settings in California and Georgia; referrals came from healthcare, community, university, Medicaid managed care, Medicare provider groups, community mental health, cancer, and palliative care organizations. Care Navigators provided support by phone, email, or text and maintained communication with referring providers. | Adults aged ≥18 years experiencing loneliness, social isolation, depression, anxiety, or unmet mental health and social connection needs; early implementation involved participants from healthcare, behavioral health, university, and community settings. | Measured: Significant and sustained improvements in WHO-5 mental health and well-being scores over repeated follow-ups, with peak increases of approximately 18–20 points and sustained improvement through follow-up 9. Social: increased access to community-based arts and cultural activities intended to promote social connection. System: preliminary evidence for integrating arts-based social prescribing into U.S. healthcare as a complementary, non-clinical mental health support. | Personalized arts matching; Care Navigator/link worker support; motivational interviewing and behavioral activation training; mental health first aid; smart matching technology; regular follow-up after activities; transportation support; reduced or eliminated out-of-pocket costs through third-party payer arrangements; option to bring a companion; repeated well-being assessment; broad range of arts and cultural community partners. | No control group; observational design; substantial attrition across follow-up assessments; incomplete demographic data; self-reported outcomes; possible selection bias among participants with complete demographic data; potential favorability, expectancy, regression-to-the-mean, and unmeasured confounding effects; early implementation limited to California and Georgia; findings may not generalize to all U.S. SP models or populations. | Conducted in the context of increasing U.S. mental health needs, loneliness, social isolation, behavioral health workforce shortages, cost barriers, and interest in whole-person care. Art Pharmacy operates as a private, U.S.-based social prescribing organization that integrates arts and culture into healthcare and community referral systems. The model aims to reduce financial and transportation barriers and expand access to community-based arts engagement. | Future research should use controlled study designs, examine subpopulations such as youth, older adults, and college students, assess longer-term outcomes, measure attendance and participation intensity, evaluate dose-response relationships, include more diverse geographic and socioeconomic populations, examine mechanisms of change, and assess implementation, cost-effectiveness, and scalability of arts-based social prescribing in U.S. healthcare. |
| Bertotti et al., 2018, UK [57] | Holistic social prescribing with a structured general practitioner referral pathway and intensive coordinator support, including up to six sessions to co-produce personalized well-being plans and coordinate community referrals. | Primary care pathway across general practice surgeries linked with voluntary and community organizations. | Adults experiencing social isolation, mild to moderate mental health problems, psychosocial challenges, frequent primary care or emergency use, and multiple long-term conditions. | Perceived: Improved well-being, confidence, sense of purpose, self-esteem, and personal control; reduced loneliness and isolation; stronger social connections and community involvement; reported potential system value through possible reduction in inappropriate general practitioner use. | Strong coordinator-patient alliance; empathetic listening; co-location in general practices; structured communication; diverse local activities; personalized support | Low GP engagement; low awareness; short consultations; inconsistent referral practices; incomplete knowledge of services; branding issues; voluntary-sector capacity limits; short-term funding; program turnover | The area had many community organizations; commissioning bodies supported the program; high deprivation increased needs; more people were allowed to self-refer; the community sector needed steady funding to continue. | Key gaps include large-scale causal studies, clear criteria for beneficiary identification, refinement of the coordinator role, more substantial financial support for community providers, consistent communication pathways, and transparent evaluation frameworks that explain what works and why. |
| Bertotti et al., 2025, Italy & Portugal [113] | Link worker-led social prescribing with co-produced action plans, one-to-one relational support, and referral to voluntary, community, and social enterprise organizations (from general practitioners, schools, employment, and social services). | Community-based network integrating mental health, employment, education, and social care systems. | Young people aged 16-34 not in education, employment, or training with social and mental health vulnerabilities, including anxiety, depression, learning difficulties, long-term disengagement, and migrant backgrounds. | Measured: Significant increase in mental well-being score; reduced psychological distress score; reduction from clinically significant to non-clinically significant levels of anxiety and depression; stronger improvements among women, younger adults, and those with poorer baseline mental health. Perceived: Increased self-esteem, confidence, motivation, social ability, stability, emotional resilience, daily routine, social networks, and readiness for employment or education. | Trusting relationships; holistic support; flexible pacing; emotional and practical coaching; family involvement; strong third-sector collaboration; structured supervision | Retention difficulties; high time and resource demands; variation in worker skill; dependency risks; pressure for rapid employment outcomes; limited community opportunities | Funding came from a European program; the pandemic and rising living costs affected delivery; some areas had strong cooperation between organizations; each country used different ways to find participants; some partner organizations had limited capacity. | Future work should focus on long-term comparisons with other youth support models, planning for workload and cost management, advanced training for link workers, improved data systems, strategies for engaging highly disengaged youth, and policy integration into national youth services. |
| Bhatti et al., 2021, Canada [41] | Link worker and navigator-supported social prescribing with referrals to community activities (arts, diabetes support, walking groups, bereavement groups) and optional co-designed health champion and volunteering pathways. | Eleven Community Health Centers across Ontario, including urban, rural, northern, and Francophone locations. | Adults with unmet social needs, including loneliness, low income, mental health concerns, and chronic conditions. | Perceived: Improved mental well-being, stronger social support, greater sense of belonging, improved self-management of chronic conditions, increased purpose, and volunteering engagement. | Person-centered communication; autonomy-supportive approach; alignment with interests; trusted provider relationships; inclusive community-health environment | Unequal access to volunteer roles; inconsistent group availability across centers | The program was part of a community health center focused on equity; the approach was linked to ideas about autonomy, competence, and supportive relationships. | Further research should clarify which program components benefit specific groups, provide numerical outcome data, include long-term follow-up, assess cost-effectiveness, and test the model in new regions or countries. |
| Bishop-Edwards et al., 2026, England [124] | Creative social prescribing through VCSE-run community groups supported by Creative Minds. Activities included arts, sports, community and social gatherings, theatre, youth work, gardening, online meetings, postal art packs, at-home crafting, gardening activities, online games, letter writing, and other creative/social activities adapted during lockdown. | Community-based VCSE groups in South West Yorkshire, England, supported or co-funded by Creative Minds, a charity embedded within South West Yorkshire Partnership NHS Foundation Trust. During lockdown, provision shifted from regular in-person groups to online, postal, home-based, or hybrid formats. | Established members of creative/community groups, including people recovering from or living with serious mental illness, learning disabilities, neurodiversity, mental health difficulties, social marginalization, and isolation. | Health/social: participants reported connection, belonging, friendship, routine, comfort, creative identity, social confidence, peer support, emotional support, and benefits for mental health recovery. Lockdown reduced in-person connections and intensified isolation, anxiety, and marginalization. System/implementation: adapted provision maintained some contact but did not fully replace in-person groups; user and provider experiences of lockdown delivery differed. | Compassionate and skilled group leadership; warmth and interpersonal skill; leaders knowing members as individuals; peer support; established pre-lockdown relationships; creative and meaningful activities; buddy systems for new members; check-ins; one-to-one calls or visits; postal activity packs; online sessions; opportunities for shared activity, creativity, and community engagement; safe and accepting group environments. | COVID-19 lockdown restrictions; loss of face-to-face contact; digital exclusion or low engagement with online tools; anxiety linked to masks and infection risk; reduced autonomy; disruption of routine; loss of confidence to return; bereavement; age and health concerns; transport and access difficulties; stigma; social isolation; marginalized groups feeling overlooked by public health rules; online groups perceived as less collective than in-person groups. | The study used the lockdown as a natural experiment to understand which group valued creative social prescribing most. It was conducted in the context of pandemic restrictions, remote and hybrid mental health service delivery, and reliance on VCSE organizations to support marginalized groups. Creative Minds provided a community-linked model embedded within an NHS mental health trust. | Future research should examine long-term impacts of creative social prescribing from participants' perspectives, include people who stopped attending or could not access groups, replicate the study in other regions and countries, evaluate hybrid and flexible delivery models, assess how VCSE groups contribute to recovery and community resilience, and develop policies that better account for marginalized groups during public health emergencies. |
| Bos et al., 2025, Netherlands [58] | Existing model: short-term "Well-being on Prescription" with link worker support. Proposed model: long-term tailored social prescribing using a single case manager coordinating cross-sector support for wider determinants of health. | Primary healthcare centers collaborating with social, community, and voluntary sector professionals in a vulnerable urban neighborhood. | Adults with multiple physical, psychosocial, social, financial, and housing challenges in a multicultural community. | Perceived: Current model viewed as insufficient for complex needs; unmet health and social needs; frustration with fragmented care. Potential outcomes of the redesigned model include improved coordination, trust, engagement, and holistic well-being. | Stakeholder willingness to collaborate; commitment to wider health needs; value of lived experience; relational trust; culturally sensitive, flexible support | Fragmented structures; unclear roles; separate financing systems; staff turnover; time and resource limits; stigma; cultural misalignment; weak system support; low awareness of available options | The program took place in a very disadvantaged neighborhood with high poverty and unemployment; most residents had a migrant background; the existing model did not match the needs of people with complex problems; rules made it hard for sectors to work together. | Priority areas include evaluating the combined social-prescribing/case-manager model, studying cross-sector teamwork, developing culturally adapted versions, reaching individuals who avoid traditional services, and testing referral routes beyond general practice. |
| Brandborg et al., 2021, Denmark [64] | Signposting to local community physical activity programs, with optional link-worker referral; community-based non-medical physical activity support. | General practitioner consultations in primary care with collaboration from community and voluntary sports organizations. | Adults with chronic illness, mental health problems, frailty, dementia, and non-Western ethnic minority backgrounds. | Perceived: Increased motivation to begin physical activity, improved adherence to an active lifestyle, increased well-being, confidence, and self-efficacy; possible reduction in medication needs. | Sense of belonging; link worker as stable contact; accessible digital signposting; cross-sector partnerships; community-based activity options; financial support availability | Expectations of medical treatment; stigma; cost of activities; GP time constraints; difficulty tracking activity options; concerns about volunteer competency; voluntary-sector turnover | Activities were mainly run by community organizations; many groups were involved in decision-making; third-sector organizations were not strongly regulated; past experiences with a similar program created doubt among some staff. | Evidence gaps concern demonstrating measurable health or well-being change, defining the link worker role, developing simple referral tools, supporting low-income participants financially, ensuring safe information-sharing, and reducing dependence on medical-only approaches. |
| Brettell et al., 2022, UK [8] | Link worker-mediated social prescribing with holistic, co-designed support, including social activities, housing, and financial advice. | National Health Service-commissioned service delivered through primary care networks and third-sector partners. | Young people aged 16-25 experiencing isolation, socioeconomic challenges, or mild mental health issues. | Measured: Improvements in loneliness, mental well-being, happiness, life satisfaction, calmness, and optimism. Perceived: Better social connectedness, usefulness, meaningful daily activity, and relationships. System: Reduced perceived need for support regarding finance, housing, and emotional issues. | Person-centered co-design; weekly link worker contact; flexible delivery; multi-agency accessibility | Small sample; no comparison group; selection bias; inconsistent network outcomes; self-report limitations | The study was set in an area with high youth mental health needs; many young people were waiting for specialist care; community services varied by location. | Research should expand to larger and more diverse youth samples, track long-term mental health outcomes, include cost data, and incorporate randomized comparisons with usual care. |
| Brown & Aylett, 2025, UK [87] | Nature-based social prescribing, including outdoor volunteering, nature activities, well-being walks, community farm engagement, and mindfulness in green spaces. | Community delivery by third-sector providers; referrals from primary care, community connectors, and local coordinators. | Adults with mental health challenges, long-term unemployment, social disadvantage, disabilities, and limited readiness for group activities. | Perceived: Improved well-being, reduced anxiety, strengthened social connectedness, greater community resilience, and perceived value to the health system. No measured outcomes reported. | Person-centered approach; trusted relationships; strong local partnerships; alignment with health-promotion policies | Funding insecurity; unclear referral pathways; low visibility; limited access for complex needs; staff turnover; no standard evaluation methods; inconsistent terminology | A national framework for social prescribing was being developed; services varied across regions; there was tension between medical and community approaches; people in rural areas had fewer options. | Future priorities include clear referral eligibility criteria, exploration of long-term funding pathways, simple progress-recording systems, improved inter-organizational collaboration, better reach to high-need groups, and system-wide impact assessment. |
| Cailhol et al., 2024, France [16] | Social prescribing integrated into discharge coordination using a link worker providing personalized navigation, referral to non-governmental organizations, language-matched general practitioner access, administrative support, and follow-up. | University hospital discharge pathway in two medical departments (endocrinology and infectious diseases). | Socially deprived inpatients with complex medical and social needs; a high proportion of migrants and individuals not fluent in French. | Measured: No significant change in unplanned hospital readmission; a large proportion with unmet social prescribing needs. Perceived: Improved discharge understanding, high trust and satisfaction, broader access to community services. | Shared cultural background; clinical communication skills; strong local networks; person-centered goal setting; continuous relational engagement | Staff resistance; poor cross-sector coordination; short duration; pandemic disruption; unsuitable outcome measures | The area had high levels of deprivation and many languages; laws limited the collection of ethnicity data; the health and social sectors were separate; the new discharge coordinator role had not yet been established. | Future work should develop accurate tools for identifying social needs, use broader health indicators beyond hospital readmission, include long-term and financial evaluations, examine optimal training approaches for discharge coordinators and link workers, and study how hospitals and social services can collaborate more effectively. |
| Calderón-Larrañaga et al., 2023, UK [17] | Primary care-based link worker social prescribing with referrals to lifestyle programs, physical activity, weight management, language support, welfare advice, and social support. | General practitioner practices and voluntary and community sector organizations; remote delivery during the COVID-19 pandemic. | Adults eligible for social prescribing include individuals with high risk for type 2 diabetes, multimorbidity, mental health needs, deprivation, and minority ethnic backgrounds. | Measured: High-risk adults are more likely to receive referral; greater reach among socially deprived and multimorbid individuals; higher uptake compared with the disease-prevention program. Perceived: Improved lifestyle behaviors, confidence, reduced isolation, better navigation of services, and a strong connection with link workers. | Broad eligibility; embedded link workers; access to medical records; proactive follow-up; holistic assessment; established community partnerships | Variable engagement; socioeconomic constraints; preference for medical care; workforce turnover; digital barriers | The area had high deprivation and many ethnic groups; general practitioners had strong links with community groups; the pandemic increased pressure; local policies supported social prescribing. | Priority areas include long-term assessments of diabetes prevention outcomes, testing the program in new settings, improving ways to measure participation and engagement, examining whether the model reduces health inequalities, and replacing short-term pilots with longer-term implementation frameworks. |
| Calderón-Larrañaga et al., 2024, UK [111] | Primary care-based link worker social prescribing with referrals to activities such as welfare advice, benefits, housing, weight management, physical activity, and general well-being support. | General practitioner practices and primary care networks with embedded link workers; voluntary and community sector program delivery; remote components during the COVID-19 pandemic. | Adults aged 18 and older with non-clinical needs; high-risk adults for type 2 diabetes; socioeconomically deprived and ethnically diverse groups. | Measured: No quantitative outcomes. Perceived: Differences across practices, including sustained personalized support, limited provision due to eligibility and capacity constraints, and signposting without the ability to address complex needs. | Flexible roles; shared experience; strong general practitioner-community relationships; reflective spaces; practitioner autonomy; organizational support | Short-term commissioning; capped sessions; restrictive eligibility; heavy monitoring; staff shortages; fragmented services; turnover; remote-access challenges; emotional strain | The area had long-term experience with social prescribing; community organizations were well developed; social prescribing was encouraged to reduce demand on services; the pandemic disrupted normal operations. | Evidence gaps relate to understanding what happens during link worker encounters, developing monitoring tools that reflect relationship-building work, evaluating flexible funding and commissioning approaches, assessing effects on health inequality, and identifying strategies to prevent practitioner burnout. |
| Calderón-Larrañaga et al., 2026, England [67] | National primary-care social prescribing referral model linked to NHS social prescribing services. Referral may include link worker support, signposting, needs assessment, ongoing support, lifestyle programs, welfare advice, community engagement initiatives, physical activity, social or cultural groups, and other voluntary/community-sector services, depending on local availability and patient need. | English primary care, using routinely collected data from the Royal College of General Practitioners Research and Surveillance Centre, covering more than 1,800 general practices. The model reflects NHS England's national social prescribing link worker program and primary care network-based roll-out from 2019 onward. | Adults aged ≥18 years at high risk of type 2 diabetes, including those with non-diabetic hyperglycemia, pre-diabetes, elevated HbA1c or fasting glucose, high QDiabetes risk score, history of gestational diabetes, or NHS Diabetes Prevention Program referral. | Health: Incident type 2 diabetes was the main clinical outcome. Social prescribing referrals were associated with lower type 2 diabetes risk over time, especially by 2022–2023 and after ≥3 years of follow-up. System/equity: referrals reached people with greater health and social need, including more deprived groups, people with obesity, multimorbidity, smoking, and depression/anxiety. Equity gaps were observed for Asian groups and younger adults. | National NHS roll-out; embedding social prescribing within primary care; referral reach into deprived and high-need groups; link worker infrastructure; holistic offer beyond lifestyle advice; potential integration with NHS Diabetes Prevention Program; sustained relationship-based support; local community networks; return to in-person services after COVID-19; service maturation over time. | Lack of data on actual participation, activity type, dose, intensity, quality, modality, or adherence after referral; possible residual confounding; guarantee-time bias; incomplete capture of health behaviors; missing data for some clinical variables; lower referral among Asian groups and younger adults; COVID-19 disruption and early remote delivery; variation in local social prescribing models and recording practices. | Conducted during the rapid national expansion of social prescribing in England following the NHS Long Term Plan and primary care network roll-out. The study covers the COVID-19 period and post-pandemic service maturation. Social prescribing was positioned as a complementary, holistic preventive approach for long-term condition prevention, especially for people with complex social and clinical needs. | Future research should identify which social prescribing components, content, intensity, modality, duration, and link-worker practices drive benefit; measure engagement beyond referral; link social prescribing data with NHS Diabetes Prevention Program attendance and medication data; use time-aware quasi-experimental designs; monitor referral equity; co-design culturally tailored approaches for Asian communities and younger adults; and evaluate long-term durability and cost-effectiveness. |
| Carnes et al., 2017, UK [76] | Link worker social prescribing with coordinators providing up to six individual sessions, volunteer support, personalized plans, and referrals to a wide range of community organizations. | General practitioner (22 GP) practices with delivery by a third-sector organization and additional community-based volunteer support. | Socially isolated adults, frequent primary care users, and adults with anxiety, depression, chronic illness, or social problems. | Measured: No significant changes in physical or mental health indicators; decrease in general practitioner visits likely unrelated to intervention; stable medication use. Perceived: Improved confidence, motivation, and daily routine; strongest benefits among participants receiving multiple sessions. | Coordinators embedded in general practices; strong interpersonal skills; relational support; shared goal setting; continuity with volunteers; available activities | Low engagement; crises reducing participation; low understanding of social prescribing; unclear terminology; inconsistent attendance; limited participation data; poor survey response | The setting had high deprivation and many ethnic groups; general practitioners had heavy workloads; the quality of community organizations varied; some people had difficulty with written English; social prescribing was still new in the area. | Further research should improve measures of social connectedness and relationships, determine how much program exposure leads to benefits, use more rigorous trial designs, strengthen public communication about the purpose of social prescribing, and explore referral timing and GP perspectives. |
| Cheshire et al., 2022, UK [83] | Ten-week structured group yoga program including psychoeducation, breathing exercises, postures, relaxation, group discussion, and home-practice support. | Community venues within National Health Service Clinical Commissioning Groups; referrals from general practitioners and community organizations. | Adults at risk of cardiovascular disease, pre-diabetes, anxiety, depression, or social isolation. | Measured: Not reported. Perceived: Reduced stress and anxiety, improved emotional regulation, mood, flexibility, mobility, sleep, energy, pain, physical activity, diet, social connection, and self-management; some improvements remained after 3 months. | Inclusive adaptations; skilled facilitators; strong group cohesion; accessible psycho-education; structured weekly format; supportive referral pathways | Time demands; discomfort with venues; scheduling issues; limited follow-on options; mismatched challenge levels; anxiety triggers; difficulty sustaining practice | The program followed the national social prescribing model; many participants had long-term health conditions; activities were delivered in community spaces; cultural beliefs affected participation; most participants were women. | Future priorities include trials comparing the program with usual care, studies assessing whether benefits are sustained post-program, development of smoother follow-up pathways, identification of the most effective components of the breathing intervention, inclusion strategies for men and other under-represented groups, and outcome tools aligned with what patients value. |
| Chng et al., 2021, UK [123] | Deep End Links Worker Program with full-time community link practitioners embedded in general practitioner practices to provide patient support, practice development, and community networking. | General practitioners practice in highly deprived areas with on-site community link practitioners. | Adults in high-deprivation areas with complex social and health needs. | Measured: None. Perceived (system and implementation): Improved integration in some practices, clearer role definition, better teamwork, greater learning, enhanced links between practices and community organizations. | Shared leadership; strong team relations; role clarity; whole-team engagement; consistent practitioner support; protected learning time; active community networking | Poor shared understanding; fragmented leadership; restricted role; low engagement; turnover; competing initiatives; limited time; lack of monitoring | The program was part of a government pilot; practices in deprived areas had heavy workloads; link workers were employed by a community organization; other system changes were happening at the same time. | Key gaps involve long-term implementation research, identification of necessary organizational conditions, strengthened monitoring systems, assessment of long-term sustainability, and examination of whether the program reduces health inequalities. |
| Christiana et al., 2025, United States [89] | Nature prescription / outdoor physical activity prescription program using the Kids in Parks TRACK Rx model; school nurse prescribed outdoor activities and TRACK Trails, with students selecting preferred activities and dosage | Two rural elementary schools in North Carolina; one intervention school and one control school; intervention delivered by the school nurse in 1st–3rd grade classrooms, with reminder emails sent to parents at 1 and 2 months | Children in 1st–3rd grade and their parents/guardians in a rural, economically distressed county; intervention targeted children's outdoor time and nature-based physical activity | Health: significant increase in weekend outdoor physical activity in the intervention group; no significant change in overall physical activity, weekday outdoor physical activity, or total time spent outdoors. Social/system: high parental trust in school nurses; evidence that school nurses can act as credible prescribers of nature-based physical activity within school health systems | Student-centered prescription process; school nurse credibility and access to children; individualized activity selection; written prescription and student–nurse signature; local TRACK Trails and parks; reminder emails to parents; alignment with school nursing leadership, care coordination, quality improvement, and community/public health roles | Small convenience sample; parent-reported outcomes; short 3-month follow-up; seasonality between baseline and follow-up; limited direct parental engagement; prescription alone may be insufficient without wider school and community supports; no significant effect on several outcomes | Conducted in a rural Tier One economically distressed North Carolina county; schools were near TRACK Trails and outdoor recreation opportunities; intervention was delivered at the end of the school year before summer break; school nurses may reach children who have limited access to primary healthcare | Future research should use larger and more diverse samples, objective measures such as accelerometry, pedometry, and GPS, longer follow-up, and stronger integration of TRACK Rx into physical education, recess, classroom curricula, after-school programming, Safe Routes to School/Parks, and organized outdoor group activities. |
| Dadswell & Bungay, 2025, UK [84] | Cultural social prescribing across arts, museums, libraries, heritage, digital creativity, food, green spaces, and sport, delivered through link workers and informal connectors. | Chelmsford district's social prescribing system, including National Health Service primary care, voluntary organizations, and cultural sector partners. | Adults and young people, including marginalized groups such as refugees, ethnic minorities, low-income groups, disabled individuals, older adults, and socially isolated people. | Measured: None. Perceived: Increased understanding of social prescribing, recognition of well-being benefits from cultural participation, identification of unmet service needs, improved confidence and social connection. | Shared terminology; community outreach; trusted community leaders; volunteer support; inclusive communication; co-production; strong networks | Confusing terminology; unclear roles; literacy barriers; financial barriers; transport barriers; uneven cultural provision; limited funding; access challenges; fear of judgement; inconsistent pathways | The area had a mix of health and community organizations; the high cost of living affected participation; volunteers were essential; cultural social prescribing was still new. | Research should focus on co-designing models with community members, identifying which types of cultural programs improve health, reaching socially excluded populations, securing stable funding for arts and culture providers, evaluating capacity within community venues, and creating frameworks for culturally appropriate delivery. |
| Darcy et al., 2025, England [33] | Green social prescribing involving referral by social prescribing link workers or equivalent professionals to nature-based interventions, including horticulture, care farming, green exercise, bushcraft, conservation, creative outdoor activities, photography, crafting, yoga or mind-body activities, and other green or blue community activities. | Humber and North Yorkshire Green Social Prescribing "test and learn" program; referrals came through social prescribing services embedded in NHS primary care, secondary care mental health services, VCSE-sector services, and local authority-led services. Nature-based interventions were delivered by community and VCSE providers. | Adults aged ≥18 years experiencing mild to moderate mental health problems, including anxiety, depression, low mood, social anxiety, phobias, bereavement, work-related stress, sleep difficulties, and related psychosocial needs; participants were required to be motivated and able to engage with green activities without one-to-one support. | Measured health/well-being outcomes: significant improvements in life satisfaction, feeling worthwhile, happiness, ONS-4 anxiety, HADS anxiety, and HADS depression. Social outcomes: engagement with nature-based and community activities. System outcomes: evidence supporting green social prescribing as a community-based response to mental health need and potential pressure on mental health services. | "What matters to you" conversations; availability of a menu of vetted nature-based activities; link worker or equivalent referral support; community and VCSE-sector delivery; longer intervention duration; horticulture and care farming activities; tailoring activities to physical health needs where possible; cross-sector collaboration between health, local authority, and VCSE partners. | No randomized or controlled design; risk of selection bias; 22% lost to follow-up; self-referral was the most common pathway, suggesting weaker formal referral mechanisms; limited participation by some underserved groups; sustainability challenges due to short-term funding; heterogeneous referral pathways and intervention models; lack of detailed evidence on which pathway components worked best for whom. | Part of the NHS England Green Social Prescribing "test and learn" program funded through the UK government's Shared Outcomes Fund after COVID-19. The program operated in a context of high unmet mental health needs, socioeconomic deprivation, pressure on mental health services, and policy interest in nature-based community interventions. Many participants lived in highly deprived areas and reported mental health conditions and functional limitations. | Future research should use controlled or randomized designs, track referral pathways from healthcare to community assets, assess long-term outcomes, examine which activity types and durations work best for different groups, evaluate equity of access and uptake, explore mechanisms of benefit, map local green assets, and develop sustainable funding and implementation models for VCSE-delivered green social prescribing. |
| Davis, 2026, Scotland/United Kingdom [86] | Dance-on-prescription / arts-on-prescription model linked to Scottish Ballet's SB Health neurological programs, including Dance for Parkinson's Scotland, Time to Dance® for dementia, and SB Elevate® for people with multiple sclerosis. Pathways included self-referral, active signposting, community links workers, secondary care referral routes, and hybrid or virtual engagement. | Scottish Ballet's Glasgow headquarters and regional pathways across NHS Greater Glasgow and Clyde, NHS Tayside, and NHS Orkney; pathways involved Scottish Ballet, the Health and Social Care Alliance Scotland, NHS primary and secondary care, community links workers, allied health professionals, neurological services, and third-sector partners. | People with neurological conditions, particularly Parkinson's disease, dementia, and multiple sclerosis, the study itself collected data from cross-sector professionals rather than service users. | Health/social: professionals perceived dance provisions as supporting physical activity, self-management, social connection, confidence, person-centered care, and well-being among people with neurological conditions. System: findings focused on professional adoption, cross-sector coordination, referral pathway development, cultural-sector credibility, and the role of a national cultural asset in social prescribing. No direct participant outcome data were collected. | Scottish Ballet's cultural credibility; evidence-informed dance health programs; Health Partnerships Manager role; support from the ALLIANCE; strategic relationships; professional awareness-raising; first-hand professional experience of dance sessions; tailored terminology aligned with health priorities; clinical leadership support; secondary care neurological pathway in NHS Tayside; hybrid delivery options; advocacy through Healing Arts Scotland. | Limited professional capacity; inconsistent coordination across services; reliance on individual champions; weak service-level agreements; variable awareness and confidence among referrers; limited relevance for generic primary care link workers; cultural perceptions that ballet is "not for them"; mobility, transport, digital, and financial access barriers; sustainability concerns; pressure on NHS resources; differences in governance and evidence expectations between arts and health sectors. | Conducted in Scotland, where social prescribing pathways vary across regions and are less uniformly structured than NHS England's primary-care link-worker model. Scottish Ballet operates as a national cultural asset seeking to connect arts provision with health and social care. The study reflects growing policy interest in arts-on-prescription, cultural assets, neurological conditions, and cross-sector models of person-centered care. | Future research should examine patient access and outcomes, compare different prescribing pathways, assess whether specialist secondary care routes are more effective than generic primary care routes for condition-specific arts provision, evaluate sustainable funding models, clarify role boundaries between cultural and health-sector actors, and explore how national cultural assets can support smaller local cultural providers without overextending into health-service responsibilities. |
| Dayson et al., 2020, UK [94] | Holistic social prescribing tailored to secondary mental health, with link workers connecting individuals to non-clinical community activities, including peer support and volunteering. | Secondary mental health services and voluntary and community sector organizations supporting discharge. | Adults with severe or long-term mental health conditions and individuals experiencing social exclusion. | Measured: Increased positive emotions and life satisfaction; increased sense of purpose, personal growth, autonomy, and ability to manage daily life; reduced social isolation; stronger social networks; more peer and volunteering activity. System: Higher likelihood of discharge from secondary mental-health services. | Structured six-month transition; strong partnership with voluntary sector; flexible person-centered delivery; grant funding; informal settings; broad activity menu | Non-uptake; long readiness periods; difficult life circumstances; limited sector resources; higher intensity needs | The program was based in secondary mental health services; progress often needed long timelines; success depended on the capacity of community groups. | Evidence gaps include long-term mixed-methods evaluations, identification of patients who benefit the most, understanding optimal program duration, assessing potential for scaling the model, and determining whether community organizations can manage workload demands over time. |
| Di Lorito et al., 2025, UK [51] | Social prescribing delivered by link workers, care coordinators, well-being coaches, and community connectors, including dementia-sensitive adaptations such as motivational interviewing, gradual goal setting, home visits, and culturally sensitive communication. | Primary care, voluntary sector organizations, local councils, private home-care agencies, and some secondary care services. | People living with dementia at all stages; informal carers indirectly involved. | Measured: None. Perceived (implementation): Improved engagement with appropriate dementia approaches, clearer needs identification, better communication, and better continuity across organizations. | Dementia-specific training; home visits; family involvement; interpreter support; accessible language; reminders; structured prioritization; strong partnerships; ongoing training | Poor referral information; weak communication; lack of dementia training; reliance on remote consultations; digital exclusion; cultural barriers; client resistance; difficulty assessing needs; dependency; uneven community assets; high workload; emotional strain | The care system was fragmented; some community providers could not access medical records; roles between clinical and non-clinical staff were unclear; remote care was influenced by cost pressures. | Future research should compare dementia-specialist and generalist link workers, incorporate perspectives from people with dementia and carers, conduct cost evaluations, test dementia-specific program versions, identify effective communication strategies, compare remote vs in-person delivery, and examine how dementia social prescribing can be integrated into routine care. |
| Dingle et al., 2024, Australia [28] | Link worker social prescribing with assessment, motivational support, and referral to community group activities. | General practitioner clinics, community centers, and combined primary care-community hubs. | Adults experiencing loneliness across diverse cultural and socioeconomic groups. | Measured: Improvements in loneliness, well-being, anxiety, psychological distress, and social trust; changes in health service use. Perceived: Increased sense of connection and reduced distress. | Varied community programs; active link worker engagement; personalized assessment; broad referral routes | Non-randomized design; short follow-up; pandemic disruption; model variation; differences between groups | The program covered multiple sites; pandemic restrictions affected delivery; the population was culturally diverse; the comparison group was not equivalent. | Priority areas include studies with appropriate comparison groups, more extended follow-up periods, more accurate loneliness measurement tools, and comparisons with other loneliness-reduction interventions. |
| Donaghy et al., 2025, UK [114] | Social prescribing link worker schemes including signposting, multi-session support, referral coordination, and assistance with social needs. | Primary care networks, general practitioner practices, voluntary and community sector partners, and integrated care systems. | Broad adult population including individuals with multimorbidity, mental health needs, social isolation, poverty, or refugee and asylum seeker status. | Measured: Changes in well being, life satisfaction, activation, and health-related quality of life. Perceived: Improvements in social needs, daily functioning, and emotional well-being that were not fully captured by standard measures. | Strong community-sector partnerships; flexible delivery; embedded link workers; multi-agency collaboration; local tailoring | Inconsistent data systems; variable tools; unclear boundaries; session limits; financial pressures; inconsistent referrals | There were large differences across regions; financial cuts affected services; rural and urban areas had different needs; voluntary-sector funding was unstable; the pandemic changed how services operated. | Future priorities include creating simple outcome tools that are consistent across services, developing social-prescribing measures designed for front-line staff, comparing short-support and intensive-support models, tracking outcomes after discharge over the long term, and conducting financial evaluations to assess program sustainability. |
| Donaghy et al., 2026, UK (England and Scotland) [141] | Generalist social prescribing link worker model embedded in general practice, with variation in employment, organization, and management models. SPLWs provided relational support, advocacy, practical assistance, and links to VCSE and community resources. | General practices and primary care systems across two English regions and two Scottish regions; delivery involved primary care networks, health and social care partnerships, NHS structures, and VCSE organizations employing, hosting, or supporting SPLWs. | Adults receiving SPLW support, including people with complex social, financial, housing, mental health, addiction, social isolation, and multimorbidity-related needs; many patient participants lived in areas of high socioeconomic deprivation. | Perceived: Improved patient confidence, independence, community engagement, and access to practical and community support. System: Reduced moral distress among general practitioners and possible reductions in GP workload, although workload effects were mixed. SPLW activity increasingly addressed social determinants of health and complex unmet needs that were not routinely captured in primary care data. | Integration into one or two general practices; regular room or presence in practice; robust organizational support; supportive employer and SPLW networks; GP/referrer support; time to build therapeutic relationships; empathy and listening skills; training, supervision, and support; clearer role boundaries; strong links with VCSE organizations. | Short-term funding; increasing service demand; financial cuts to statutory and voluntary services; role ambiguity; lack of standardization; job retention difficulties; burnout; variable pay; limited career progression; increasing referral complexity; gaps in mental health, housing, welfare, and statutory support. | Conducted across England and Scotland during a period of primary care pressure, health inequalities, socioeconomic deprivation, economic austerity, and financial pressure on statutory and voluntary sectors. Delivery structures differed across English and Scottish regions, with variation in SPLW employment, organization, management, and integration into general practice. | Future research should examine the long-term influence of SPLWs on health inequalities, prevention, patient outcomes, GP workload, and system-level effects across different models and contexts. Further work is needed on sustainable funding, role clarity, workforce support, and routine capture of social determinants-related SPLW activity. |
| Du Plooy et al., 2025, Aotearoa New Zealand [34] | Green Prescription program; primary-care-based physical activity prescribing with individualized support, goal setting, and referral or connection to subsidized community-based physical activities | Green Prescription Otago program in Aotearoa New Zealand; referrals from general practitioners, specialists, and self-referral; delivered through consultations with exercise professionals and links to community-based activities in urban Dunedin and rural Central Otago | Adults who would benefit from increased physical activity, including people with chronic physical conditions, mental health challenges, mobility limitations, diabetes, arthritis, seasonal depression, and socioeconomic constraints | Health: perceived increases in physical activity, improved mental health, enhanced confidence, hope, self-efficacy, diabetes management, seasonal mood, and quality of life. Social: social connection through group activities, peer support, and family involvement. System: perceived need for better follow-up, stronger community partnerships, longer support duration, and improved program visibility | Strengths-based and individualized support; supportive staff; subsidized access to activities; goal setting; flexible activity options; social/group-based activities; family involvement; community activity links; positive framing around what participants could do rather than restrictions | Financial constraints despite subsidies; transport difficulties, especially for rural participants; complex medical conditions and medication effects; chronic pain and mobility limitations; COVID-19 disruption; confusion about program name; insufficient in-person support for some; difficulty sustaining activity after formal support ended | Long-running national program operating for more than 25 years; Otago context included both urban and rural populations; participants had diverse socioeconomic circumstances; some were on fixed incomes or benefits; program duration was typically 3–6 months; outdoor and community-based physical activity were central but not exclusive elements | Future research should examine optimal program duration and intensity, compare in-person, digital, hybrid, and tiered delivery models, include economic evaluation and participant-reported outcomes, track long-term sustainability beyond 2 years, include healthcare provider and program staff perspectives, investigate non-participation, and explore culturally responsive delivery for Māori and Pasifika populations. |
| Elston et al., 2019, UK [29] | Twelve-week intensive link worker model including assessment, goal setting, coaching, emotional support, practical assistance, and community navigation. | Integrated care organization with primary, community, and social care hubs; home-based components; voluntary-sector partners. | Adults aged 50 and older with multimorbidity, frailty, low activation, and complex social needs. | Measured: Improvements in well-being, activation, and frailty; mixed findings regarding use of services; increased costs among individuals with high needs. Perceived: Reduced isolation and improved support for carers. | Strong relational engagement; home-based model; cross-sector co-location; integrated referrals; voluntary-sector partnership | No comparison group; incomplete GP data; heterogeneous referrals; missing follow-up; high-cost cases | The population was older and lived in rural or coastal areas; community care had been reduced due to budget cuts; the program was part of a larger system redesign. | Key gaps involve trials comparing the program with usual care, evidence on cost savings, analysis of how different patient groups respond, improved routine data collection, and comparisons of shorter versus more extended program versions. |
| Esfandiari et al., 2025, Canada [139] | Holistic social prescribing delivered by community connectors, including assessment, goal setting, coaching, referrals, accompaniment, and follow-up. | Community agencies linked with primary care across approximately twenty sites; mixed in-person and remote delivery. | Older adults with unmet social, housing, financial, or isolation needs; low-income and digitally limited groups. | Perceived: Better access to various supports, improved communication, enhanced service navigation, reassurance, and social support. System: Variation in reach and quality depending on available resources. | Strong rapport with connectors; partnerships with community agencies; volunteer support; flexible tailoring | Limited community assets; shortages in housing and transport; digital exclusion; few volunteers; inconsistent referrals; limited translation | The program was new at the provincial level; it covered both urban and rural settings; volunteers were heavily used; community resources varied widely across sites. | Research should define core link-worker tasks, establish shared tools and materials across sites, strengthen communication between healthcare teams and community organizations, evaluate volunteer roles, and plan for long-term funding stability. |
| Farina et al., 2026, Italy [47] | Youth-focused social prescribing intervention through the C.O.P.E. model: Capabilities, Opportunities, Places and Engagement. The intervention used Link Worker support to connect young people with relational, well-being-oriented opportunities in community, education, and employment. Rather than emphasizing rapid job placement, it provided personalized, flexible, and gradual support for psychosocial stabilization and labor-market readiness. | Autonomous Province of Trento, Italy, within a community welfare and relational proximity model involving public services, third-sector organizations, Link Workers, social services, employment services, mental health services, educators, families, and community actors. The C.O.P.E. intervention was piloted in Italy and Portugal, with this study focusing on the Italian pilot. | Young people aged 16–34 years in NEET situations, including those with mental health difficulties, social isolation, low educational attainment, long-term inactivity, family pressure, substance-use service involvement, social work support, psychiatric service involvement, or criminal justice system contact. | Perceived health/social outcomes included improved self-esteem, agency, emotional stabilization, self-understanding, confidence, future orientation, motivation, reduced isolation, and increased readiness to engage with education or employment pathways. System-level outcomes included evidence that social prescribing can bridge well-being support and labor-market engagement through relational, low-threshold support. Immediate employment was not the main outcome; benefits were gradual, psychosocial, and preparatory. | Flexible and non-judgmental Link Worker role; trust-building; relational proximity; personalized pacing; low-threshold entry; emotional protection; practical support with CVs, job searching, interviews, and pathway planning; coordination with social, mental health, employment, and community services; attention to readiness rather than rapid activation; family or professional referral routes; protected space for rebuilding agency. | Low initial motivation; external pressure to participate; fear of failure; low self-confidence; anxiety and panic symptoms; social isolation; long-term inactivity; previous negative labor-market experiences; fragmented services; structural labor-market insecurity; limited trust in institutions; risk that rapid activation models may worsen disengagement; small qualitative sample and lack of long-term employment outcome data. | Conducted in the Italian welfare context, where community-based and relational approaches are increasingly used to address social vulnerability. The intervention responds to the NEET phenomenon as a combined social, mental health, and labor-market issue rather than a purely employment-related problem. The study highlights the health–work paradox: employment is important for well-being, but many vulnerable young people first need psychosocial stabilization and relational support before they can engage with work or training. | Future research should use longitudinal and mixed-methods designs, include young people who disengage or refuse participation, assess longer-term education and employment outcomes, include Link Worker and service-provider perspectives, examine gender and vulnerability differences, compare C.O.P.E. across welfare contexts, and evaluate how social prescribing can be integrated with employment, mental health, and social services for young people in NEET situations. |
| Fixsen et al., 2020, UK [18] | Link worker social prescribing with co-produced plans and referrals to voluntary, community, and social enterprise activities, including physical activity, arts, gardening, welfare, and social support. | Rural general practitioner practices, community venues, government offices, and adult social care settings. | Adults with cardiovascular risk, loneliness, multimorbidity, and complex social needs in rural settings. | Perceived: Increased well-being, confidence, motivation, and social connectedness; some lifestyle changes reported. Measured outcomes not specified. | Strong link-worker relationships; skilled staff; multi-sector collaboration; existing community assets; supportive leadership | Transport barriers; mobility limitations; activity costs; short program duration; data challenges; GP skepticism; difficulty engaging isolated individuals | The area had rural deprivation; transport options were limited; teams were small; local inequality affected engagement; and the program was still in an early stage of development. | Priority areas include identifying effective models for rural settings, supporting people with low motivation or confidence, improving connections between general practice and community services, enabling digital access, and tracking outcomes over extended periods. |
| Fixsen et al., 2021, UK [96] | Three social prescribing models: general practitioner-embedded link workers, the community-based SPRING program, and the rural digital program, offering signposting, well-being sessions, education workshops, and digital support. | General practitioner practices, community centers, and rural digital outreach locations. | Adults with complex needs, adults with low-level mental or social support needs, and older adults with long-term conditions or digital barriers. | Perceived: Improved digital inclusion, continued social contact, access to necessary resources, consistent support during the pandemic; no measured outcomes reported. | Person-centered support; general practitioner trust; flexible adaptation; provision of digital devices; interdisciplinary collaboration | Digital exclusion; language barriers; heavy service demand; socioeconomic hardship; limited voluntary-sector capacity; unclear roles | Most services were delivered remotely due to the pandemic; the area already faced deprivation; community services had been shrinking; waiting times in healthcare were long. | Future work should compare digital and in-person delivery, clarify job descriptions for link workers, include minority ethnic groups, standardize outcome measures, and develop preparedness plans for future service disruptions. |
| Fixsen & Barrett, 2022, UK [79] | Green social prescribing, including walking, gardening, outdoor groups, nature-based arts, and general social prescribing referrals. | Primary care, third-sector organizations, and community parks and venues in Scotland and Northeast England. | Adults with non-medical needs, including older adults, individuals with multimorbidity, chronic illness, anxiety, and social isolation. | Perceived: Improved well-being, reduced loneliness, increased social interaction, more physical activity; some participants avoided activities due to post-pandemic anxiety. | Engagement from general practitioners, accessible green spaces, and inclusive community activities | Transport barriers; mobility limits; digital exclusion; reduced third-sector capacity; weather constraints | The area had high levels of deprivation and rural isolation; public transport was limited; pandemic restrictions affected deliveries. | Evidence gaps include urban-rural comparisons, strategies for engaging people who live far from services, long-term post-COVID evaluations, and financial planning for community organizations that cannot depend on short-term grants. |
| Foster et al., 2021, UK [69] | National social prescribing service with link workers and volunteers providing twelve weeks of assessment, confidence-building, befriending, and signposting. | Thirty-seven sites, including community venues, voluntary-sector centers, home visits, telephone, and video delivery. | Adults at risk of loneliness, including young parents, bereaved individuals, older adults, and people living alone. | Measured: Reduced loneliness, improved well-being, reduced missed appointments, reported positive social return on investment. Perceived: Increased confidence, social connection, and daily functioning; partial decline at follow-up but improvements remained above baseline. | Skilled link workers; personalized support; broad referral menus; flexible appointments and locations | Volunteer shortages; limited activity options; transport barriers; mobility issues; high disengagement; missing routine data | Community capacity had been reduced by financial cuts; needs varied across different areas; there was high demand for support related to loneliness. | Evidence gaps relate to planning ongoing support after program completion, establishing regular follow-up systems, strengthening routine record-keeping, comparing different program intensities, and conducting financial evaluations. |
| Giebel et al., 2021, UK [80] | Community-based social prescribing with personalized assessment, signposting, weekly support, transport assistance, and volunteer community champions. | Third-sector organization with a community drop-in center; referrals from general practitioners and adult social care. | Middle-aged and older adults experiencing isolation, frailty, mobility barriers, or financial constraints. | Perceived: Reduced loneliness, improved social connection, enhanced well-being, better sleep, more physical and social activity, and greater engagement with community groups. | Easy self-referral; accessible drop-in venue; rapid assessment; person-centered approach; trusted community champions; wide referral options | Low awareness; transport costs; financial limits; stigma; physical challenges; unstable funding; limited diversity | The area had socioeconomic disadvantages; there was strong cooperation between sectors; volunteers were central to delivery. | Future research should generate numerical health and well-being outcomes, test approaches in more diverse populations, develop sustainability plans, provide practical implementation guidance, and examine long-term health improvements. |
| Giebel et al., 2022, UK [125] | Weekly well-being classes including low-impact exercise, Tai Chi, walks, mindfulness, games, and optional leisure-center activities. | Community and leisure center in collaboration with the National Health Service, the local council, and dementia navigators. | People living with dementia and their family carers. | Measured: Improved well-being at 3 and 6 months. Perceived: Increased social engagement, sense of belonging, reduced isolation. | Multi-agency partnership; navigator support; free access; flexible drop-in model; varied activities | Transport barriers; single-site access; competing commitments; small sample; no comparison group; attrition | The setting was deprived; participants depended heavily on public transport; the program was in an early development stage. | Key priorities include multi-site studies, larger samples, separate analyses for people with dementia and carers, comparison groups, and strategies to address transport barriers. |
| Golden et al., 2023, USA [42] | "CultureRx" arts-on-prescription model, including museum visits, dance and music classes, art sessions, and nature activities, with free access through clinical prescription. | Twelve cultural organizations partnered with twenty healthcare providers. | Children, adults, and older adults, including refugees, individuals with Parkinson's disease, mental health clients, and socially isolated groups. | Measured: Improved well being, mood, and mobility among individuals with neurological conditions; positive activity evaluations. Perceived: Reduced stress, stronger social connectedness, and increased confidence. | Clear communication; free access; flexible scheduling; take-home materials; multiple referral channels; funding support | Transport barriers; digital literacy issues; language barriers; scheduling limits; cultural relevance issues; pandemic restrictions | The COVID-19 pandemic disrupted delivery; the program depended on temporary grants; organizations had different levels of capacity; and social prescribing was still new in the United States. | Gaps include consistent evaluation tools, long-term participant tracking, integrated digital referral systems linking hospitals and community providers, approaches to engage underserved groups, secure financial support, and studies assessing clinical health outcomes. |
| Golubinski et al., 2020, Germany [65] | General practitioner-referred community health advice and navigation, including needs assessment, lifestyle education, group programs, and linkage to community resources. | Community health advice center in a deprived urban area staffed by multilingual health professionals. | Adults with lifestyle, chronic disease, mental health, or social and administrative needs in a multicultural community. | Measured: Increased return visits to primary care; higher adherence among individuals with overweight; lower adherence among those with long travel distances. Perceived: Professional authority encouraged engagement. | Trust in general practitioners; personalized plans; multilingual staff; free access; structured service directories | Low adherence among those with psychological concerns; distance barriers; limited referral information; no link worker model; single-site constraints | The program took place in a deprived area; people could access services directly without general practitioner referral; the center was funded by insurers; the organization used both nonprofit and for-profit structures. | Future research should provide detailed emotional, social, and practical outcome data; compare this model with programs using link workers; identify reasons for non-attendance; and use broader outcome sets beyond attendance and basic engagement. |
| Gordon et al., 2025, England [72] | Hospital in-reach, family-centered link worker, social prescribing. Link workers worked collaboratively with children and families to identify needs and goals, provide personalized support, and connect families to community resources over six months after hospital identification/discharge. | Great North Children's Hospital, Newcastle upon Tyne, North-East England; link workers were co-located in a children's hospital and employed by an external community/social prescribing organization, with support extending into the community after discharge. | Children aged <16 years with neurodisability and complex chronic needs, plus parents/carers and siblings. Conditions included cerebral palsy, epilepsy, autism, developmental delay, genetic syndromes, spina bifida, neuromuscular and neurodegenerative disorders. | Measured: Significant reduction in unmet need using the Support Star; most goals were fully or partially achieved; positive SROI of £2.75 per £1 spent. Some child well-being scores improved, but quality of life and parental well-being measures did not change significantly over six months. Perceived: families felt less isolated, more connected to community services, more supported, and more confident in navigating systems. System: link workers helped healthcare professionals address non-medical needs and may reduce pressure on clinical staff. | Co-location of link workers in the hospital; early identification during inpatient admission; trusted, empathetic, flexible, and knowledgeable link workers; family-centered goal setting; personalized "signposting plus"; practical help with forms/referrals; advocacy with community services; strong collaboration between link workers and hospital professionals. | Lack of initial understanding of the link worker role; complex family needs; parental exhaustion and limited "headspace"; language barriers and difficulty accessing translation; geographic eligibility restrictions; travel costs/logistics; limited community services suitable for children with complex needs; short six-month support duration; funding uncertainty. | The intervention addressed unmet social needs among families of children with complex neurodisability in a hospital setting, where care is usually medically focused. Families often experienced financial strain, social isolation, disrupted family life, repeated hospital admissions, and difficulty navigating non-medical support. The model was developed in a UK context where social prescribing is well established in adult primary care but underdeveloped for children, families, and hospital settings. | Future studies should test the model across multiple sites, include larger samples, evaluate longer support periods, develop family-based well-being outcome measures, examine scalability and commissioning models, include children with other forms of medical complexity, improve ways to capture child voice, and assess health service delivery and economic outcomes more robustly. |
| Gorenberg et al., 2023, UK [52] | Cultural-sector social prescribing, including museum, library, and garden programs with sensory and heritage activities delivered in person and online. | Cultural venues, including museums, libraries, and gardens, with online options. | Adults aged 60 and older experiencing loneliness, low well-being, or limited community engagement. | Perceived: Reduced stress, improved emotional well being, better social connectedness, positive experiences with online groups. System: Variation in coordination between link workers and providers. | Tailored offers; strong communication; buddy schemes; flexible participation; accessible venues; trained staff | Limited funding; emotional burden on staff; unclear boundaries; digital barriers; perceived exclusivity; weak coordination; low virtual engagement | Cultural organizations had uneven resources; the pandemic affected staffing and participation; volunteers played a significant role; expectations from the cultural sector differed from expectations in health services. | Key priorities include evaluating buddy-support approaches, tailoring activities to individual needs, determining best practices for virtual activity design, developing monitoring systems to track attendance and progress, and studying staff workload and long-term service sustainability. |
| Hamill et al., 2026, Australia [115] | RISE UP social prescribing model of care involving routine identification of unmet social needs and provision of referral/resource pathways. The model screened for five unmet social need domains: childcare, employment, housing, food security, and household bills, and provided families with a social care information resource regardless of disclosure status. | Pediatric allied healthcare intake service within the Child Health Information Link in Sydney Local Health District, Australia. The model was delivered during routine telephone intake for publicly funded pediatric medical, nursing, and allied healthcare services, including speech pathology, occupational therapy, physiotherapy, social work, psychology, early childhood social work, and dietetics. | Parents/carers of children aged 0–12 years referred for pediatric allied healthcare services; families included English-speaking, mixed-language, and non-English-only households. Child Health Information Link intake staff were also included in the acceptability and feasibility assessment. | Measured: unmet social needs, parent/carer acceptability, staff acceptability and feasibility, and sociodemographic factors associated with unmet need. Most families reported at least one unmet need; childcare and employment were most common. Parent/carer acceptability was very high, while staff acceptability and feasibility were moderate to low. System-level findings highlighted a reach gap between eligible families and those offered RISE UP, and the need to integrate social needs identification into pediatric allied healthcare access pathways. | Routine intake as a contact point for identifying social needs; person-centered screening; provision of resources regardless of disclosure; health literacy and equity-informed social care resource; consultation with local multicultural service staff; use of Accelerating Implementation Methodology; telephone delivery; parent/carer willingness to discuss unmet needs; recognition of allied health professionals' role in tackling health inequalities. | Staff workload and competing demands; staff shortages; workflow and information technology barriers; lower staff acceptability and feasibility; limited reach and representativeness; possible underrepresentation of non-English-speaking families; lack of tailored or translated resources for all communities; disclosure barriers; reliance on individual-level social needs screening; no outcome data on whether families' unmet needs improved after referral/resource provision. | Conducted in a culturally and linguistically diverse Sydney Local Health District, where pediatric allied healthcare intake may be one of the few opportunities to identify and address unmet social needs. The study was framed by child health inequities, social determinants of health, the inverse care law, rising child poverty, and the lack of routine social prescribing implementation in Australian pediatric allied healthcare services. | Future work should refine RISE UP with parent/carer and community input, conduct psychometric assessment of the screening tool, improve reach and equity, tailor and translate resources for diverse communities, strengthen workforce training and capacity building, evaluate impact on unmet needs, service access, utilization, child and family outcomes, and assess economic implications for families and health services. |
| Hanlon et al., 2021, UK [126] | One-to-one community link practitioner social prescribing, including assessment, support, and signposting. | Primary care in high-deprivation areas with community link practitioners embedded in general practice teams. | Adults aged 34-64 with multimorbidity, social isolation, and psychosocial issues. | Measured: Mixed levels of change in well-being and functioning. Perceived: Reduced isolation, improved well-being, increased confidence, and autonomy. | Trusted practitioner relationships; time availability; patient-led goals; flexible support | Health limitations; low readiness; few community groups; poverty; transport barriers | The area had high deprivation; community infrastructure was limited; and services showed an inverse care pattern, with areas of high need having fewer resources. | Evidence gaps involve collecting long-term outcome data, increasing sample sizes, designing programs that do not depend heavily on individual practitioners, and conducting evaluations grounded in clear theories of change. |
| Hassan et al., 2023, UK [121] | Multiple social prescribing models, including link workers, hubs, signposting, well-being activities, and personalized planning. | Primary care settings partnering with voluntary and community sector hubs and local authorities. | Adults of all ages with social, mental health, or long-term conditions need. | Measured: Improvements in well-being and personal goal achievement; progress on several process indicators. Perceived: Reduced loneliness and improved personalized support. | Co-design; strong link-worker relationships; asset mapping; multi-agency coordination; service co-location | High caseloads; inconsistent tools; volunteer shortages; limited asset capacity; short-term funding | The region experienced significant pressure on services; the program relied heavily on the voluntary sector; and it began before the pandemic changed service delivery. | Future work should establish outcome measures usable across all services, compare different social prescribing models, conduct long-term follow-up, develop stable funding frameworks, and improve cross-organizational data systems. |
| Hazeldine et al., 2021, UK [122] | Link worker social prescribing, including assessment, navigation, community referrals, and tailored small-scale solutions. | General practitioner practices, community trust hubs, and dispersed voluntary and community sector partners in rural and remote areas. | Adults with complex social needs, long-term conditions, loneliness, and moderate to severe mental health issues. | Perceived: Improved appropriateness of referrals, better understanding of link worker workload, improved mental health support navigation, and variable accessibility of services. | General practitioner support; social prescribing champion; structured supervision; experienced link workers; flexible problem-solving | High-severity referrals; limited training; space constraints; poor referral quality; rural transport barriers | The area was rural, with limited transport; community sector capacity varied widely; and health practices differed across cultures and ways of working. | Priority areas include clear referral criteria, mental-health training for staff, structured support systems for link workers, and numerical estimates of health improvements and service use. |
| Heijnders & Meijs, 2018, Netherlands [116] | Strengths-based social prescribing model delivered by a community well-being organization. | Primary care centers linked with a community well-being organization. | Adults with psychosocial needs, including loneliness, anxiety, and bereavement. | Perceived: Increased self-confidence, self-reliance, well-being, and social interaction; improved perceived health; some participants resumed volunteering. | Trusted referral from general practitioners; strengths-based intake; personalized matching; follow-up coaching; buddy support option | Limited follow-up; mismatched activities; sector differences; privacy concerns; financial constraints | Many participants had recently experienced significant life events; matching between participants and providers was important; activities were mainly daytime, which influenced who could attend. | Research should develop standardized referral procedures, define link-worker skills and training requirements, determine optimal referral timing, include randomized trials, and assess the social value generated for the wider community. |
| Hejlesen and Jensen, 2026, Denmark [48] | Arts on Prescription intervention through the Culture Vitamins for Young Adults program. The 10-week program included approximately three arts and culture activities per week, including ceramics, visual art workshops, group singing, theatre, shared reading, architecture workshops, street art, guided film experience, and jazz sessions. Activities emphasized participation, creative expression, voluntary engagement, and low-pressure involvement rather than artistic performance. | Arts, cultural, and community settings in Aalborg, Denmark, including Ceramics Aalborg, North Urban Art Studio, Nordkraft, Aalborg Theater/Teater Nordkraft, Aalborg Library, Utzon Center, Aalborg city spaces, a cinema, and a jazz venue. Participants were referred by general practitioners or job center case workers, and an Arts on Prescription coordinator participated throughout the program. | Young adults aged 18–30 years with mental health challenges, including anxiety, depression, stress, chronic pain, ADHD, or autism, who had discontinued education or were unemployed and were not currently engaged in education, employment, or training. | Perceived health/social outcomes included improved mental well-being, reduced depressive symptoms, improved mood, reduced isolation, increased confidence, strengthened routines, greater self-efficacy, improved social connection, peer support, identity development beyond illness, renewed creativity, and increased readiness for education or employment. Some participants reported concrete next steps, such as applying for education or exploring internships. System-level findings highlighted referral barriers and the need for better post-program transition support. | Voluntary participation; low-pressure creative activities; supportive Arts on Prescription coordinator; hands-on arts engagement; group-based peer connection; shared lived experiences; safe and caring group environment; structured routine; opportunities for achievement and self-expression; flexibility to take breaks; creative variety; familiar interview and program settings; person-centered pacing. | Fatigue and low energy after demanding activities; guilt about non-attendance; anxiety or discomfort in boundary-testing activities such as theatre; inconsistent times or locations; insufficient content warnings for potentially distressing material; limited time for relationship-building; dropout affecting group motivation; referral barriers through job centers; inconsistent professional awareness of the program; limited follow-up and transition support after program completion. | Conducted in the Danish welfare and jobcenter context, where young adults with mental health challenges may experience disrupted education, unemployment, social isolation, and developmental "limbo." The intervention responded to rising mental health needs among young adults and aimed to support psychosocial well-being and readiness for life transitions through arts, culture, and social connection. | Future research should include participants who disengage or do not complete the program, use one-to-one interviews to capture divergent experiences, examine long-term outcomes, assess how different art forms affect well-being, strengthen referral pathways, test structured follow-up after program completion, and explore how Arts on Prescription can support transitions into education, employment, or sustained community engagement. |
| Hoffmeister et al., 2024, Portugal [112] | Primary care social prescribing delivered by social-worker link workers including holistic assessment, tailored action plans, and community referrals. | Two Family Health Units in Lisbon, in partnership with public, private, and voluntary sector organizations. | Individuals of all ages with non-clinical needs, including migrants, isolated older adults, and those experiencing financial or housing issues. | Perceived: Improved collaboration across sectors, more holistic care, quicker problem resolution, clearer definition of the link worker role. | Holistic care focus; motivated staff; culturally adaptable tools; visible link workers; regular meetings; intersectoral networks | Limited resources; low awareness; poor adherence; staff shortages; weak communication; long waits; no follow-up | The population had high socioeconomic vulnerability; local infrastructure was limited; there was no extra funding for the program; care was based on biomedical norms; link workers covered several health units. | Evidence gaps include participant-level outcome data, understanding reasons for dropout, strengthening training and capacity for cultural and health staff, using simple monitoring tools, assessing scalability to other regions, and integrating patient perspectives. |
| Hoffmeister et al., 2026, Portugal [146] | Primary-care social prescribing program delivered through link workers who were trained social workers. The model involved referrals from general practitioners, family nurses, or psychologists; social prescribing appointments; participatory development of individual care plans; linkage to community and social supports; and follow-up with patients, referring professionals, and community organizations, as needed. | Two Family Healthcare Units in downtown Lisbon, Portugal. The program linked patients to community and social resources such as Senior University classes, Portuguese-language classes for migrants, medication support, food and housing assistance, aqua aerobics and other physical activities, day centers, trishaw rides, social and financial support, and other community services. | Patients of all ages with non-clinical needs affecting health and well-being, including older adults in isolation, recently arrived migrants, unemployed adults with low education, people experiencing homelessness, individuals with chronic disease, people needing functional support, and those with social, financial, mental health, or behavioral needs. | System/referral outcomes: 1,298 referrals were analyzed. Main referral reasons were social and financial support, social isolation, unemployment, mental health concerns, functional dependency, and sedentary lifestyle. Social isolation, mental health, and a sedentary lifestyle formed a co-occurring cluster. COVID-19 changed referral patterns, with increased social/financial support and functional dependency referrals and reduced sedentary lifestyle referrals. The study did not assess patient follow-up, adherence, or health outcomes after referral. | Bottom-up implementation; integration into primary care; link worker role performed by social workers; collaborative care planning; free or low-cost community responses; follow-up contact; involvement of general practitioners, family nurses, psychologists, social workers, social services, community organizations, and the voluntary sector; ability to identify referral profiles and adapt responses over time. | Lack of outcome data; reliance on healthcare professionals' referral assessments; exclusion of self-referrals from the analyzed dataset; absence of direct socioeconomic indicators such as income, education, or deprivation; potential reporting bias; limited generalizability beyond two Lisbon Family Healthcare Units; cross-sectional design preventing causal inference; referral-level rather than patient-level analysis. | The program was launched in 2018 as the first social prescribing program in Portugal and operated through the COVID-19 pandemic. Referral patterns reflected changing population needs, socioeconomic disruption, limited healthcare access, pandemic restrictions, and evolving awareness of social prescribing among professionals. | Future research should examine patient trajectories after referral, adherence to prescribed activities, intervention outcomes, links between referral profiles and specific prescribed services, longitudinal referral trends, equity of access, and the long-term impact of social prescribing on health behaviors, chronic disease management, social isolation, and healthcare use. |
| Holding et al., 2020, UK [107] | Link worker social prescribing with co-produced twelve-week plans, signposting, accompaniment, and volunteer support. | Thirty-seven community localities coordinated by the British Red Cross, with referrals from health and statutory sectors. | Adults at risk of loneliness, including young parents, bereaved individuals, and people with mobility limitations. | Measured: Not reported. Perceived: Improved social engagement, confidence, and relationship-building. | Skilled link workers; flexible tailoring; creation of new groups; person-centered support | Short program; complex referrals; volunteer shortages; long waiting lists; transport issues; health limitations | The program covered a wide geographic area; community services had been reduced due to financial cuts; implementation relied strongly on local networks. | Future research should measure long-term loneliness outcomes, assess how much support individuals require after program completion, explore how poverty and deprivation influence participation, and examine investment models for rebuilding community groups. |
| Holt, 2020, UK [102] | Twelve-week arts-on-prescription workshops, including visual arts and crafts. | Community arts venues linked with primary care. | Adults with anxiety, depression, low mood, chronic pain, and isolation. | Measured: Improved well-being and reduced tension-related arousal; short-term mood improvements. Perceived: Feeling calmer, less stressed, and more engaged. | Skilled arts practitioners; supportive group environment; structured sessions; continuity | No comparison group; temporary well-being dips; missing data | The program was delivered inside an existing national social prescribing pathway; the model used group-based activities; participants often experienced social isolation. | Key gaps include long-term studies, evidence on effects on GP consultations, analysis of which program elements drive improvements, and outcome tools that capture participants' lived experiences. |
| Hou et al., 2026, England [73] | Proactive, integrated social prescribing and holistic case-management model delivered through the Central Locality Integrated Care Service (CLICS). The intervention combined community connectors, clinical assessment, personalized care planning, multidisciplinary review, and referral or linkage to community-based assets and services addressing social and clinical needs. | Twenty-five inner-city general practices in Bradford, Yorkshire, England; community connectors were embedded within general practice settings and worked alongside general practitioners, advanced nurse practitioners, and administrative staff. | Adults in deprived and ethnically diverse inner-city areas, including proactively identified at-risk groups such as carers, new asylum seekers, people with dementia, frailty, COVID-19 shielding status, multiple clinical risk factors, and patients opportunistically identified by general practices as needing support. | System-level measured outcomes: reduced unplanned hospital admissions and reduced unplanned accident and emergency attendances compared with matched controls. Equity outcomes: intervention effects varied by ethnicity, with a stronger reduction in admissions among Pakistani heritage patients and a significant reduction in accident and emergency attendances among White British patients. Health/social needs addressed included financial hardship, food insecurity, mental health, disability, frailty, medication review, social isolation, and wider determinants of health. | Embedded community connectors in general practice; proactive identification of at-risk patients; 45-minute holistic needs assessment; personalized care plans; repeated appointments with the same staff; rapport-building; weekly multidisciplinary team meetings; integrated clinical and non-clinical support; knowledge of local community assets; bilingual/community advocacy potential; focus on deprived and ethnically diverse communities. | COVID-19 implementation disruption; inability to distinguish specific CLICS pathways in routine data; higher pre-intervention hospital use among CLICS patients; possible regression to the mean; limited coding of community connector pathway; intervention not fully delivered as planned during lockdowns; inability to identify which components were most effective for which patients. | Bradford is a highly deprived and ethnically diverse city in northern England. CLICS was commissioned as part of the Reducing Inequalities in Communities program, a strategic initiative to reduce health inequalities. The intervention operated in the context of NHS investment in proactive social prescribing and policy interest in reducing health inequalities and avoidable hospital use. | Future research should describe intervention components in greater detail, examine causal mechanisms, investigate why effects differ across ethnic groups, assess long-term effects, evaluate different models of integrated social prescribing, identify the most effective components for different communities, and improve routine data coding of social prescribing pathways and community connector activity. |
| Howlett et al., 2025, Scotland [99] | Green Health Prescriptions including walking, cycling, gardening, conservation, outdoor learning, mindfulness, and gentle movement. | Primary and secondary care, third-sector outdoor providers, and Green Health Partnerships. | Adults with mental health needs, long-term conditions, low activity levels, loneliness, and isolation. | Measured: Staff time and costs per referral. Perceived: Reduced anxiety and depression, improved mood and sleep, increased strength and social connection, and development of new skills. | Knowledgeable link workers; skilled activity providers; broad activity range; equipment support; buddy systems | Weak data systems; distance barriers; limited evening options; low awareness; inconsistent staffing | There were significant differences between urban and rural areas; volunteers played an important role; access to green spaces varied; and provider organizations differed widely in structure. | Priority areas include consistent monitoring tools, financial planning for community organizations, defining core program components, comparing different program models, and tracking participants over extended periods. |
| Ikeda et al., 2026, Hawaiʻi, United States [54] | Culture-based social prescribing intervention centered on restoring a loko iʻa, a traditional Hawaiian fishpond. Activities included clearing invasive vegetation, repairing or rebuilding the kuapā, supporting mākāhā and water-flow restoration, physical work in the fishpond, cultural learning, oli, moʻolelo, and ʻāina-based collective practice. | Two loko iʻa sites on Hawaiʻi Island, one on the east side and one on the west side, delivered through collaboration among community partners, university researchers, retired firefighter liaisons, fire department leadership, firefighter union representatives, cultural practitioners, and kiaʻi loko iʻa. | Active and retired firefighters from the county fire department on Hawaiʻi Island, a high-stress occupational group exposed to traumatic incidents, irregular work hours, PTSD risk, substance-use risk, identity loss after retirement, and limited culturally appropriate mental health supports. | Measured: recruitment, adherence, retention, Brief Inventory of Thriving, and PTSD Checklist for DSM-5. Small, non-significant improvements were observed in well-being and PTSD symptoms. Qualitative: participants reported relaxation, stress relief, mental clarity, spiritual renewal, pride, cultural identity, connection to Hawaiʻi, connection to ʻāina, purpose, camaraderie, relationship-building without rank, alcohol-free peer support, and a buffer between work and home. System/implementation: the study demonstrated the feasibility of culturally grounded, community-led alternatives to conventional mental health support. | Cultural grounding in loko iʻa restoration; connection to ʻāina; Native Hawaiian cultural protocols; moʻolelo and oli; community leadership; firefighter liaisons; involvement of retired and active firefighters; physical outdoor work; peer camaraderie; shared purpose; alcohol-free social connection; convenient east and west island sites; stipend support; union and fire department approval; participatory partnership between community and academic partners. | Scheduling conflicts linked to 24-hour firefighter shifts; unpredictable call-outs and overtime; session cancellations when minimum attendance was not reached; unclear communication about study purpose and expectations; safety concerns related to ocean swells and tool use; travel distance and traffic; variable cultural grounding across sites; mobile sign-in problems due to poor cellular coverage; attrition and adherence challenges. | Conducted in Hawaiʻi after fire department morale was affected by COVID-19-era losses and limited employee assistance options. The intervention was rooted in Native Hawaiian concepts of ʻāina, kuleana, pilina, piko, and mauli ola, and responded to a local need for culturally meaningful, non-clinical well-being support for firefighters. | Future research should use a more comprehensive community-based participatory research approach, integrate loko iʻa staff and firefighter liaisons as full research partners, improve orientation and communication, expand scheduling and site options, strengthen safety protocols, test optimal intervention dose, compare active and retired firefighters, assess effects in a larger powered study, and evaluate scalability to other firefighter and first responder populations. |
| Jensen, 2019, Denmark [103] | Arts on Prescription involving a ten-week cultural program including choir, guided reading, museums, theatre, symphony, creative workshops, and nature activities. | Municipal health and cultural services across museums, libraries, theatres, archives, and nature agencies. | Adults aged 31-49 with mild to moderate depression, anxiety, or stress; unemployed and referred from job centers. | Measured: Not reported. Perceived: Increased energy, self-esteem, motivation, reduced panic symptoms, improved emotional capacity, increased social participation, greater readiness for employment-related steps. | Predictable structure; professional facilitation; supportive non-clinical environment; cross-sector collaboration; strengths-based approach | Initial anxiety; fatigue; difficulty with social re-entry; unclear continuation; group dynamic issues | The program was part of a national pilot; it was delivered by the local government; participants were unemployed adults; and cultural institutions offered various activities. | Future work should produce numerical health and well-being outcomes, establish clear procedures for post-program support, create joint working agreements between the cultural and health sectors, conduct long-term follow-up, and use mixed-methods designs combining qualitative and quantitative evidence. |
| Jensen et al., 2025, Sweden [101] | Ten-week Arts on Prescription delivered twice weekly with two-hour participatory arts sessions. | Referrals from primary healthcare centers to nine cultural institutions. | Adults aged 18 and older with stress, anxiety, mild to moderate depression, loneliness, or chronic illness. | Measured: Improvements in well-being and self-assessed health. Perceived: Better coping, stress management, routines, social support, and reduced loneliness. | Free access; strong primary-care referral links; cross-sector collaboration; person-centered design; relevance for vulnerable groups | No comparison group; small sample; long waits; mental-health delays; language exclusion; high distress | The primary care system had a high mental health workload; many participants were older and socially isolated; activities offered included mindfulness, mediyoga, and nature programs. | Future research should include controlled trials, gather long-term outcome data, analyze differences between men and women, evaluate scalability, define integration routes into formal social prescribing services, and conduct subgroup analyses for older adults and individuals living alone. |
| Jensen & Brorsson, 2025, Sweden [30] | Ten-week Arts on Prescription with structured group sessions combining active and receptive arts practices. | Eighteen primary healthcare centers refer to cultural institutions such as museums and libraries. | Adults aged 18 and older with stress, anxiety, mild to moderate depression, or loneliness. | Perceived: Reduced isolation, increased sense of belonging, stronger social networks, improved confidence, motivation, coping, routines, and daily functioning. | Safe environment; structured schedule; diverse group; cultural-sector collaboration; professional facilitation | Initial anxiety; fluctuating attendance; difficulty maintaining routines; no follow-up | Primary care in Sweden faced time and resource pressures; collaboration with cultural institutions was strong; activities were provided by non-clinical staff rather than therapists. | Priority areas include long-term tracking of participant well-being, evidence linking arts activities to measurable outcomes, studies of transitions after program completion, comparisons with other activity-based interventions, and assessment of whether the model fits within primary-care services. |
| Jensen & Bungay, 2021, Sweden [53] | Participatory arts activities lasting at least six weeks, delivered at least once per week. | Primary care referral into community arts and cultural venues. | Adults aged 18 and older with stress, anxiety, mild to moderate depression, or social isolation. | Perceived: Increased motivation, confidence, hope, activation, reduced loneliness, improved routines; perceived decrease in primary-care demand. | Holistic orientation; practitioner support; positive patient valuation; alignment with non-clinical care pathways | Practitioner time limits; biomedical dominance; low awareness; short-term funding; weak feedback | The mental health burden in the population was increasing; pandemic restrictions increased social isolation; non-clinical activities in primary care were widely available and free of charge. | Evidence gaps involve securing reliable funding for cultural social prescribing, conducting long-term outcome studies, examining ways to involve clinicians who currently do not refer, creating standardized feedback systems, and identifying pathways for integrating social prescribing into routine primary care. |
| Jonsson et al., 2026, Sweden [131] | Four-step Swedish primary-care social prescribing model for loneliness among older adults: routine identification using the question "Are you troubled by loneliness?", in-depth assessment of needs, interests, and ability to engage in social activities, written personalized prescription for a community-based social activity, and structured follow-up after approximately 3 weeks and 3 months. | Ten primary care centers across six Swedish municipalities, including rural and urban centers and public and private providers. Social prescribing was delivered by existing licensed primary care staff rather than dedicated link workers. Prescribers included mainly occupational therapists, but also nurses, psychologists, assistant nurses, rehabilitation coordinators, and healthcare counselors. | Older adults aged ≥65 years who were troubled by loneliness, interested in trying social prescribing, and willing to participate in the research. The patient interview sample included 16 older adults aged 69–92 years. | System/implementation outcomes: implementation fidelity was weak. The loneliness screening question was used selectively rather than routinely, assessment and prescribing processes were not always person-centered, follow-up was inconsistent, and some patients experienced disappointment or distress when expectations were raised but support or follow-up was not delivered. The study did not assess clinical effectiveness or patient health outcomes. | Alignment with Swedish policy interest in proactive, integrated, health-promoting, and person-centered primary care; prescribers' recognition of loneliness as an important health issue; professional fit with occupational therapy; written model materials; structured assessment and follow-up templates; research-team support during implementation; voluntary engagement of motivated primary care centers. | Selective screening based on assumptions about who appeared lonely; discomfort asking directly about loneliness; time pressure in clinical encounters; medicalized primary care routines; symbolic rather than active managerial support; lack of clear organizational mandate; competing clinical and statutory tasks; poor workflow integration; limited prescriber knowledge of local activities; weak primary care–community links; lack of suitable or meaningful social activities, especially in some local areas; patient transport, mobility, financial, emotional, and psychological barriers; inconsistent follow-up. | Conducted in an emerging Nordic social prescribing context where the model was being tested rather than routinely embedded. The intervention was developed to address loneliness among older adults and to align with Swedish health system reforms toward proactive, person-centered primary care. Implementation depended heavily on local organizational capacity, managerial engagement, prescriber discretion, and the availability of accessible community resources. | Future work should clarify which model components require high-fidelity delivery, strengthen leadership and managerial accountability, embed social prescribing into routine primary care workflows, develop formal bridging structures between primary care and community resources, improve local activity mapping, strengthen person-centered matching, add practical transition support such as buddying or transport assistance, and evaluate patient outcomes only after feasibility and implementation fidelity have improved. |
| Kellezi et al., 2019, UK [9] | Holistic social prescribing pathway including general practitioner referral, health-coach assessment, link-worker support, and referral to community groups. | General practitioner practices and community and voluntary organizations within the East Midlands pathway. | Adults with chronic illness and loneliness or social isolation. | Measured: Larger group membership, stronger belonging, reduced loneliness, decreased primary-care use. Perceived: Increased confidence, motivation, sense of support, and well-being. | Supportive link-worker relationships; personalized contact; accompaniment; general practitioner advocacy; welcoming community groups | GP knowledge gaps; inconsistent feedback; unwelcoming groups; anxiety; high referral volume; inconsistent follow-up | The area was relatively affluent with strong community resources; the primary care population was large; referral patterns differed between general practitioners. | Key gaps include long-term follow-up, evaluations in deprived areas, improved group-matching processes, clearer theoretical models explaining program effects, and structured communication between referrers and program staff. |
| Kiely et al., 2024, Ireland [77] | Practice-based link worker social prescribing model including needs assessment, goal setting, personalized resources, and one-month follow-up. | General practitioners practice in deprived areas, using a mix of in-person, telephone, and online delivery. | Adults with multimorbidity and psychosocial or socioeconomic challenges. | Measured: Just over half of participants achieved their personal goals; improved self-rated health among those with goal achievement. Perceived: Increased confidence, better routines, feeling listened to. | General practitioner endorsement; co-located link workers; person-centered communication; flexible contact; digital support | Short duration; literacy barriers; pandemic limits; high needs; digital exclusion; limited community options | The area was deprived; pandemic restrictions influenced delivery; link workers came from varied backgrounds; the program duration was pre-set by the trial design. | Evidence gaps relate to flexible program length, recruitment embedded in routine care, tailored support for people with complex needs, stepped wedge or phased trial designs, strategies for including individuals with limited digital access, and clearer definitions of worker roles. |
| Kiely et al., 2024, Ireland [147] | Practice-based link worker model including needs assessment, one-month follow-up, motivational interviewing, and connection to community supports. | General practitioners practiced in deprived urban areas, mostly providing remote care during the pandemic. | Adults aged 18 and older with multimorbidity, high treatment burden, psychosocial stress, and low socioeconomic status. | Measured: No significant change in health-related quality of life or symptoms of anxiety and depression; more participation in activities; slight well-being improvement for men; intervention not cost-effective in base analysis. | General practitioner engagement; link worker retention; behavioral-change training; structured supervision; flexible delivery | Pandemic restrictions; under-recruitment; low contact; literacy barriers; missing data; multimorbidity; limited services; short duration | The study took place during strict lockdowns; the setting was a deprived urban area; psychosocial stress was high; community infrastructure was limited; program duration was fixed and short. | Priorities include longer program durations, evaluations free from pandemic constraints, long-term cost assessments, improved recruitment of socially vulnerable groups, stronger study designs, and development of a core outcome set for social prescribing research. |
| Loftus et al., 2017, Northern Ireland [127] | Twelve-week community activity referral program including social clubs, arts, exercise, befriending, and learning activities. | General practitioner referral to community programs through the Health and Social Care Trust. | Adults aged 65 and older with chronic conditions, social isolation, and frequent healthcare use. | Measured: No change in visits to general practitioners; moderate engagement with activities. Perceived: Reduced isolation and improved emotional well-being. | Coordinator-led referrals; general practitioner explanation; home assessments; broad activity options; established community partnerships | Low uptake; delays; health decline; loss of motivation; short program | Commissioning resources were limited; the program was delivered in a single practice; the population was mainly older adults with multiple conditions; social prescribing was framed to reduce general practitioner workload. | Future research should identify predictors of program uptake, determine which components influence healthcare utilization, use larger studies with adequate statistical power, and reduce waiting times between referral and first contact. |
| Ma et al., 2025, USA [108] | Five-week virtual group-based social prescribing intervention including network mapping, goal-setting, social skills, and behavioral activation. | Urban academic primary care network of sixteen clinics using virtual video delivery. | Young adults aged 18-25 with elevated loneliness and diverse identities. | Measured: No significant improvements in loneliness, depression, or anxiety. Perceived: Increased confidence, emotional awareness, self-compassion, proactive social engagement, and normalization of loneliness. | Supportive facilitators; inclusive group environment; relevant content; virtual accessibility; incentive structure | Limited recruitment; virtual barriers; burdensome tasks; low engagement; small groups; short sessions | The program adapted social prescribing to a U.S. primary care setting; loneliness was shaped by life-stage transitions; the virtual format influenced group interaction. | Priority areas include larger trials, comparisons of online versus face-to-face formats, identification of the most effective program components, studies explaining mechanisms of change, long-term tracking, and evaluation of scaling across primary-care settings. |
| Makanjuola et al., 2022, UK [70] | EmotionMind Dynamic lifestyle coaching delivered through six face-to-face sessions or online guided self-help with group sessions. | Primary care referral pathway with community and online delivery. | Adults aged 18 and older with anxiety, stress, trauma-related conditions, or depression. | Measured: Improved well-being and self-efficacy; small reduction in mental health-related service visits; reported positive economic return. Perceived: Better confidence, emotional balance, coping, and motivation. | Strong coach relationship; structured model; flexible delivery; empowerment-aligned approach | Recruitment difficulty; limited individual contact; small sample; no comparison group; recall bias; uneven follow-up | The program shifted online due to pandemic restrictions; there was high demand for mental health services; the area had moderate deprivation; and referral pathways varied across services. | Evidence gaps include feasibility testing with larger samples, refining the online model, conducting long-term follow-up, involving a broader range of stakeholders, and conducting a more detailed economic evaluation. |
| McGarity-Shipley et al., 2025, Canada [74] | Link worker social prescribing model embedded in primary care. A social prescribing link worker from a local community resource center was integrated into a rural family health organization to provide mental health support, community resource navigation, and connection to non-clinical services addressing social determinants of health, isolation, and well-being needs. | A rural family health organization in the Ottawa region, Canada, was delivered through collaboration between a community resource center and a primary care clinic. | Rural primary care clients with mental health, social isolation, social determinants, or community resource navigation needs. | Measured: well-being, loneliness, and healthcare utilization. Well-being improved by 43% and loneliness improved by 23% among the small subset with repeated measures. Hospital, emergency department, and paramedic use decreased among participants with discharge utilization data, while family doctor visits increased. System: findings suggest improved integration between primary care and community services. | Embedding a link worker in primary care; collaboration between a community resource center and family health organization; local community resource navigation; mental health support; rural community knowledge; integrated care approach; connection between healthcare and community organizations. | Very small number of participants with complete outcome data; limited ability to draw conclusions about effectiveness; rural service access challenges; possible limited availability of community resources; dependence on collaboration between healthcare and community organizations. | Conducted in a rural Ottawa community outside the community health center model of care. The intervention addressed the need for integrated, community-linked support in rural primary care, where social isolation, mental health needs, and limited-service access may affect health and healthcare utilization. | Future research should evaluate the model with larger samples, more complete outcome data, longer follow-up, comparison groups, and stronger measurement of well-being, loneliness, mental health, healthcare utilization, and implementation processes. Further work should examine the scalability of rural primary-care/community resource center partnerships and identify which clients benefit most. |
| McGowan et al., 2026, Ireland [35] | Hospital-based link-worker social prescribing model connecting older adults to personalized non-medical community supports, including community activities, dementia-specific activities, day care centers, befriending services, biodiversity tours, walking groups, choirs, men's sheds, art, knitting and crochet classes, exercise classes, online activity packs, and statutory/community services | Tertiary acute hospital geriatric medicine department at St James's Hospital, Dublin; referrals originated from outpatient clinics or before discharge following acute hospital admission; first contact occurred on the ward, in patients' homes, or in outpatient clinics | Older adults aged ≥65 years with complex medical and social needs, including people living alone, people with dementia or mild cognitive impairment, multimorbidity, social isolation, loneliness, anxiety, low mood, caregiver stress, or need for re-connection after illness or functional decline | Measured: Significant improvements in mental well-being measured by WEMWBS and WHO-5 among program completers; MYCaW well-being and concern scores improved but were not statistically significant. Social: referrals addressed loneliness, social isolation, cognitive stimulation, community reconnection, and access to social/community activities. System: feasible delivery of a link-worker SP pathway within secondary geriatric care; demonstrated hospital-based SP as an operational model for older adults with frailty, dementia, and multimorbidity | Dedicated link worker embedded in geriatric services; link worker knowledge of local services; personalized community referrals; telephone support; advocacy; liaison with primary care teams, public health nurses, general practitioners, social workers, home care package managers, dementia services, community psychiatric teams, and hospital staff; alignment with geriatric medicine's holistic and non-pharmacological ethos | Transport difficulties; poor mobility; burden of appointments; dementia and cognitive impairment; high medical acuity; deteriorating health; hospital readmission; transition to long-term care; death; living outside catchment; COVID-19-related restrictions and social anxiety; digital exclusion due to lack of equipment, internet connection, or digital literacy; long referral-to-contact time | The program was implemented in Dublin's inner city, an area of considerable social deprivation, during the COVID-19 period. Participants were medically complex and differed from healthier community-based SP cohorts. The service was developed in the context of the Irish National Framework for Social Prescribing and growing interest in integrating hospital, primary care, social care, and voluntary/community sectors | Future research should include controlled designs, validated loneliness measures, assessment of hospital and outpatient service use, longer follow-up, evaluation across multiple hospital sites, and more diverse populations, analysis of mechanisms linking SP to well-being, and policy-level work on transport, digital access, community capacity, and commissioning of secondary-care SP pathways for older adults. |
| McMullen et al., 2024, UK [43] | Culturally adapted social prescribing for Pakistani carers, including gender-specific groups, Urdu-language delivery, and community or religious activities. | General practitioner referral pathways, voluntary and community sector organizations, mosques, and community venues. | Pakistani carers aged 18 and older providing high-intensity care; English and Urdu speakers. | Perceived: Reduced isolation, emotional relief, culturally appropriate support, stronger peer connection, improved well-being. | Culturally knowledgeable staff; gender-sensitive sessions; delivery in local languages; mosque-based access; strong community partnerships | Stigma; reluctance to identify as carers; language barriers; low awareness; low referral rates; underfunded services; unclear terminology | The setting had high deprivation; caregiving roles followed gendered norms; religious expectations shaped engagement; mistrust of services was common; interpretation resources were limited. | Future work should develop models tailored for South Asian families, compare outcomes across ethnic groups, strengthen coordination between general practitioners and voluntary organizations, provide staff training for culturally diverse caregiving contexts, and include older carers in research. |
| Mercer et al., 2019, Scotland [31] | Community link practitioner model with individualized support and community referral. | Primary care practices in highly deprived areas with full-time community link practitioners. | Adults with multimorbidity, psychosocial needs, and low income. | Measured: No overall change in well-being or quality of life; improvements among those attending several sessions. Perceived: Better engagement with services and resource use. | Flexible relational support; repeated sessions; practice-development funding; strong engagement | Low engagement; weak GP involvement; deprivation; limited capacity; unclear contribution of specific components | The area had very high deprivation; financial cuts affected community services; voluntary organizations had limited capacity; mental health needs were widespread. | Priority areas include strategies to increase participation, more extended follow-up periods, evidence on program costs, identification of the components driving change, and pre-implementation assessments to determine program suitability. |
| Moffatt et al., 2017, UK [44] | Link worker program including assessment, motivational interviewing, action planning, community referrals, and self-care support. | Seventeen general practitioner practices with voluntary-sector delivery in community venues, homes, and cafes. | Adults aged 40-74 with chronic conditions, multimorbidity, social isolation, or low income. | Measured: Improved physical fitness, weight and diet, and better management of long-term conditions. Perceived: Reduced isolation, improved mood, increased confidence and resilience, and financial benefits. | Trusted link-worker relationships; long-term support; flexible contact; personalized goals | Health setbacks; low confidence; weekday-only access; need for intensive early support | The area had severe deprivation; many participants had multiple health conditions; income insecurity was common; the program was funded through a social impact bond; voluntary organizations were essential for delivery. | Evidence gaps include conducting large comparative studies, validating outcome measures, conducting cost evaluations, determining the levels of support required, and analyzing reasons for disengagement or dropout. |
| Morimoto et al., 2023, Japan [91] | Community-library social prescribing model with consultation support and multiple volunteer roles. | Community library hub staffed by medical and social professionals and volunteers. | Socially isolated residents, including unemployed adults, caregivers, bereaved individuals, parents, and disabled persons. | Perceived: Reduced loneliness, emotional relief, increased empowerment, new skills, greater participation in community life, stronger community connection. | Welcoming space; walk-in access; trusted staff; safe consultation; varied roles; community involvement; long opening hours | Limited diversity; response bias; volunteer dependence; limited reach | The setting was a rural Japanese city with an ageing population; national policy encouraged community integration; having medical staff on-site helped reduce barriers to participation. | Future research should include non-users of the service, examine whether findings apply to other regions, gather long-term outcome data, conduct economic evaluations, and plan how libraries can be formally integrated into social prescribing systems. |
| Morris et al., 2022, UK [78] | Remote link worker social prescribing during the COVID-19 pandemic, including behavior-change support, long-term condition support, and social and well-being referrals. | General practitioner practices and community voluntary organizations; entirely remote delivery during the pandemic. | Adults aged 40-74 with chronic conditions and individuals identified as vulnerable during the pandemic. | Perceived (positive): Reduced loneliness, improved support with long-term conditions. Perceived (negative): Halted progress, increased symptoms, reduced physical activity, worsening mental health. System: Flexible adaptation and strengthened links between general practitioners and community support. | Flexible adaptation; proactive outreach; established relationships; creative remote support; integration with primary care | Digital exclusion; literacy barriers; weak rapport; inconsistent contact; high caseloads; service closures; reduced motivation | The study took place during strict lockdowns; the area faced high deprivation, the voluntary sector was overwhelmed, digital access was unequal, and financial stress affected engagement. | Evidence gaps relate to the inclusion of people with limited digital access, maintaining relationships with link workers during service disruptions, understanding voluntary-sector resilience, comparing remote and in-person delivery, and supporting clients with complex needs. |
| Morris et al., 2025, UK [95] | Emergency department-based link worker social prescribing with single-encounter assessment and referral. | Large teaching hospital emergency department with digital referral pathways. | Emergency department patients of all ages with unmet social, emotional, housing, financial, or well-being needs. | Measured: High engagement; large number of patients assessed; strong participation among the most deprived groups; clear internal referral patterns. Perceived: Better discharge support, reduced staff burden, improved workflow. | Co-design; trained link workers; integrated workflow; digital systems; staff awareness | Limited staff awareness; incomplete data; time pressure; weekday-only model; no follow-up | The emergency department served a highly deprived population; emergency demand was high; the program involved multiple agencies; the health service had a strong focus on reducing inequalities. | Key priorities include long-term tracking after emergency department referral, identifying who completes referrals and who does not, assessing the impact on emergency department demand, developing monitoring tools to detect unequal access, and comparing outcomes with primary-care-based social prescribing. |
| Muhl et al., 2025, Canada [49] | Connector-based child and youth social prescribing model with co-produced social prescriptions for community activities, including arts and culture, physical activity, time in nature, career exploration, practical skills, and entertainment/leisure | Comprehensive pediatric integrated health and social service hub located in a community services center in an underserved inner-city neighborhood in Ottawa, Ontario; delivered by hub-based connectors through in-person, telephone, home, hub, or community-based contact | Children and youth aged 0–17 years with poor social health, including social isolation, loneliness, lack of social skills, limited community participation, financial or transport barriers to engagement, and a poor sense of community belonging; many participants came from socioeconomically disadvantaged, newcomer, racialized, Francophone minority, single-caregiver, and disability-affected families | Health: perceived improvements in confidence, self-determination, autonomy, competence, emotional well-being, and sense of purpose. Social: stronger belonging, trusting relationships with connectors, peer connection, family bonding, community integration, and prosocial behavior. System: supported referral, feedback to the hub team, documentation in electronic medical records, and integration within a pediatric social care model | Child-centered "what matters to you" conversations; co-production of prescriptions; trusted connector relationship; supported referral; flexible meeting locations; bilingual delivery; financial barrier reduction; individualized matching to child interests; caregiver involvement; community partnerships; use of the Circle of Courage as a child/youth-centered framework | Short-term program evaluation; potential selection bias; overlapping roles of program development, implementation, and evaluation teams; single coding; deductive analysis may have limited identification of themes outside the Circle of Courage framework; findings based on one program in one community context | Program was embedded in a community social pediatrics hub serving a highly disadvantaged and diverse neighborhood; strong health equity orientation; families did not pay out of pocket for social prescriptions; children and youth were referred through regular hub appointments after identification of poor social health | Future research should examine whether the Circle of Courage can be applied to other child and youth social prescribing programs, explore long-term outcomes, evaluate what works, for whom, and under what circumstances, and further investigate how social prescribing fosters self-determination across childhood, adolescence, and the life course. |
| Muhl et al., 2026, Canada [62] | Child and youth social prescribing program using a connector model. Children and youth were paired with a connector who explored their needs, strengths, interests, and dreams, co-produced a written social prescription, arranged a supported referral, and provided follow-up. Social prescriptions included arts and culture, physical activity, time in nature, career exploration, practical skills, entertainment, and leisure activities. | Comprehensive pediatric integrated health and social service hub located in a community services center in Vanier, Ottawa, Ontario, Canada. The program was delivered through the former Vanier Social Pediatric Hub, in English and French, and was embedded within a community social pediatrics model. | Children and youth aged 4–17 years with poor social health, including social isolation, loneliness, lack of social skills, limited community participation, financial or transportation barriers to engagement, and a poor sense of community belonging. Families were from an underserved inner-city neighborhood with high socioeconomic disadvantage, including low income, housing insecurity, newcomer status, disability, and racial/ethnic minority status. | Perceived outcomes included improved social connectedness, new friendships, increased participation in community activities, greater confidence trying new experiences, improved family sense of community belonging, satisfaction with connectors and activities, and reduced barriers to engagement. System-level outcomes included provider joy and fulfillment, strengthened links between families and community organizations, and improved community cohesion. The study did not quantitatively measure clinical health outcomes. | Connector role; "what matters to you" conversations; co-production of social prescriptions; supported referral; individualized and child-centered approach; covering costs of activities and transportation; bilingual delivery; flexible meeting locations; rapport-building; feedback loop with the hub team; documentation in electronic medical records; trusted pediatric social hub; community partnerships; health equity focus. | Difficulty finding the right social prescription; trial-and-error matching; complex child health needs; caregiver dependency and caregiver engagement barriers; caregiver mental health, pregnancy, work, single parenting, incarceration, and transport challenges; financial hardship; difficulty completing paperwork; under-resourced team; staff turnover; limited connector capacity; poor responsiveness from some community organizations; burdensome subsidy application processes. | Conducted in Vanier, one of Ottawa's most socioeconomically deprived and diverse neighborhoods. The program was developed because the hub team was already addressing urgent caregiver basic needs and wanted to add a child-centered intervention focused on children's dreams, wishes, social health, and positive community participation. The model reflects social prescribing as upstream, health-equity-oriented pediatric care. | Future research should evaluate child and youth social prescribing using stronger outcome designs, longer follow-up, and more diverse sites; examine what works, for whom, and under what circumstances; assess impacts on loneliness, social health, well-being, healthcare use, family outcomes, and community cohesion; explore cultural adaptation; improve caregiver engagement strategies; and test sustainable funding and staffing models for pediatric social prescribing. |
| O'Connor et al., 2024, Australia [55] | Arts on Prescription at Home: an in-home, participatory arts-based social prescribing model delivered by trained professional artists. The model involved 8 weekly sessions with a person living with dementia and, where desired, their family carer, using visual arts, music, dance, creative movement, or other art forms according to participant preference. | Community aged care service in Sydney, Australia, delivered in participants' homes through an existing aged care provider and funded through government Home Care Packages. Referrals came from care managers, allied health professionals, and the wider community/aged care sector. | Community-dwelling people living with dementia and their informal family carers, particularly those for whom group-based arts programs were difficult due to dementia stage, support needs, mobility, geography, or carer circumstances. | Measured: Improved self-rated health and well-being among people with dementia; improved WEMWBS well-being scores among carers; goal attainment for people with dementia and carers; no safety incidents. Perceived: enhanced sense of purpose, achievement, confidence, engagement, enjoyment, respite for carers, improved carer understanding of the person's abilities, and improved dyadic interaction. System: feasible delivery through existing aged care funding, but limited uptake due to workforce and implementation constraints. | Existing community aged care infrastructure; government funding mechanisms; trained AoP@Home artists; tailored artist education and dementia training; advisory input from people with lived experience; flexible home-based delivery; person-centered choice of art modality; reflective artist notes; good fidelity to the AoP@Home protocol; program manager coordination. | Small sample; COVID-19 restrictions; delays in service roll-out; casual artist workforce; limited availability of trained artists by geography and art modality; difficulty matching referrals to suitable artists; some carers' concern about cost or participant ability to engage; limited reach beyond Sydney; two dyads discontinued before completion. | The model responded to access barriers faced by people with dementia who may struggle to attend group-based community activities. The Australian community aged care system enabled the use of Home Care Package funding, but implementation depended on local service capacity, artist workforce stability, and availability of suitable art modalities. Carers sometimes used sessions as respite rather than participating actively. | Future research should test AoP@Home in larger, more diverse samples, evaluate longer-term outcomes, explore telehealth delivery, examine scalability beyond a single provider/geographic region, compare in-home and group-based arts prescribing models, strengthen referral pathways, assess cost-effectiveness, and further investigate implementation strategies to sustain artist-delivered social prescribing in aged care. |
| Ogrin et al., 2026, Australia [100] | Two co-designed community social prescribing/social connection models. Connect Local used trained community connectors to support older adults to identify meaningful social goals and access existing local activities, groups, and services. Spark used trained volunteers ("Sparkies") to host low-pressure, drop-in, interest-based social connection events and activities for women. | Connect Local was implemented in the Glen Eira local government area of metropolitan Melbourne through partnerships with aged and community care, local council, community services, and the health sector. Spark was implemented through a neighborhood community center in Adelaide, with local businesses, community groups, volunteers, and cross-sector partners. | Connect Local: older adults with chronic conditions who were experiencing or at risk of loneliness, social isolation, and/or depressive symptoms. Spark: midlife women experiencing or at risk of loneliness, with activities later extending through intergenerational and broader community participation. | Health/social: intended outcomes included reduced loneliness, reduced social isolation, reduced depressive symptoms, improved well-being, improved quality of life, social confidence, stronger connection behaviors, and increased access to meaningful local activities. System/community: program learning emphasized community capacity-building, relationship development, new partnerships, changes in provider practices, improved welcoming practices, volunteer development, and policy-level interest in social connection. | Co-design with community members; use of existing community assets; trained community connectors and volunteer Sparkies; asset-based framing; local council support; trusted community center infrastructure; flexible drop-in activities; low-pressure participation; citizen-science data collection; Measurement, Evaluation, and Learning approach; social capital framing; adaptive evaluation; cross-sector partnerships. | Linear funder expectations; pressure to use fixed quantitative outcomes; stigma or discomfort with loneliness screening; transport limitations; venue accessibility; waitlists; changing health status; bereavement; cognitive decline; caregiving responsibilities; difficulty collecting individual-level surveys without undermining participation; evolving program boundaries; different definitions of success across interest holders. | Both programs operated within complex adaptive community systems where interventions, contexts, and outcomes changed during implementation. Connect Local was shaped by funder requirements, aged/community care structures, policy interest in social prescribing, and local community assets. Spark was shaped by community co-design, women's preference for action-based rather than education-based activities, and the trusted role of neighborhood community centers. | Future evaluation should move beyond milestone-based linear logic models and use complexity-informed, stewardship-oriented approaches. Priorities include relational indicators, participant-defined outcomes, social capital measures, system-level ripple effects, adaptive learning cycles, longer timeframes, co-developed evaluation methods, and funder guidance that supports flexible evaluation of evolving community-based social prescribing programs. |
| Oliveira et al., 2026, Portugal [68] | REDE D+ program: a multicomponent social prescribing intervention for type 2 diabetes, involving collaborative goal setting, selection of community-based activities, self-care education, health literacy promotion, behavior change support, SMS reminders, SP passport, pedometer use, and support from a nurse social prescriber | Primary healthcare and community setting in the Lisbon metropolitan area, Portugal; delivered through collaboration among primary care nurses, a nurse social prescriber, volunteers, and community stakeholders over 12 weeks | Adults living with type 2 diabetes mellitus, mostly older adults, many retired, with varying education levels and long-standing diabetes; intervention targeted self-care needs, health literacy, physical activity, diet, diabetes awareness, and social well-being | Health: perceived improvements in diabetes self-care, physical activity, healthy eating, medication management, blood glucose and foot self-monitoring, pain/mobility, quality of life, and well-being. Social: increased peer support, group belonging, new friendships, reduced isolation and loneliness, and stronger social interaction. System: high acceptability and satisfaction with a structured SP pathway linking primary healthcare and community resources | Person-centered tailoring; collaborative goal setting with diabetes nurse and social prescriber; group support; peer learning; trusted nurse social prescriber acting as a bridge between primary care and community; community stakeholder involvement; clear program structure; SMS reminders; SP passport; pedometer; accessible communication adapted to low literacy levels; activities matched to personal goals | Age-related limitations; osteoarticular pain; difficulty participating in walking or movement-based activities; initial difficulty understanding the SP passport; low self-esteem; preconceived beliefs about ability to participate; challenges maintaining motivation and commitment; possible social desirability bias due to prior relationship with the moderator | Intervention was developed as part of a broader mixed-methods program informed by needs assessment, consensus-building, the Fundamentals of Care framework, the Behavior Change Wheel, and the COM-B model. The program operated in a Portuguese primary care context and addressed chronic disease self-management through clinical–community integration | Future research should include larger-scale implementation studies, younger and more diverse T2DM populations, perspectives from nurses, healthcare professionals, volunteers, and community stakeholders, quantitative outcomes on effectiveness, longer follow-up, assessment of sustainability, and refinement of nutrition and emotional management activities to individual needs. |
| Opacin et al., 2026, Australia [63] | Co-designed nature-based social prescribing intervention, Friends in Nature, delivered as an 8-week group program. Activities included forest therapy, forest bathing, gardening, coastal and river walks, birdwatching, picnics, mindfulness walks, sensory activities such as "sniffari" and soundscaping, shared meals, reflective conversations, and optional low-pressure participation in green and blue spaces. | Melbourne, Australia, as part of the EU Horizon 2020 RECETAS project. The intervention was delivered through a partnership with Many Coloured Sky, a trusted LGBTIQA+ refugee and asylum seeker community organization, using relational referral, peer co-facilitation, community-based facilitation, and pre-selected natural locations such as parks, creeks, beaches, rivers, and other green/blue spaces. | LGBTIQA+ asylum seekers and refugees experiencing loneliness, social exclusion, displacement-related stress, visa precarity, cultural and linguistic barriers, minority stress, racism, queerphobia, settlement insecurity, and limited access to culturally safe services. | Measured: loneliness, emotional loneliness, social loneliness, nature connectedness, nature dose, and health-related quality of life. Global loneliness and emotional loneliness decreased significantly, and nature connectedness increased. Health-related quality of life did not significantly change. Perceived outcomes included improved mood, calm, hope, emotional regulation, belonging, chosen family, confidence, language practice, public transport confidence, social connection, cultural exchange, safety, and attachment to local nature. | Co-design with community members; trusted referral through Many Coloured Sky; trauma-informed and culturally sensitive facilitation; peer co-facilitation; shared meals; multilingual and culturally inclusive practices; optional participation; safe group composition; repeated weekly contact; relational check-ins; transport support; participant-led activities; attention to culture, identity, memory, and place; pathways for participants to become future peer co-facilitators. | Small sample; single-arm non-controlled design; missing post-intervention data for some participants; structural precarity beyond the scope of an 8-week program; visa insecurity; displacement trauma; language barriers; racism, queerphobia, and exclusion; transport and accessibility needs; potential researcher-facilitator bias; limited subgroup comparison because of participant safety and confidentiality concerns. | Conducted with a highly marginalized population navigating overlapping forms of exclusion related to sexuality, gender identity, forced migration, settlement, racism, legal uncertainty, and social isolation. The model was embedded in RECETAS, an international project testing nature-based social prescribing for loneliness across six cities, and adapted locally through participatory, culturally safe, community-led design. | Future research should test the model using comparison groups, larger samples, longer follow-up, and implementation evaluations; examine whether reductions in loneliness are sustained beyond 12 months; assess dose and engagement processes; compare outcomes across different marginalized groups and settings; evaluate peer co-facilitator sustainability; and investigate how culturally safe nature-based social prescribing can be embedded within health, settlement, and community support systems. |
| Papon et al., 2026, Spain [75] | Nature-based social prescribing through the Friends in Nature intervention. The intervention included an individual interview followed by nine weekly 2-hour facilitated group sessions in natural spaces, including walks, picnics, group cohesion activities in green or blue spaces, guided visits to natural and heritage sites, birdwatching, nature observation, forest bathing, mindfulness in nature, and outdoor artistic activities. | Community and natural spaces in Barcelona, Spain, were delivered as part of the EU-Horizon 2020 RECETAS project. The intervention was compared with usual care, which involved individual signposting to nature-based activities using the same co-created menu of local nature activities. | Adults aged ≥18 years experiencing loneliness, living in socioeconomically deprived urban areas, and willing to participate in group activities in nature. | Measured: social return on investment, net present value, loneliness, health-related quality of life, capability well-being, healthcare resource use, and participation in other social/group activities. The intervention generated positive social value, with EUR 1.48 returned per EUR 1 invested. Qualitative findings reported reduced loneliness, improved connectedness, improved well-being, sense of belonging, increased trust, confidence to engage in hobbies and leisure activities, and a stronger connection to neighborhoods and local natural environments. | Group-based nature activities; individual interview before intervention; co-created menu of local nature activities; trained facilitators; structured nine-session program; stakeholder involvement; integration with the RECETAS trial; use of natural environments to promote connection; social group membership; opportunities for shared experience; qualitative validation of outcomes; inclusion of broader social and economic value through SROI analysis. | Missing outcome data at 3 months; short intervention and evaluation timeframe; reliance on self-reported healthcare resource use; limited availability of Spanish-specific social value proxies; possible limits to generalizability beyond the Barcelona RECETAS setting; potential group conflicts, discomfort, distress, social comparison, or physical injury during nature activities; uncertainty about persistence of effects over time. | Conducted in socioeconomically deprived urban areas of Barcelona within the RECETAS project, which tested nature-based social prescribing interventions for loneliness across European sites. The study reflects growing policy interest in loneliness, social connection, social determinants of health, nature-based interventions, and economic evaluation of social prescribing beyond standard cost-effectiveness approaches. | Future research should include longer follow-up, assess whether effects persist over time, extend SROI analyses to other RECETAS sites, examine social and emotional loneliness separately, include additional psychosocial and community outcomes, improve local valuation methods for Spain, explore subgroup effects, and assess transferability of Friends in Nature to other populations, settings, and nature-based social prescribing models. |
| Payne et al., 2020, UK [45] | Multi-activity social prescribing including advocacy, physical activity, well-being groups, arts and crafts, social cafes, and volunteering. | Third-sector organization linked with primary care; referrals from general practitioners and other health services. | Adults with social, emotional, lifestyle, or practical needs living in high-deprivation areas. | Perceived: Reduced isolation, stronger social connections, improved confidence and resilience, better routines, more meaningful activity, greater mobility, reduced pain, improved self-care behaviors. | Personalized support; assistance attending first sessions; continuous background support; safe informal environment; structured meaningful activities | Low confidence; fear of attending alone; health instability; caregiving demands; dependency; difficulty navigating welfare | The area had high deprivation; the gateway organization also delivered activities; most participants were older White British adults; community sector funding was uncertain. | Priority areas include long-term prospective evaluations, assessment of pathways focused solely on advice and advocacy, more precise explanations of behavioral mechanisms, and greater inclusion of participants from diverse backgrounds. |
| Pescheny et al., 2018, UK [120] | Navigator-based social prescribing, including assessment, motivational interviewing, personalized support, and up to twelve free community sessions. | Four general practitioner practices working with accredited third-sector providers. | Adults with psychosocial, emotional, housing, financial, lifestyle, or isolation needs. | Perceived: Better readiness for change, feeling listened to, increased confidence and personal control, reduced preference for medication. No measured outcomes. | Trusted general practitioner referral; early navigator contact; person-centered communication; free services; clear explanation; navigator accompaniment | Stigma; expectation of medical solutions; short program; inconsistent provider response; low understanding at referral | The setting had high levels of deprivation; the health system was undergoing restructuring; staff turnover disrupted continuity; participants often expected medical solutions; accreditation requirements shaped provider participation. | Future research should examine individuals who decline social prescribing, document reasons for non-participation, determine whether engagement continues once free sessions end, and strengthen cost evaluation. |
| Pescheny et al., 2019, UK [137] | Navigator-led social prescribing with personalized support and referral to twelve free community sessions across physical activity, arts, social groups, and advice services. | Three-year pilot linking general practitioner practices with third-sector delivery. | Adults with chronic or psychosocial needs, including carers and individuals at risk of type 2 diabetes or chronic respiratory disease. | Measured: Increased physical activity levels across all categories; strongest gains among previously inactive participants. | Continuous navigator support; structured pathway; free sessions; diverse community options; alignment with integrated-care strategy | High loss to follow-up; missing data; no comparison group; short follow-up; system delays; sustainability issues; time constraints | The area was highly deprived with a significant minority ethnic population; the program relied on the voluntary sector; chronic disease was common; data-sharing limitations affected coordination. | Evidence gaps include long-term follow-up, controlled evaluations, standardized data systems, affordability assessments, and tailoring strategies for older adults and individuals with full-time employment. |
| Pola-Garcia et al., 2024, Spain [90] | Formal social prescribing protocol integrated into electronic health records, including asset search tools, diagnostic linkage, social-worker assessment, and structured follow-up. | Public primary healthcare centers across 123 geographical areas. | Primary healthcare patients of all ages; the majority older adults and predominantly women. | Measured: Attendance patterns; self-rated improvement; self-rated satisfaction; reported improvement across physical, emotional, relational, cognitive, and self-care domains. Comparative: Differences in attendance and improvement between men and women. | Electronic health record integration; structured workflow; multidisciplinary teams; strong attendance; high satisfaction | High attrition; registration errors; gender imbalance; low male participation; cross-sectional limits | The region had an aging population; the health system was universal; patterns of healthcare use differed between men and women; there were differences between rural and urban areas. | Priority areas include long-term evaluations, explanations for gender differences in response, consistent recording of social prescribing activity, testing in varied regional settings, and analysis of impacts on gender-related inequalities. |
| Pollard et al., 2023, UK [56] | Link worker social prescribing, including holistic assessment, personalized plans, behavior-change support, and onward referral. | General practitioner practices and voluntary and community sector organizations within an outcomes-based National Health Service contract. | Adults with long-term conditions, including diabetes, in deprived multi-ethnic communities. | Measured: Small reduction in long-term blood glucose level. Perceived: Improved motivation, better daily-life management, increased emotional support. | Person-centered rapport; tailored referrals; champions in general practice | Weak GP integration; low referrals; worker turnover; long waits; pandemic disruption | Funding was based on outcomes; the voluntary sector had limited resources; deprivation in the area made engagement more difficult. | Future research should develop social prescribing models that offer longer-term support, align funding approaches with the time required for behavioral change, examine how poverty and inequality shape engagement, include people who do not participate, and apply tools to assess whether programs reduce or worsen inequality. |
| Pollard et al., 2025, UK [43] | Green social prescribing, including walking and gardening, and gym-based social prescribing coordinated by link workers. | General practitioner practices, community gyms, walking groups, and gardening groups. | Adults aged 40-74 with long-term conditions, including diabetes and multimorbidity. | Perceived: Increased sense of purpose, stronger social connection, improved well-being, group belonging; negative views about gym atmosphere. System: Stable referrals to gym-based programs; inconsistent provision of nature-based activities. | Empathetic link workers; skilled activity leaders; peer support; structured pathways | Fragile green activities; group exclusivity; worker turnover; stigma in gyms | Public funding cuts affected community environments; outdoor spaces were not always stable or readily available; and the goals of community groups did not always align with those of the health system. | Priority areas include securing stable funding for nature-based groups, collecting long-term attendance data, evaluating groups formed through social prescribing, understanding group dynamics and inclusion processes, and identifying which participants benefit most from nature-based activities. |
| Polley et al., 2026, England [50] | Bespoke Armed Forces Community Social Prescribing Service delivered by veteran social prescribing link workers. The model involved personalized action planning, practical support, advocacy, navigation of civilian and Armed Forces-specific services, and connection to community activities, health and social care, housing, financial support, food, heating assistance, mental health support, and social groups. | Cornwall, South West England, delivered by Active Plus and funded by Public Health Cornwall Council. Service users were supported in community locations of their choice, at the Active Plus Health and Wellbeing Innovation Centre in Truro, and occasionally through home visits. Referrals came from general practitioners, primary care social prescribing link workers, secondary care, community mental health teams, VCSE organizations, housing associations, a local naval base, self-referrals, and community professionals. | Members of the Armed Forces Community, mainly veterans but also people related to veterans. Service users included individuals experiencing transition difficulties from military to civilian identity, PTSD-related needs, social isolation, financial hardship, housing problems, physical health needs, domestic abuse, and practical barriers to accessing support. | Perceived health/social outcomes included improved mental health, improved psychological and physical well-being, increased hope and optimism, reduced despair, improved living conditions, access to food and heating support, better navigation of appointments and online systems, increased social connection, and improved ability to engage with civilian and community services. System-level outcomes included evidence that veteran link workers can bridge military and civilian cultures and improve engagement with support. | Veteran status of the link workers; shared Armed Forces identity; understanding of military culture, language, banter, and transition challenges; trust-building; structured "one issue at a time" approach; pragmatic problem-solving; advocacy; standardized and efficient service processes; knowledge of Armed Forces-specific and civilian services; collaboration between link workers; ability to tailor support by service branch and individual experience; focus on determinants of health. | Complex and long-standing needs; PTSD and mental health difficulties; financial hardship; food and heating insecurity; housing issues; social isolation; digital exclusion; difficulty asking for help; stigma and military culture; transition from military to civilian identity; risk of over-supporting service users; administrative burden of verifying Armed Forces status; small qualitative interview sample; limited inclusion of spouses and dependents. | Conducted in Cornwall, which has one of the highest Armed Forces Community populations in England. The service was developed to reduce health inequalities and address wider determinants of health among an underserved population. The model responds to the specific cultural and identity-related challenges of leaving the Armed Forces and navigating civilian services. | Future research should include larger and more diverse samples, include spouses, dependents, and other Armed Forces Community members, assess long-term well-being and service-use outcomes, compare veteran-led and generic social prescribing models, examine risks of over-support and link worker burnout, and evaluate the scalability of Armed Forces-specific social prescribing across other regions. |
| Porter et al., 2023, UK [92] | Social prescribing referrals, both formal and informal, to Men's Sheds. | Community Men's Sheds across England and Scotland. | Older men experiencing loneliness, isolation, bereavement, or disability. | Perceived: Reduced loneliness, improved well being, strengthened identity and purpose, increased peer support. | Good communication; matching to Men's Shed environment; peer support; informal activity structure | Inappropriate referrals; safety concerns; unrealistic expectations; paperwork; resource limits; stigma | Activities were led by volunteers; community groups had varying capacities; demand increased after the pandemic; the model relied on an asset-based approach. | Evidence gaps involve establishing clear and safe referral pathways, understanding the experiences of individuals referred through health services, assessing support from integrated care systems, sustaining volunteer-led groups, and planning for limited space and staffing capacity. |
| Potter et al., 2025, England [142] | Primary care-based social prescribing through the Community Connect program, with social prescribers/Community Wellbeing Coordinators and an interim volunteer telephone-befriending intervention introduced during COVID-19. | Primary care-based social prescribing program in the London Borough of Bexley, co-commissioned by the Clinical Commissioning Group and local authority, with links to voluntary and community sector services, welfare support, practical support, and telephone befriending. | Service users with long-term conditions, multimorbidity, mental health problems, loneliness, social isolation, deprivation, housing or financial difficulties, and complex health or social needs during and after COVID-19. | Perceived: Telephone befriending reduced social isolation, increased self-worth, and improved mental well-being for some service users. Health/social: Service users with multimorbidity, mental health problems, loneliness, and isolation were adversely affected physically, emotionally, and socially during the pandemic. System: Remote delivery maintained some contact but had limited impact for digitally excluded users and those with complex needs; implementation and efficacy were weakened when voluntary and linked services were disrupted. | Rapid adaptation to remote delivery; volunteer recruitment during furlough; matched telephone-befriending relationships; regular check-in calls; commissioner support; flexible attempts to maintain contact with vulnerable service users; multilingual volunteer support. | Digital exclusion; loss of in-person contact; difficulty building relationships remotely; intermittent contact due to limited capacity; staff turnover; lone working; social prescriber exhaustion; voluntary-sector closures; volunteer shortages; referral backlogs; inappropriate referrals; reduced access to community services; escalating complexity of need. | Conducted during and after COVID-19 in the context of NHS backlog, disrupted primary care access, economic crisis, rising mental health need, increasing health inequalities, reduced voluntary-sector capacity, and pressure on community and statutory services. Program effectiveness depended heavily on the availability and stability of linked voluntary and community services. | Future priorities include evaluating long-term post-pandemic effects on social prescribing users and providers, strengthening voluntary-sector capacity, developing equitable hybrid models, improving support for digitally excluded and complex-need service users, clarifying referral pathways, supporting social prescriber workforce development, and examining how social prescribing can remain sustainable during system shocks. |
| Redmond et al., 2019, UK [85] | Arts-on-prescription consisting of eight to ten weeks of creative group activities led by artists. | Community venues receiving general practitioner referrals; voluntary sector delivery. | Adults with mental or physical health issues, including low mood, isolation, bereavement, anxiety, and chronic illness. | Perceived: Improved well-being, reduced isolation, enhanced emotional relief, increased confidence and creativity; no measured outcomes reported. | Accessible voluntary-sector delivery; non-judgmental environment; anonymity; skilled facilitators; structured routine | No formal barriers reported | Financial cuts affected mental health services; the voluntary sector played an important role in reaching underserved groups; many participants had low health literacy. | Future work should clarify how creative activities lead to outcomes, evaluate negative or unintended effects, gather long-term outcome data, determine which subgroups benefit, and explore how creativity supports self-management. |
| Schoultz et al., 2026, United Kingdom [82] | Armed Forces Community-specific broker model of social prescribing delivered by dedicated Armed Forces Social Prescriber Link Workers. The model involved tailored assessment, personalized support, signposting, advocacy, navigation, one-to-one support, and connection to veteran-specific, third-sector, primary care, and community resources. | Primary care and community-based social prescribing services in the United Kingdom, with referrals mainly from Social Prescribing Link Workers and additional referrals from healthcare and community networks. The pilot was funded by NHS Armed Forces Health and the Armed Forces Covenant Fund Trust and delivered through Armed Forces Social Prescriber Link Workers. | Members of the Armed Forces Community, including veterans and individuals with military-related experiences and needs. Common referral reasons included social isolation, mental well-being, housing, finances, physical health, employment, family and community issues, and COVID-19 local support. | Measured: Significant improvement in well-being measured by SWEMWBS from initial to final consultation. Perceived/qualitative: improved access to support, stronger advocacy, better navigation across fragmented services, enhanced trust, culturally competent support, and improved engagement with veterans who may otherwise be difficult to reach. System: Armed Forces Social Prescriber Link Workers acted as connectors between primary care, third-sector services, professional networks, and Armed Forces-specific support systems. | Cultural competence in military and veteran experiences; understanding psycho-socio-cultural differences between military and civilian life; advocacy; care navigation; mentoring; outreach; empathy and active listening; personalized care planning; trust-building; rapport; veteran-specific knowledge; relationships between primary care and third-sector services; ability to challenge poor services and coordinate complex support. | Complex health and social care needs; mental health issues; physical disabilities; financial limitations; housing and employment difficulties; mistrust or reluctance to engage; emotional burden of sharing military experiences; fragmented services; bureaucratic hurdles; lack of Armed Forces-specific training in generic social prescribing; risk to service continuity because of third-sector funding instability; variable follow-up timing and limited attrition data. | The Armed Forces Community has distinct well-being needs linked to military culture, transition to civilian life, social isolation, mental health, employment, housing, finances, and service-related experiences. The pilot addressed a gap in generic social prescribing provision by developing Armed Forces-specific link worker skills and culturally responsive support. | Future research should examine long-term outcomes, use longer follow-up periods, assess representativeness and attrition, compare Armed Forces-specific and generic social prescribing models, evaluate scalability across the United Kingdom, identify which components produce the strongest benefits, and assess sustainable funding and training models for Armed Forces Social Prescriber Link Workers. |
| Skinner et al., 2023, UK [32] | Lifestyle-based social prescribing, including physical activity programs, diet-support groups, and structured follow-up. | Primary care and community programs were delivered remotely during the COVID-19 pandemic. | Adults with pre-diabetes and obesity. | Measured: Improvements in well-being, general health, health service use, and reported positive economic return. Perceived: Higher motivation and engagement. | Consistent follow-up; multiple program options; accessible resources; supportive practitioner relationship | Pandemic limits; low retention; no adherence data; small sample; no comparison group | The pandemic reduced the ability to deliver activities; the program operated within the Welsh Diabetes Plan; social prescribing capacity was reduced. | Priority areas include multi-site evaluations, controlled comparisons, reliable measurement of attendance and participation, long-term health outcome assessments, and cost-effectiveness analyses. |
| Skivington et al., 2018, Scotland, UK [117] | Link worker social prescribing with intensive case management and community referrals. | General practitioners practice in high-deprivation areas, working with voluntary and community organizations. | Adults with health problems linked to socioeconomic disadvantage. | Perceived: Improved access to community resources, stronger case-management support, greater awareness among general practitioners of available programs. | Co-location; local knowledge; boundary-spanning role; relational trust | Reduced community capacity; high demand; role overload; organizational turnover | The area experienced financial cuts and welfare reforms; community organizations were overburdened; deprivation was long-standing. | Evidence gaps relate to rigorous outcome evaluations, assessments of long-term sustainability, stronger inter-organizational partnerships, and clarification of social prescribing's role during periods of financial austerity. |
| Stickley & Eades, 2013, UK [104] | Arts on Prescription delivered through community creative groups. | Community arts venues in the voluntary sector linked to primary care mental health pathways. | Adults with depression, anxiety, stress, substance-use history, or social isolation. | Perceived: Higher confidence, stronger self-esteem, more motivation, improved social connection, personal identity development, improved relationships. Observed: Participation in arts education, volunteering, and community leadership roles. | Supportive non-clinical environment; peer networks; long-term participation; creative identity opportunities | Funding cuts, program closures, limited volunteer roles, difficulty sustaining activities, and limited monitoring | The national mental health policy supported arts-based activities; delivery relied on the voluntary sector; financial cuts put pressure on services; participants joined for varied reasons. | Future research should develop methods to measure changes in confidence, identity, and social connection, produce quantitative outcome data, conduct long-term evaluations, assess financial sustainability without external funding, and explore service-user-led approaches. |
| Stickley & Hui, 2012, UK [105] | Arts on Prescription delivered as participatory community arts. | Voluntary and third-sector arts venues with referrals from primary care and mental health services. | Adults with mild to moderate mental health problems, low confidence, social isolation, or complex social needs. | Perceived (referrer views): Improved confidence, motivation, emotional expression, social participation, autonomy; progress toward education or volunteering; reduced reliance on statutory services. | Safe creative space; skilled facilitators; peer support; minimal bureaucracy; complementary to statutory care | Unsuitable for some clients; inconsistent feedback; motivation issues; funding dependence; reduced services; access problems | Mental health care was moving toward community-based models; commissioning decisions were shaped by financial austerity; interest in social prescribing was increasing. | Priority areas include generating evidence considered acceptable by commissioners, undertaking quantitative evaluations, identifying the groups that benefit most, investigating links with primary and secondary care, and conducting long-term follow-up. |
| Sumner et al., 2020, UK [128] | Arts on Prescription involving creative courses delivered by artists. | Primary care social prescribing pathways linked to a community arts program. | Adults referred for mental health concerns, low self-esteem, isolation, chronic illness, or well-being issues. | Measured: Attendance, engagement, change in well-being score; mixed improvement patterns. Perceived: General well-being improvements noted in earlier evaluations. | Established community arts model; long-running scheme enabling consistent participation | Low well-being, reducing engagement; deprivation reducing uptake; multi-reason referrals linked to lower engagement | Studies were conducted in less deprived areas; the model was creative rather than therapeutic; findings came from a single region. | Evidence gaps include determining whether the program directly causes improvements, explaining the mechanisms behind changes in well-being, understanding negative participant experiences, including more diverse populations, and evaluating impacts on social inclusion. |
| Sumner et al., 2021, UK [129] | Arts on Prescription offering visual and performing arts classes. | National Health Service primary care social prescribing in community settings. | Adults with psychosocial or clinical needs, including anxiety, depression, isolation, chronic pain, or bereavement. | Measured: Reduced symptoms of general anxiety, reduced symptoms of depression, improved well-being scores; improvements maintained across multiple cycles. Perceived: Improved confidence, greater social interaction, stronger sense of purpose. | Accessible referral; structured model; re-referral options; professional artist facilitation | Rebound between cycles; inconsistent attendance; difficulty in group settings; anxiety at program end; limited screening | The model was embedded in the national social prescribing system; referrals varied widely; repeat referrals were common; activities focused on creativity rather than clinical therapy. | Future work should include randomized controlled trials, strategies to maintain improvements after program completion, identification of predictors of change, subgroup analyses, evidence on patient perspectives, and clearer guidance on screening and risk assessment. |
| Taylor et al., 2026, United Kingdom [106] | Direct-referral youth social prescribing intervention combining psychoeducation and physical activity. Safety Nets involved weekly 2-hour group sessions over 8 weeks, with one hour of age-appropriate psychoeducation led by CAMHS clinicians and one hour of mild-to-moderate physical activity led by community sports coaches. Sessions covered topics such as peer support, social media, diet, sleep, mental health literacy, and anxiety management. | Local professional sports club stadiums and community sport settings across Yorkshire and Humber, delivered jointly by NHS CAMHS clinicians and sports club coaches. Young people were clinically referred from CAMHS waiting lists to community-based sessions. | Young people aged 11–16 years on NHS CAMHS waiting lists for anxiety, low mood, and/or depression; parents/carers and delivery staff were also included in the qualitative evaluation. | Perceived health/social outcomes included improved mood, confidence, self-esteem, anxiety management, sleep routines, mental health literacy, peer relationships, sense of belonging, social confidence, and school-related functioning. System/implementation outcomes included positive staff experiences, clinician job satisfaction, sports coach professional development, and evidence that social prescribing can be integrated into CAMHS waiting-list support. | Co-production with young people, families, and staff; combination of psychoeducation and physical activity; informal and friendly group atmosphere; non-clinical sports club setting; "pull of the badge" from professional sports venues; co-delivery by clinicians and sports coaches; staff participation in both psychoeducation and physical activity; flexible activities selected with young people; trusting relationships; peer support; experienced staff; balance between structured content and relaxed delivery. | Long CAMHS waiting lists; young people's anxiety, low mood, and depression; travel distance and transport difficulties; program duration perceived as too short by many participants; some psychoeducation topics perceived as repetitive; exclusion or under-inclusion of autistic young people and young people with more complex additional needs; need for experienced staff to manage group processes; uncertainty about scalability and resource requirements. | Conducted in the context of increasing CAMHS referrals, long waiting times, and limited interim support for young people with mental health difficulties in the United Kingdom. Safety Nets was developed as a community-based, non-stigmatizing support option while young people waited for specialist care, linking NHS clinical services with professional sports club community foundations. | Future research should evaluate Safety Nets in a large-scale effectiveness and cost-effectiveness trial, assess long-term outcomes, examine scalability and sustainability, explore adaptations for autistic young people and marginalized groups, consider optimal program length, assess transport and access needs, and provide clearer training and guidance for staff on the role of social prescribing within CAMHS. |
| Thompson et al., 2023, UK [98] | Community Connectors model providing short-term person-centered support and volunteer-assisted signposting. | Thirty-seven locations across home and community settings with multiple referral routes. | Individuals at high risk of loneliness including bereaved adults, retirees, young parents, and people with mobility challenges. | Measured: Reduced loneliness score. Perceived: Higher confidence, motivation, companionship, reduced loneliness during program; some decline after program ended. | One-to-one relationships; personalized plans; consistent contact; volunteer engagement | Short duration; dependency risk; mobility issues; lack of befriending services; few local options; transport barriers; financial constraints | Structural inequalities shaped access: rural areas had limited transport, community infrastructure was weak, and loneliness was a key national policy concern. | Future research should gather long-term outcome data following short interventions, examine how transport barriers, poverty, and limited local services reduce program effectiveness, compare outcomes with similar models such as befriending, assess whether signposting alone is sustainable, and include participants who do not complete the program. |
| Thompson et al., 2026, Australia [37] | General practice nurse-led social prescribing intervention. Nurses acted as health connectors, assessed older people's social networks and interests, used social network mapping and strengths-based conversations, co-produced personalized activity plans, referred participants to community activities and supports, and provided follow-up approximately 12 weeks later. | Seven general practices in the Hawkesbury region of New South Wales, Australia, within the Nepean Blue Mountains Primary Health Network. The intervention was delivered by trained general practice nurses during funded health connector time and linked participants to local community assets, social groups, special interest groups, exercise activities, transport options, health services, aged care, and social care providers. | Community-dwelling older people aged ≥75 years, and Aboriginal and Torres Strait Islander people aged ≥65 years, who were at risk of loneliness and/or social isolation and interested in forming social connections through community-based activities. | Measured: loneliness using UCLA-3, health-related quality of life using EQ-5D-5L, self-rated health using EQ-VAS, activity uptake, and consumer experience. No statistically significant changes were found in loneliness or quality of life, but some participants improved in self-rated health and pain/discomfort. Perceived: increased awareness of community activities, improved social interaction, greater willingness to attend groups, satisfaction with support, and positive overall experience. | General practice nurse role; trusted primary care setting; personalized assessment; social network mapping; strengths-based conversations; motivational interviewing; co-produced activity plans; online community database; follow-up contact; training and refresher training for nurses; project manager implementation support; community of practice; links with health, aged care, social care, and community organizations. | Low recruitment and limited reach; COVID-19 lockdowns and social distancing; bushfires and flooding; disruption of community activities and local infrastructure; transport limitations; limited public transport; stigma around loneliness; difficulty identifying lonely or socially isolated older people; some participants not lonely at baseline; incomplete data capture; inconsistent follow-up timing; poor uptake of recommended activities by some participants. | Conducted in a mixed rural-urban region affected by major bushfires, flooding, and COVID-19 disruptions. The intervention was nested within an asset-based community development initiative and reflected the Australian primary care context, where general practice nurses may be well placed to identify and support older adults experiencing loneliness or social isolation. | Future research should improve methods for identifying lonely and socially isolated older people in general practice, include validated measures of social isolation, assess implementation from nurses' perspectives, strengthen recruitment strategies, address transport and accessibility barriers, examine stigma and uptake, test the model in other settings with less disruption, and evaluate team-based, multicomponent, whole-of-community approaches for older adults. |
| Thomson et al., 2018, UK [8] | Museum-based cultural and creative social prescribing, including talks, tours, object handling, and group arts. | Seven museums in London and Kent receiving referrals from health and social care. | Older adults aged 65-94 at risk of isolation; some with mild to moderate cognitive impairment. | Measured: Improved emotional well-being. Perceived: Stronger social connection, reduced isolation, cognitive stimulation, greater confidence. | Skilled facilitators; meaningful interactions; structured weekly sessions; supportive social environment | Fatigue; travel difficulty; mobility limits; short duration | Museums acted as accessible community resources; delivery relied on volunteers and outreach staff; programs aligned with national well-being policies; inclusion rules influenced who could take part. | Priority areas include long-term follow-up, testing delivery models capable of scaling, conducting economic evaluations, improving accessibility for people with mobility or sensory limitations, measuring physical health outcomes, and evaluating delivery across multiple regions. |
| Tierney et al., 2022, UK [88] | Cultural-sector social prescribing involving gardens, libraries, and museums, delivered with link-worker assessment and signposting. | Cultural venues during the pandemic with many services online or closed; referrals from primary care networks and voluntary organizations. | Older adults aged 60 and older experiencing loneliness or emotional and practical challenges. | Perceived: Gardens promoted calmness and outdoor activity; libraries offered free resources and safety; museums supported learning and social interaction; overall, reduced isolation and increased meaningful activity. | Link-worker familiarity with venues, good communication, and accessible outdoor and indoor settings | Perceived elitism; weak communication; transport barriers; digital exclusion; psychological barriers; closures | Pandemic restrictions limited outreach: link-worker networks varied; workers had different employer arrangements; accessibility standards (e.g., building access) influenced delivery. | Evidence gaps relate to tested partnership models between social prescribing and cultural organizations, identification of groups who benefit most, evaluation of introductory link-worker sessions, strategies to reduce referral bias, understanding sustainability after pandemic-related closures, and economic evaluation. |
| Tierney et al., 2024, UK [46] | Primary care social prescribing delivered by non-clinical link workers providing personalized support, goal-setting, signposting, and coordination. | Seven primary care sites, including general practitioner practices, primary care networks, and voluntary sector subcontractors. | Adults referred for non-medical needs, including isolation, financial strain, housing issues, and low well-being. | Perceived: Improved engagement, feeling heard, stronger person-centered care, higher empowerment; increased link-worker job satisfaction; some emotional burden, and unclear role boundaries. Measured: None. | Flexible roles; supportive supervision; professional autonomy; communication; organizational support | Lack of guidance; unclear boundaries; unrealistic expectations; limited training; high caseloads; housing and financial constraints; time limits; inconsistent supervision; safety concerns | Differences in deprivation and commissioning models shaped delivery; service maturity varied; referral pathways differed; biomedical norms in primary care influenced expectations; voluntary-sector resources were limited. | Future work should clarify appropriate levels of link-worker discretion, provide guidance on safe termination of support, evaluate training and supervision models, investigate staff retention, assess varied role types, examine how professional identity shapes practice, and analyze knowledge-based tasks within social prescribing. |
| Tierney et al., 2024, UK [118] | Primary care link worker social prescribing, including assessment, emotional support, goal setting, signposting, and advocacy. | Primary care sites with general practitioner-embedded or voluntary-sector-contracted link workers. | Adults with non-medical issues, including stress, anxiety, loneliness, housing or financial challenges, bereavement, and caregiving responsibilities. | Perceived: Reduced stress and anxiety, improved coping, increased confidence and hope, reduced loneliness, improved communication, increased social contact, reduced use of general practitioners. Measured: None. | Credible general practitioner referrals; empathetic link workers; personalized plans; strong therapeutic alliance | Transport issues; financial strain; poor housing; few activities; unemployment; low English skills; discomfort in groups; unclear endings; consultation pressure | Social and economic conditions varied across sites; commissioning arrangements differed; voluntary-sector capacity was low; general practitioners had heavy workloads; participant needs were diverse; throughput pressures existed. | Priority areas include collecting long-term outcome data, explaining how relationship quality influences results, studying groups who do not engage with external services, defining clearer training requirements, comparing similar roles, and conducting subgroup analyses such as by gender or socioeconomic status. |
| Tierney et al., 2024, England [81] | Link worker social prescribing model in which link workers identify non-medical factors affecting health and well-being and connect patients to community, voluntary, social, and practical support | Primary care settings across seven sites in England; link workers were based in or connected to general practices and primary care networks, with links to the voluntary, community, and social enterprise sector | Patients referred from primary care with non-medical needs affecting health and well-being, such as loneliness, bereavement, financial issues, housing problems, social isolation, or broader psychosocial concerns; also included link workers, primary care staff, managers, and VCSE professionals as implementation stakeholders | Health/social: patients could receive more holistic, person-centered support for non-medical needs. System: findings focused on link worker integration, legitimacy, referral appropriateness, role clarity, feedback systems, GP workload, primary care–VCSE relationships, and whether link workers were "bolted on," "fitting in," or "belonging" within primary care | Clear explanation of the link worker role; visible presence in general practice; attendance at team meetings; comprehensive induction; supportive supervision; strong interpersonal relationships with GPs and practice staff; time to build VCSE connections; appropriate space to see patients; feedback loops to referrers; recognition of link worker skills; whole-system planning; investment in VCSE capacity | Poor role understanding; ambiguous accountability when link workers were employed across PCN and VCSE structures; lack of supervision; limited feedback from VCSE organizations; pressure to meet throughput targets; insufficient infrastructure; inadequate consultation with stakeholders; lack of professional qualification or legitimacy; underfunded VCSE sector; shortage of space, time, and managerial support | Conducted in the context of the NHS Long Term Plan and national rollout of link workers in English primary care, delivery varied by employment model, geography, deprivation level, number of practices served, and maturity of the link worker role; primary care medical culture influenced whether link workers were accepted as legitimate members of the team | Future work should develop meaningful metrics for link worker impact, strengthen feedback systems between link workers, GPs, and VCSE organizations, clarify supervision and accountability arrangements, examine long-term patient and system outcomes, support VCSE sustainability, and identify how link workers can be fully embedded in primary care without compromising person-centered practice. |
| White et al., 2022, UK [109] | Link worker social prescribing with personalized assessment, tailored planning, signposting, and buddying for first attendance. | Urban general practitioner practices and voluntary organizations with mixed referral routes. | Adults aged 16 and older with loneliness, low well-being, anxiety, depression, inactivity, or psychosocial needs. | Perceived: Higher confidence, emotional support, reduced loneliness, improved awareness of services, better ability to attend community groups. Measured: None. | Strong link-worker rapport; flexible personalized support; co-location; buddying; local knowledge | GP skepticism; low referral rates; low awareness; client anxiety; limited capacity; pandemic restrictions; autonomy-dependency tension | The area had high deprivation; welfare issues were handled by a separate service; community referral patterns differed across providers; general practitioners faced heavy workloads; delivery shifted to phone-based contact during the pandemic. | Evidence gaps include understanding how many clients require more than simple signposting, developing strategies to increase GP referrals, identifying reasons for non-attendance, determining the appropriate duration of support, assessing voluntary-sector capacity for clients with complex needs, and clarifying the role of social workers in social prescribing. |
| Whitelaw et al., 2017, UK [60] | Active social prescribing model with a single point of contact for assessment, matching, referral, and monitoring across eight pathways. | Two rural general practitioner practices with multisector collaboration and an information-technology referral gateway. | Adults with long-term conditions, psychosocial needs, isolation, financial strain, or low well-being. | Perceived: Improved resource access, increased inclusion, improved well-being, stronger partnerships. System: Early integration into practice workflow. Measured: Well-being scores, prescribing counts, referral counts (limited reporting). | Multisector engagement; strong leadership; efficient referral workflow; effective communication; available resources | Unstable funding; confidentiality concerns; no feedback loops; low volunteer capacity; stigma; preference for medical solutions; rural access issues | The setting was rural; practices were early adopters of social prescribing; national well-being policies supported the model; delivery relied on the voluntary sector; funding was short-term. | Future research should evaluate social prescribing in low-resource settings, assess long-term sustainability, develop structured feedback systems, create quality assurance standards for community delivery, study how to manage patient expectations, and improve the detail in outcome reporting. |
| Whiteley et al., 2025, UK [71] | Nature-based social prescribing including horticulture, green crafts, mindfulness, shared meals, and structured sessions. | Rural farm setting with National Health Service, community mental health, and third-sector partnerships. | Adults aged 18 and older with mild to moderate anxiety, depression, or stress. | Measured: Improvements in well-being, capability, self-efficacy, and social connection; reduced visits to general practitioners; reported positive economic return. Perceived: Greater belonging, calmness, confidence, and new skills. | Supportive instructors; safe environment; meaningful structured activities; co-production; strong partnerships; volunteer support | Small sample; no comparison group; variable attendance; dropout; weather barriers; resource-intensive model; volunteer dependence | Mental health needs increased after the pandemic; the model operated within a charitable-sector framework; activities used natural outdoor environments; funding came from European sources; the NHS sought alternatives to conventional care. | Priority areas include large controlled trials, long-term follow-up, evidence on scalability, analysis of underlying cost assumptions, assessment of outcomes for family members and referrers, clear implementation guidance, and analysis of program mechanisms. |
| Wildman et al., 2019, UK [110] | Ways to Wellness social prescribing model, including motivational interviewing, personalized goals, and long-term support with community referrals. | Seventeen general practitioner practices in deprived areas using clinics, community hubs, and home visits. | Adults aged 40-74 with one or more long-term conditions and high socioeconomic disadvantage. | Perceived: Reduced isolation, improved mental health, higher confidence, better self-management of long-term conditions, healthier behaviors, improved ability to navigate welfare services. | Strong link-worker relationships; holistic support; motivational interviewing; flexible long-term contact | Limited culturally suitable services; worker discontinuity; travel costs; unsafe areas; poor scheduling; low motivation; language barriers; unsupportive environments | The area experienced severe deprivation; financial cuts affected public services; delivery depended on the voluntary sector; participants had complex social and health conditions. | Future work should include longitudinal mixed-methods research, the development of solutions to strengthen continuity of support, the generation of evidence on sustaining behavior change, the evaluation of structural barriers such as transport, housing, and safety, and the clarification of long-term support needs for people experiencing unstable life situations. |
| Wildman & Wildman, 2021, UK [66] | Community health worker-led social prescribing with needs assessment, goal-setting, personalized support, and multi-domain referrals. | Primary care in deprived areas with comparison between intervention and non-intervention zones. | Adults aged 40-74 with long-term conditions including diabetes, respiratory disease, heart failure, coronary disease, epilepsy, and osteoporosis. | Measured: Reduced long-term blood glucose level; stronger effects among individuals with fewer comorbidities and higher deprivation. Perceived: Financial and social pressures reduced capacity for self-management. | Long-term support; community-health worker involvement; primary-care integration | Limited culturally suitable services; low capacity for behavior change in multimorbidity; variable uptake; inconsistent referrals; missing minority data | The population had high deprivation and complex needs; usual primary care was provided by the Clinical Commissioning Group; community resources serving minority groups were limited. | Evidence gaps involve identifying causal effects, generating evidence for minority groups, collecting long-term clinical data, explaining change mechanisms, assessing equity impacts, and comparing practice-level versus individual-level effects. |
| Wildman & Wildman, 2023, UK [97] | Long-term link worker social prescribing with structured goal setting, support across eight domains, and multiple community referrals. | Sixteen intervention general practitioner practices and fifteen control practices in a deprived urban area. | Adults aged 40-74 with long-term conditions including diabetes, chronic respiratory disease, heart failure, coronary disease, epilepsy, osteoporosis, anxiety, or depression. | Measured: Reduced non-elective inpatient costs, largest reductions among high-engagement groups, older adults, and individuals from minority backgrounds. Perceived: Better condition management, improved well-being, stronger motivation, reduced isolation. | Long-term personalized support; strong relationship-building; high engagement | Variable engagement; unclear completion; worker capacity limits; reduced effect in multimorbidity; incomplete ethnicity data | The area had high levels of long-term conditions and deprivation; the model was intensive and well embedded locally; national policy expansion supported social prescribing. | Future work should include detailed analysis of primary and community care use, monitor participant engagement over time, broaden outcome measures, conduct subgroup analyses, explain cost changes, and test the applicability of the model across other primary-care networks. |
| Woodall et al., 2018, UK [119] | Well-being coordinator social prescribing with individual assessment, personalized support, signposting, referrals, and a limit of 6 sessions. | General practitioner-aligned service in a large urban area with strong voluntary and community sector links. | Individuals aged 14 and older with social or non-medical needs across varied socioeconomic backgrounds. | Measured: Improved well-being, social connectedness, self-rated health, reduced symptoms of anxiety and depression; mixed findings regarding use of general practitioners. Perceived: Higher confidence, improved coping and purpose, stronger relationships. | Trusting coordinator relationships; empathetic communication; collaborative goal setting; continuity; strong community networks | Session caps; insufficient duration for complex needs; loss to follow-up; dependency concerns; voluntary-sector fragility; austerity pressures; staff turnover | The program took place in an urban area with strained voluntary-sector capacity; demand for services was high; many referral routes existed; activities were free at the point of access. | Priority areas include controlled evaluation designs, system-wide outcome assessments, subgroup analyses across demographics such as age and socioeconomic status, research on the sustainability of voluntary-sector partners, and outcome data specific to high-need participants. |
| Zhao et al., 2026, South Korea [38] | Multicomponent community- and digitally supported social prescribing intervention. Activities included music storytelling, self-help groups, community gardening, Kalimba learning, flower tea classes, smartphone education, COVID-19 prevention sessions, group exercise, singing, movement, and peer-support activities. Digital support was low-threshold and embedded within face-to-face community activities. | Rural community libraries, welfare centers, community gardens, and local community settings in Heung-eop-myeon and Munmak-eup, Wonju City, Gangwon-do, South Korea. The program was organized by Yonsei University with support from local welfare centers, social workers, public health doctors, community health nurses, volunteers, and instructors. | Rural adults aged ≥65 years, including older adults living alone and those with mild depressive symptoms, who were able to communicate in daily life and participate in community activities. | Measured: social support, loneliness, depression, cognitive function, and life satisfaction. The intervention significantly improved social support and life satisfaction and reduced depression compared with the control group. Cognitive function showed slight but nonsignificant improvement, and loneliness did not show a statistically significant direct intervention effect. System/mechanism findings highlighted social support as a key pathway linking social prescribing to better mental health and life satisfaction. | Culturally tailored activities; use of local community libraries and welfare centers; music storytelling as a core component; peer self-help groups; gardening and hands-on activities; smartphone education to support digital inclusion; local social worker and public health provider involvement; volunteer support; repeated weekly engagement; group-based social contact; accessible community settings; integration of local culture and social networks. | Nonrandomized design and possible selection bias; short intervention duration; relatively small pilot sample; limited ability to assess sustained effects; MMSE may be insensitive to short-term cognitive change; loneliness may be deeply rooted and less responsive over a short period; possible residual confounding; social prescribing not yet implemented nationally in South Korea. | Conducted in super-aged rural communities in Gangwon-do, South Korea, where older adults face high risks of depression, loneliness, poverty, low literacy, social isolation, and limited access to conventional services. The intervention reflected a locally adapted social prescribing model combining community resources, cultural activities, and low-threshold digital support. | Future research should use randomized or stronger quasi-experimental designs, larger samples, longer follow-up, more sensitive cognitive measures, and implementation studies assessing sustainability and scalability. Future programs should examine which components are most effective, tailor activities by gender and literacy, strengthen link-worker or community coordinator roles, and test integration into national healthy aging and rural mental health policy frameworks. |
